# Host nucleases generate prespacers for primed adaptation in the *E. coli* type I-E CRISPR-Cas system

**DOI:** 10.1126/sciadv.abn8650

**Published:** 2022-11-25

**Authors:** Anna A. Shiriaeva, Konstantin Kuznedelov, Ivan Fedorov, Olga Musharova, Timofey Khvostikov, Yuliya Tsoy, Elena Kurilovich, Gerald R. Smith, Ekaterina Semenova, Konstantin Severinov

**Affiliations:** ^1^ Center for Molecular and Cellular Biology, Skolkovo Institute of Science and Technology, Moscow 121205, Russia.; ^2^ Saint Petersburg State University, Saint Petersburg 199034, Russia.; ^3^ Peter the Great St. Petersburg Polytechnic University, Saint Petersburg 195251, Russia.; ^4^ Waksman Institute, Rutgers, State University of New Jersey, Piscataway, NJ 08854, USA.; ^5^ Institute of Gene Biology, Russian Academy of Science, Moscow 119334, Russia.; ^6^ Institute of Molecular Genetics, National Research Center Kurchatov Institute, Moscow 123182, Russia.; ^7^ Division of Basic Sciences, Fred Hutchinson Cancer Center, Seattle, WA, USA.

## Abstract

CRISPR-Cas systems provide prokaryotes with adaptive immunity against foreign nucleic acids. In *Escherichia coli*, immunity is acquired upon integration of 33-bp spacers into CRISPR arrays. DNA targets complementary to spacers get degraded and serve as a source of new spacers during a process called primed adaptation. Precursors of such spacers, prespacers, are ~33-bp double-stranded DNA fragments with a ~4-nt 3′ overhang. The mechanism of prespacer generation is not clear. Here, we use FragSeq and biochemical approaches to determine enzymes involved in generation of defined prespacer ends. We demonstrate that RecJ is the main exonuclease trimming 5′ ends of prespacer precursors, although its activity can be partially substituted by ExoVII. The RecBCD complex allows single strand–specific RecJ to process double-stranded regions flanking prespacers. Our results reveal intricate functional interactions of genome maintenance proteins with CRISPR interference and adaptation machineries during generation of prespacers capable of integration into CRISPR arrays.

## Teaser

RecJ, ExoVII, and RecBCD generate prespacer 5′ ends during primed adaptation in the type I-E CRISPR-Cas system.

## INTRODUCTION

CRISPR-Cas systems are prokaryotic adaptive immunity systems acting against mobile genetic elements (*1*, *2*). Complete CRISPR-Cas systems include one or more CRISPR DNA arrays and CRISPR-associated *cas* genes (*3*, *4*). CRISPR arrays consist of repeats separated by unique spacers (*3*, *4*). The arrays are unidirectionally transcribed from promoters located in an upstream leader sequence, and the primary transcript is further processed into short CRISPR RNAs (crRNAs), each containing a single spacer flanked by partial repeat sequences (*5*, *6*). Cas proteins bound to individual crRNAs form effector complexes, which identify sequences (protospacers) complementary to crRNA spacers and mediate degradation of nucleic acids containing recognized protospacers in a process called CRISPR interference (*7*–*10*).

**Fig. 1.**
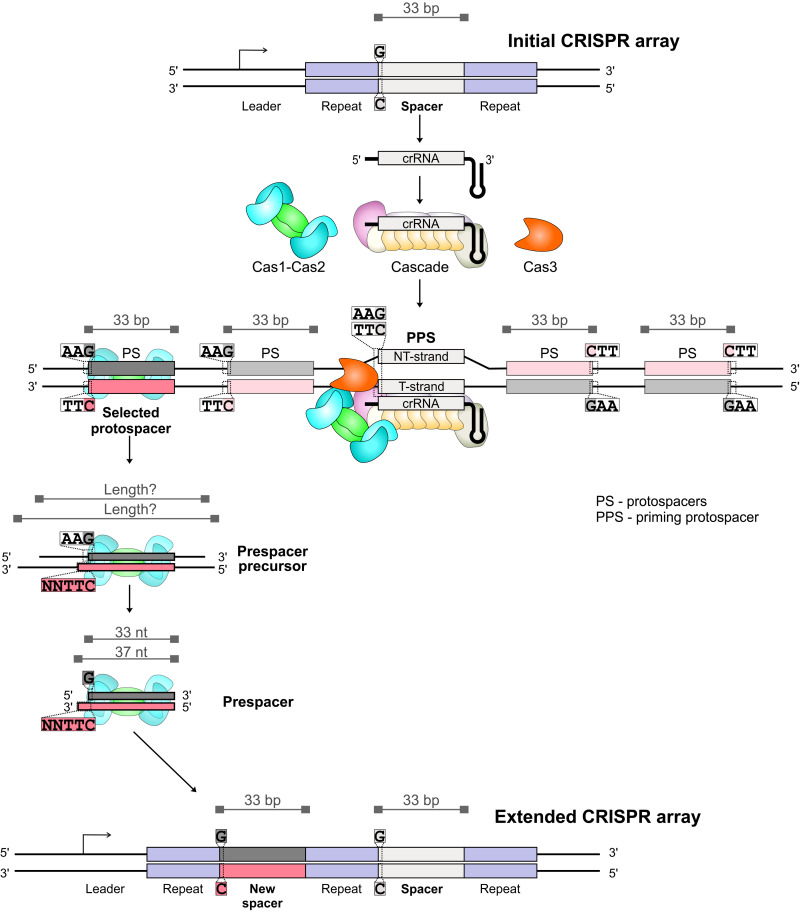
Prespacer generation and integration into the CRISPR array during primed adaptation. A CRISPR array is transcribed yielding crRNA. Cascade bound to the crRNA finds the PPS (priming protospacer) and forms an RNA-DNA duplex with the target strand of protospacer DNA (T-strand). The second PPS strand is called nontarget, or NT-strand. Primed acquisition complex (PAC) composed of Cascade, Cas3, and Cas1-Cas2 selects a new protospacer out of AAG/TTC-flanked 33-bp protospacer sequences specifically oriented relatively to the PPS. The selected protospacer sequence bound to Cas1-Cas2 is excised from DNA as part of a prespacer precursor whose length is unknown. The prespacer precursor is trimmed by cellular nucleases generating a 33/37-nt prespacer. The 3′-NNTT-5′ overhang on the prespacer PAM-derived end is further trimmed, and the prespacer is integrated into the CRISPR array as a new spacer in the correct orientation.

In the type I-E CRISPR-Cas system, the effector is composed of а crRNA and a multisubunit Cascade complex. To be recognized optimally by the effector, the target DNA protospacer must contain, in addition to complementarity with the crRNA spacer part, a 3-nucleotide (nt) motif called PAM (protospacer adjacent motif), which is recognized by one of the Cascade subunits (*10*, *11*). The consensus PAM for the *Escherichia coli* type I-E CRISPR-Cas system is 5′-AAG-3′/3′-TTC-5′ (*12*). When the effector locates its target, the crRNA spacer forms an RNA-DNA duplex with one strand of protospacer DNA, defined as the target strand, or T-strand (Fig. 1) (*9*). Upon recognition and melting of the protospacer by Cascade:crRNA, the Cas3 helicase-nuclease is recruited through protein-protein interactions with target-bound Cascade (*10*, *13*). Initially, Cas3 nicks the nontarget strand of the protospacer (NT-strand), defined as that not complementary to the crRNA (*14*, *15*). Then, it moves away from the protospacer, simultaneously cutting both DNA strands at multiple positions and ensuring highly efficient defense against invaders (*13*–*16*).

New spacers are acquired into CRISPR arrays during a process called adaptation. There are two modes of adaptation: naïve and primed. During naïve adaptation, new spacers are acquired from any DNA source, foreign or domestic, present in cells (*17*). In *E. coli*, about half of newly acquired “naïve” spacers originate from DNA sequences associated with the consensus 5′-AAG-3′/3′-TTC-5′ PAM and can thus support CRISPR interference. During primed adaptation, new spacers are selected from a region surrounding the protospacer targeted by Cascade:crRNA concurrently with degradation of this region by Cas3 (*18*, *19*). Thirty-three–base pair (bp) segments of DNA that are used as a source of new spacers are also defined as protospacers (*12*, *20*). To avoid confusion and to differentiate between several meanings of the term “protospacer,” we will refer to the Cascade-bound protospacer at which primed adaptation is initiated as PPS (priming protospacer) (Fig. 1). Primed adaptation is characterized by a high percentage of newly acquired interference-proficient spacers selected from protospacers with the 5′-AAG-3′/3′-TTC-5′ PAM (more than 95% of all acquired spacers) and their specific orientation with respect to the PPS. Therefore, here, we focus only on such AAG-associated protospacers (Fig. 1).

During naïve and primed adaptation, a complex of two proteins—Cas1 and Cas2—integrates spacer-sized DNA fragments called prespacers at the leader-proximal end of the CRISPR array (*17*, *18*, *21*, *22*). The precise structure of prespacers formed in vivo during naïve adaptation is currently not known. Prespacers formed during primed adaptation have a double-stranded region of ~33 bp corresponding to a mature spacer plus four additional 3′-NNTT-5′ nucleotides on the PAM-derived end (Fig. 1) (*23*, *24*). Therefore, in the present work, we will refer to asymmetrical 33/37-bp fragments or fragments of a very close size and structure containing the 3′-TTC-5′ motif within the PAM-derived 3′ end as prespacers. Integration of such a prespacer starts with a nucleophilic attack by its PAM-distal 3′ end at the leader-repeat junction (*25*). Next, the 3′-NNTT-5′ overhang should be removed to allow for the second nucleophilic attack by the PAM-derived end at the junction site between the first repeat and the preexisting spacer (*26*, *27*).

The mechanism of prespacer generation is not clear. The Cas1-Cas2 complex guided by the PAM sequence captures a wide variety of DNA substrates (*22*, *26*, *28*). We will refer to Cas1-Cas2–bound fragments that are larger than prespacers as prespacer precursors (Fig. 1). According to a current model, the Cas1-Cas2 complex bound to the protospacer sequence within the prespacer precursor determines the boundaries of the future prespacer by protecting it from degradation, while the unprotected ends are removed by nucleases (*26*, *27*, *29*). In some CRISPR-Cas systems, prespacer precursor 3′ ends are generated by Cas4, an auxiliary adaptation protein (*30*–*32*). The *E. coli* type I-E system lacks Cas4. In vitro, *E. coli* exonuclease ExoT or DnaQ can trim the 3′ ends of model substrates mimicking prespacer precursors and generate correct prespacer boundaries (*26*, *27*), but the contribution of these enzymes to prespacer generation in vivo has not been assessed. Likewise, while it was demonstrated that the *E. coli* type I-E Cas1-Cas2 complex protects long double-stranded oligonucleotides from full degradation by the phage T5 5′→3′ exonuclease in vitro (*33*), proteins responsible for generation of prespacer 5′ ends in vivo have not been determined.

To study primed adaptation in the type I-E system of *E. coli,* we previously developed an inducible self-targeting experimental system, in which a spacer matching a sequence elsewhere in the genome is inserted into the CRISPR array (*24*). In a separate study, we demonstrated that inactivation of the host single strand–specific 5′→3′ exonuclease RecJ alone or especially together with RecBCD helicase-nuclease severely decreased primed adaptation efficiency in self-targeting cells (*34*). Here, we study the effect of various *E. coli* exonucleases on generation of prespacers using the self-targeting model and in vitro approaches. We demonstrate that the RecJ 5′→3′ exonuclease is responsible for trimming the 5′ ends of prespacer precursors, although its activity may be partially substituted by ExoVII. RecBCD or its nuclease-deficient RecBC subcomplex enables access of RecJ to the 5′ ends of prespacer precursors with double-stranded extensions, which explains the previously reported strong reduction of primed adaptation in the Δ*recB* Δ*recJ* strain (*34*). Last, we demonstrate that DnaQ and ExoT 3′→5′ exonucleases are not essential for prespacer generation during primed adaptation in vivo.

## RESULTS

### *E. coli* RecBCD and RecJ are jointly required for prespacer generation during primed adaptation in vivo

The self-targeting model system for studying primed adaptation (*24*) is based on an *E. coli* strain with *cas* genes transcribed from inducible promoters and a single CRISPR array with an Sp*^yihN^* spacer targeting a protospacer in the nonessential chromosomal gene *yihN* (table S1). The protospacer in the *yihN* gene has a consensus 5′-AAG-3′/3′-TTC-5′ PAM. There is a single mismatch between Sp*^yihN^* and the *yihN* protospacer immediately to the right of the PAM (as written here). The mismatch stimulates primed adaptation (*18*). Henceforth, we will refer to the *yihN* protospacer as the PPS.

When short single- and double-stranded DNA (dsDNA) fragments purified from 5-hour–induced self-targeting cells undergoing primed adaptation are sequenced and mapped to the chromosome, a distinct peak in the coverage plot is observed around the PPS (*24*). On the basis of the fact that the numbers of fragments originating from the T- and NT-strands are nearly equal and that only double-stranded oligonucleotides can be integrated into the CRISPR arrays (*24*, *35*), we previously inferred that prespacers are double-stranded with a short 3′ overhang on the PAM-derived end (*24*).

In agreement with our previous study (*34*), compared to the wild-type self-targeting strain (*wt*), we observed a decrease in primed adaptation efficiency by ~90% in Δ*recJ* and by ~99% in the Δ*recB* Δ*recJ* derivative strains. We also detected a less prominent ~36% decrease in the ∆*recD* strain (which retains helicase but not nuclease activity) and ~70% decreases in both Δ*recB* and Δ*recC* strains (which lack both activities) (fig. S1, A and B, and tables S2 and S3). In the previous work (*34*), no differences in primed adaptation efficiency between the *wt* and either ∆*recD*, ∆*recB*, or ∆*recC* strains were observed. This discrepancy is likely explained by a more sensitive and quantitative method used to detect spacer acquisition in the current work (high-throughput sequencing versus quantification of agarose gel band intensity used previously). Overall, our observations suggest that (i) host nucleases RecJ and RecBCD participate in prespacer generation and (ii) the decrease in spacer acquisition in mutant strains is caused by reduced prespacer amounts or modified prespacer structures (or both).

**Fig. 2.**
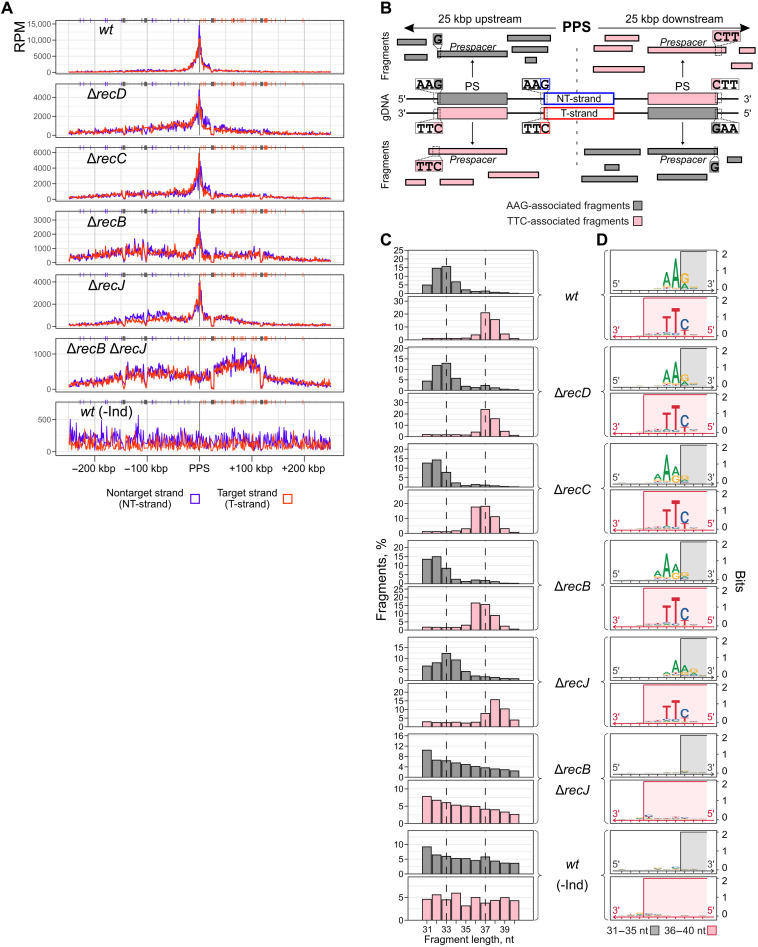
Detection of prespacers in self-targeting *E. coli* strains affected in homologous recombination. (**A**) Normalized sequence coverage of PPS ± 250 kbp [reads per million mapped reads (RPM), in 1-kbp bins] by 31- to 40-nt fragments after adjusting fragment abundancies using fragment length–specific loss coefficients from fig. S2A. Coordinates on the *x* axes show the distance from the PPS. Fragments mapped to the NT-strand are shown in blue; fragments mapped to the T-strand are in red. Chi sites properly oriented to activate RecBCD moving away from the PPS are shown above each coverage plot as blue or red vertical lines. Gray boxes indicate repetitive regions of the genome. Reads matching such regions were excluded from analysis, which leads to an apparent decrease in fragment coverage. (**B**) A 50-kbp region around the PPS is schematically presented. Oppositely oriented protospacers (PS) that are predominantly selected as spacers during primed adaptation are depicted to the left (upstream) and to the right (downstream) of the PPS. Fragments mapping to the NT-strand upstream of the PPS and to the T-strand downstream of the PPS are shown in gray. In the wild type, AAG-associated fragments are found in this group. Fragments mapping to the T-strand upstream of the PPS and to the NT-strand downstream of it are shown in pink. In the wild type, TTC-associated fragments are found in this group. (**C**) Length distributions of 31- to 40-nt fragments originating from the 50-kbp PPS region. One hundred percent corresponds to all 31- to 40-nt fragments from both strands in this region. Fragment abundancies were adjusted using fragment length–specific loss coefficients from fig. S2A. (**D**) Sequence alignments of fragments’ ends and adjacent genomic regions. Gray rectangles represent 5′ ends of 31- to 35-nt AAG-associated fragments. Pink rectangles represent 3′ ends of 36- to 40-nt TTC-associated fragments.

To test these hypotheses, we sequenced DNA fragments purified from *wt*, Δ*recD*, Δ*recB*, Δ*recC*, Δ*recJ*, and Δ*recB* Δ*recJ* strains (table S6). The amounts of fragments were adjusted to account for preferential loss of shorter fragments (Supplementary Text and fig. S2). As expected, enrichment of 31- to 40-nt fragments around the PPS in induced *wt* self-targeting cells was observed (Fig. 2A). The sharpest central part of the enrichment peak was within 50 kilo–base pairs (kbp) around the PPS. In what follows, if not stated otherwise, we analyze fragments located not farther than 25 kbp from the PPS. Similar enrichment peaks were also observed in the coverage plots of all studied mutants except for Δ*recB* Δ*recJ*, suggesting that prespacer generation is abolished when both RecBCD and RecJ are inactivated.

Previously, we found that in *wt* cells most fragments originating from the NT-strand upstream of the PPS (i.e., at the PAM-proximal side of the PPS; Fig. 2B) and from the T-strand downstream of the PPS (i.e., at the PAM-distal side of the PPS) were ~33 nt in length, and their 5′ ends were generated by cleavage of a phosphodiester bond within the 5′-AAG-3′ sequence. We will refer to these fragments as “AAG-associated fragments.” In contrast, most fragments originating from the T-strand upstream of the PPS and from the NT-strand downstream of the PPS were ~37 nt in length, and their 3′ ends were generated by cleavage of the phosphodiester bond 2 nt to the left of the 3′-TTC-5′ motif. We will refer to these fragments as “TTC-associated fragments” (Fig. 2B). Once annealed, complementary AAG- and TTC-associated fragments should produce prespacers with a double-stranded region of ~33 bp and a short 3′ overhang on the PAM-derived end (Fig. 1). The ratios of fragments from the two complementary strands (NT/T) varied in the range of 0.77 to 1.6, suggesting that most fragments have a complementary pair and form double-stranded prespacers (Fig. 2A).

To determine the effects of *recB*, *recC*, *recD*, and *recJ* deletions on AAG-associated fragments, for each strain we combined 31- to 40-nt fragments from the NT-strand upstream and from the T-strand downstream of the PPS (Fig. 2B). Likewise, we combined fragments from the T-strand upstream and the NT-strand downstream of the PPS to determine the effects of *rec* deletions on TTC-associated fragments (Fig. 2B). While in most mutant strains we detected AAG- or TTC-associated fragments with frequencies and distributions the same as or very similar to that of *wt* (see, however, below), no enrichment with AAG- or TTC-associated fragments was observed in the Δ*recB* Δ*recJ* mutant (Fig. 2, A, C, and D). Instead, the distribution of fragments in this mutant was similar to that observed in the uninduced *wt* control, where no detectable adaptation takes place. We thus conclude that the absence of spacer acquisition in the Δ*recB* Δ*recJ* mutant is due to the absence of prespacers.

### The RecBCD helicase and RecJ nuclease participate in the processing of prespacer 5′ ends

Although AAG- and TTC-associated fragments were detected in all studied mutants except for the Δ*recB* Δ*recJ* strain, fragment lengths were slightly different in Δ*recJ*, Δ*recC*, and Δ*recB* mutants compared to *wt* (Fig. 2C). In particular, while 37-nt fragments constituted the major fraction of TTC-associated fragments in *wt*, 38-nt fragments were predominant in the Δ*recJ* strain. Likewise, a higher fraction of the AAG-associated fragments was 1 nt longer in this mutant than in the *wt* strain. On the contrary, higher percentages of fragments 1 nt shorter than those observed in *wt* were detected in Δ*recB* and Δ*recC* strains. The differences in fragment length distributions might be caused by altered processing of the 5′, 3′, or both ends of a prespacer precursor.

To analyze variations in prespacer ends, we first determined chromosomal coordinates of all possible 33-bp protospacers associated with 5′-AAG-3′/3′-TTC-5′ PAMs and located not farther than 25 kbp from the PPS in the correct orientation (375 protospacers with 5′-AAG-3′ in the NT-strand upstream of the PPS and 490 protospacers with 5′-AAG-3′ in the T-strand downstream of the PPS; fig. S3A). The coordinates of such protospacers should (i) coincide with properly processed 33-nt prespacer coordinates (as is the case with 33-nt prespacer strands starting with a G) or (ii) lie within not-fully- processed prespacer coordinates (as is the case with 37-nt prespacer strands having the 3′-NNTTC-5′ motif on their 3′ ends). Last, when a prespacer is processed to a length shorter than the protospacer length (33 bp), prespacer coordinates should lie within the protospacer coordinates. We calculated distances from the 5′ and 3′ ends of all 31- to 40-nt AAG- and TTC-associated fragments to the boundaries of their corresponding protospacers (fig. S3A). Given that in the crystal structure of the *E. coli* Cas1-Cas2 bound to a 33-bp substrate the central 23-bp region is in a double-stranded form, while the terminal 5-nt regions on both sides of the substrate are single-stranded and likely more exposed to degradation (*36*, *37*), we selected for our analysis only those fragments that spanned the central 23-nt protospacer parts (fig. S3A). Negative values were assigned to calculated distances if positions of fragment ends were shifted toward the centers of protospacers; positive values were assigned if positions of fragment ends lay beyond the corresponding protospacer boundaries; zero values were assigned if positions of fragment ends coincided with positions of protospacer boundaries.

The distributions of calculated distances for the *wt* and the mutants are shown in fig. S3B (the Δ*recB* Δ*recJ* strain was not included in this analysis because of low number of PAM-associated fragments and high background of unrelated fragments in the PPS region). For every strain considered, the expected differences in distance distributions for PAM-derived and PAM-distal 3′ ends were observed (fig. S3B). The major fraction of fragments had the 3′-NNTTC-5′ (~60%) or 3′-NNNTTC-5′ (~20%) motif on their PAM-derived 3′ ends, which corresponds to the distance of +4 and +5, respectively. PAM-distal 3′ ends either coincided with protospacer boundaries (distance = 0) or were truncated by 1 nt (distance = −1) in ~60 to 70% of fragments. When we compared the distributions of distances for fragments’ 3′ ends between *wt* and the Δ*recD*, Δ*recB*, and Δ*recJ* mutants, only negligible differences were found (effect size *r* is less than 0.1) (table S7). Therefore, we conclude that none of the nucleases encoded by the affected genes has a considerable effect on the processing of prespacer 3′ ends.

Unlike the asymmetric 3′ ends, PAM-derived and PAM-distal 5′ ends in *wt* were processed symmetrically with respect to the protospacer boundaries (fig. S3B): In ~60% of fragments, both 5′ ends coincided with protospacer boundaries (distance = 0); in ~30% of fragments, an additional nucleotide was present (distance = +1). Both 5′ ends were on average longer in the Δ*recJ* mutant than in *wt* (Mann-Whitney *U* test, *P* ≈ 0; effect size *r* = 0.32) (table S7). Only ~20% of fragments had 5′ ends coinciding with protospacer boundaries, while 50% of fragments had an extra nucleotide (distance = +1) and fragments with two additional nucleotides (distance = +2) became prominent (~20%). Significant differences with *wt* were also revealed in Δ*recB* and Δ*recC* mutants (Mann-Whitney *U* test, *P* ≤ 5.9 × 10^−120^; *r* = 0.23 to 0.37), but in this case, 5′ ends were processed more excessively than in *wt* such that ends located at the −1 distance became prominent (~40% of fragments). In strains lacking RecB or RecC, the shapes of the distributions calculated for the PAM-distal and PAM-derived 5′ ends were different. Specifically, for PAM-derived 5′ ends formed in these mutants, the distance equal to 0 was most prominent, while for PAM-distal 5′ ends, the −1 distance was most prominent (fig. S3B). The result suggests that recognition of the PAM influences the way 5′ ends are processed but only when no RecBCD or RecBC complex is present in the cell.

No changes in 5′ end processing were found in the Δ*recD* mutant. The RecBCD complex, or ExoV, has helicase and nuclease activities on single- and double-stranded linear DNA (*38*–*42*). In the absence of RecD, the remaining RecBC complex has helicase but no nuclease activity (*43*–*45*). Therefore, the helicase activity of RecBCD is required for generation of proper prespacer ends. Together, we conclude that the RecJ 5′→3′ exonuclease activity and the RecBCD helicase activity are involved in the processing of prespacer 5′ ends during primed adaptation.

### Lowered efficiency of prespacer generation rather than modified structure of prespacer ends causes a decrease in primed adaptation efficiency in ∆*recJ*, ∆*recB*, and ∆*recC* mutants

To test whether lowered primed adaptation efficiencies in ∆*recJ*, ∆*recB*, and ∆*recC* mutants are caused by modifications of prespacer ends, we used an oligo electroporation assay (*35*). *E. coli* cells containing a single CRISPR array and expressing *cas1* and *cas2* from a plasmid were transformed with either a 33/37-nt canonical prespacer, a 34/38-nt prespacer with 5′ ends elongated by 1 nt as observed in Δ*recJ*, or a 32/36-nt prespacer with 5′ ends shortened by 1 nt as observed in Δ*recB* and Δ*recC* (fig. S4, A and B, and tables S8 and S9). An amplified mixture of extended and unextended CRISPR arrays in cells harvested 2 hours after transformation was sequenced, and the percentage of arrays containing an oligo-derived spacer was calculated. We found that elongation or shortening of prespacer 5′ ends did not significantly decrease the frequency of oligo-derived spacers or the accuracy of integration (fig. S4, C and D). Similarly, changes of oligo lengths had no or a very small effect on their incorporation into the array of the double *recB recJ* mutant (fig. S4, E and F, and table S10). Therefore, the altered structures of prespacer ends in Δ*recJ*, Δ*recB*, and Δ*recC* do not explain low spacer acquisition efficiencies in these mutants.

**Fig. 3.**
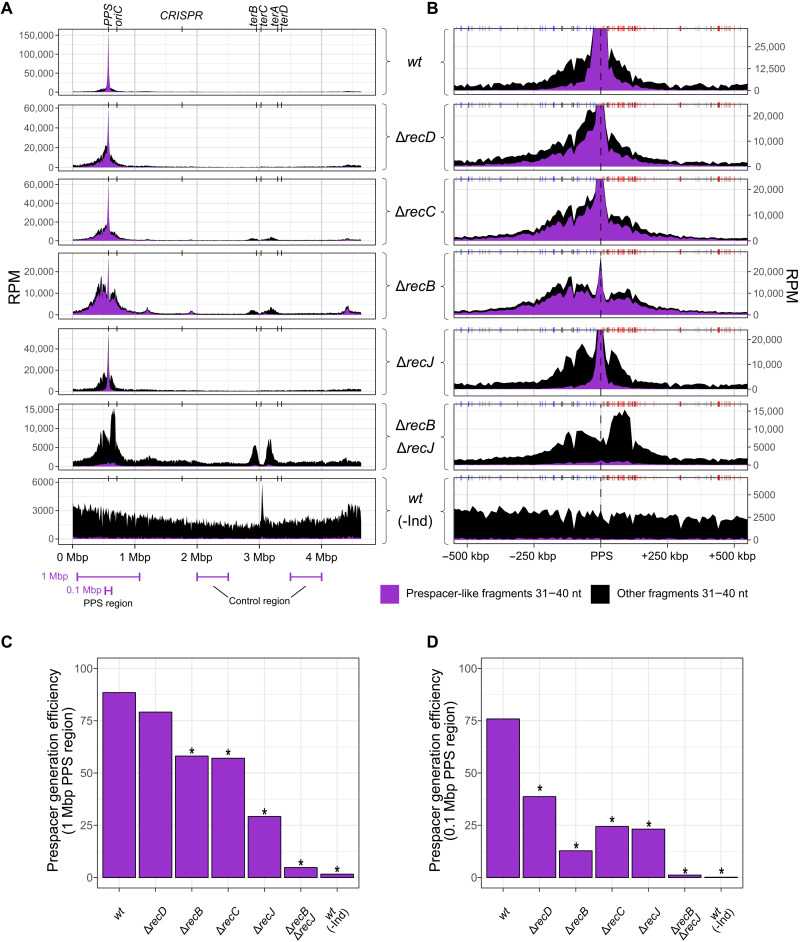
Chromosomal distribution of 31- to 40-nt fragments related and unrelated to prespacers in self-targeting *E. coli* strains. (**A**) Normalized sequence coverage (RPM, in 10-kbp bins) by 31- to 40-nt fragments. Coordinates on the *x* axes represent positions on the chromosome. *oriC*—replication origin; *terA*, *terB*, *terC*, *terD*—sites of replication termination; “CRISPR”—denotes the CRISPR array. Prespacer-like fragments (purple) are defined as 31- to 40-nt sequences overlapping with AAG/TTC-flanked protospacers and having ends located at the following distances from protospacer boundaries: −1, 0, 1, and 2 for 5′ ends; −2, −1, 0, and 1 for PAM-distal 3′ ends; and 2, 3, 4, 5, and 6 for PAM-derived 3′ ends (see fig. S3). All other 31- to 40-nt fragments are shown in black above prespacer-like fragments. (**B**) A close-up of the sequence coverage from (A) shown for the 1-Mbp PPS region (coordinates on the *x* axes represent distance from the PPS). Chi sites properly oriented to activate RecBCD moving away from the PPS are shown above each coverage plot as blue or red vertical lines. Gray boxes indicate repetitive regions of the genome. Reads matching such repetitive regions were excluded from analysis, which leads to an apparent decrease in fragment coverage. (**C**) Prespacer generation efficiency in the 1-Mbp PPS region calculated as the ratio of prespacer-like fragments from the 1-Mbp PPS region to prespacer-like fragments from the 1-Mbp control region. Coordinates of the 1-Mbp PPS and control regions are shown in (A). (**D**) Prespacer generation efficiency in the 0.1-Mbp PPS region calculated as the ratio of prespacer-like fragments from the 0.1-Mbp PPS region to prespacer-like fragments from the 1-Mbp control region. Coordinates of the 0.1-Mbp PPS region and 1-Mbp control region are shown in (A). *P < 0.05 for a χ^2^ test comparing the amounts of prespacer-like fragments in the control and the PPS region between *wt* and other samples.

To estimate the efficiency of prespacer generation in mutant strains, we calculated the amounts of prespacer-like fragments in 1–million base pair (Mbp) region surrounding the PPS. Prespacer-like fragments were defined as 31- to 40-nt fragments whose ends are located at the following distances from protospacer boundaries calculated as in fig. S3: −1, 0, 1, and 2 for both 5′ ends; −2, −1, 0, and 1 for PAM-distal 3′ ends; and 2, 3, 4, 5, and 6 for PAM-derived 3′ ends. We extended the region around the PPS compared to the analysis presented above since in *recBCD* mutants spacers are acquired from a wider region compared to the *wt* (*34*). The number of prespacer-like fragments around the PPS was normalized to the number of prespacer-like fragments in a 1-Mbp “control” region distant from the PPS (Fig. 3, A and C, and table S6). Since this control region is not subject to CRISPR interference, fragments mapping to this region represent a nonspecific background.

When the 1-Mbp PPS-containing region was assessed, the Δ*recJ* mutation led to a ~70% decrease in prespacer generation efficiency (Fig. 3C). Very few prespacers were detected in the Δ*recB* Δ*recJ* strain (a decrease of ~95% compared to the *wt*). Prespacer generation was also decreased, by ~35% in Δ*recB* and Δ*recC*, but not in the Δ*recD* strain (Fig. 3C). The lowered prespacer generation efficiency in the Δ*recD* mutant may be masked by the widening of the area around the PPS where prespacers are generated. Therefore, we also estimated prespacer generation efficiency in a smaller, 0.1-Mbp, area centered at the PPS (Fig. 3D). This analysis revealed a decrease in the amount of prespacers formed in the ∆*recD* strain, which was less prominent than in the *recB* and *recC* mutants. Overall, these results suggest a key role of RecJ in prespacer generation and demonstrate that RecBCD also participates in the process. Therefore, a decrease in prespacer generation efficiency is the likely reason for the low adaptation level in Δ*recD*, Δ*recB*, Δ*recC*, Δ*recJ*, and Δ*recB* Δ*recJ* strains.

Other non–prespacer-like fragments could be generated during the FragSeq procedure or represent specific DNA fragments that exist in a cell. In Fig. 3 (A and B), prespacer-like fragments are indicated by purple color; other fragments are black. While this work is dedicated to prespacer-like fragments, it is evident that non–prespacer-like fragments have specific patterns of accumulation that are affected by *rec* mutations. One notable case of fragments originating from the *ter* region of the chromosome is considered in Discussion.

### RecJ trims single-stranded 5′ ends in the presence of Cas1-Cas2 up to protospacer boundaries in vitro

**Fig. 4.**
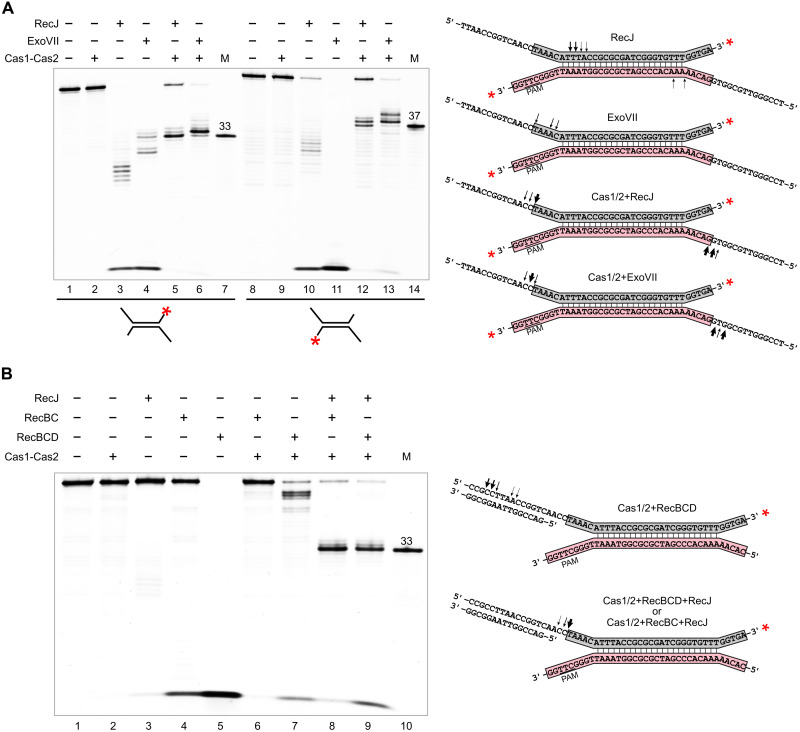
Processing of prespacer 5′ ends by RecJ, ExoVII, and RecBCD nucleases. (**A**) A double-forked DNA substrate composed of a 23-bp central duplex with single-stranded extensions (shown on the right) was labeled at one of the 3′ ends with fluorescein (shown by asterisks) and treated with RecJ or ExoVII exonucleases in the presence or absence of Cas1-Cas2. (**B**) A modified double-forked DNA substrate (shown on the right) was labeled with fluorescein and used to study RecBC- and RecBCD-assisted processing of double-stranded terminal regions by RecJ. Reaction products were resolved by denaturing PAGE. Fluorescein-labeled 33- and 37-nt oligonucleotides (highlighted, respectively, in gray and pink in schemes on the right) were used to map PAM-proximal and PAM-distal boundaries of the DNA substrate protection by Cas1-Cas2 in (A). In (B), only the fluorescein-labeled 33-nt oligonucleotide was used as a marker. Arrows show the positions of cleavage sites observed in the presence of different proteins.

Our in vivo results suggest that 5′ ends of prespacer precursors formed in self-targeting cells are trimmed by the RecJ 5′→3′ exonuclease up to the boundaries of mature 33/37-nt prespacers. To reconstruct this process in vitro, we used a model DNA substrate based on a previously characterized dual forked substrate containing a 23-bp central duplex flanked by 5-nt 3′ and 5′ noncomplementary overhangs (*22*). To achieve stronger binding by Cas1-Cas2 and make the substrate more similar to prespacers observed in vivo (*23*, *24*), we introduced the 3′-TTC-5′ PAM and two additional nucleotides in the PAM-proximal 3′ overhang. To provide the recognition and processing of 5′ overhangs by RecJ (*46*, *47*), we extended both single-stranded 5′ ends to 19 nt (Fig. 4A and table S12).

The affinity of the Cas1-Cas2 complex toward the resulting extended DNA substrate was estimated using electrophoretic mobility shift assay. The Cas1-Cas2 concentration needed to bind essentially all the substrate under the conditions of the experiment was 1.6 μM [a dissociation constant (*K*_d_) of ~0.5 μM] (fig. S5). Parallel reactions containing DNA substrates 3′-terminally labeled at either of the two strands were treated with RecJ in the presence or absence of saturating amounts of Cas1-Cas2. In the absence of Cas1-Cas2, RecJ processed ~30% of substrate molecules to very short fragments that were not resolved on the gel (Fig. 4A, lanes 3 and 10). Approximately 20 to 50% of substrates were digested up to 5 nt within the duplex, reflecting RecJ’s limited digestion within dsDNA connected to a single-stranded 5′ end (*46*). In contrast, in the presence of Cas1-Cas2, digestion stopped at protospacer boundaries on both sides of the DNA substrate, yielding a mature 33/37-nt prespacer and a 33/38-nt version (Fig. 4A, lanes 5 and 12).

The cleavage specificity of RecJ was compared to that of *E. coli* ExoVII exonuclease (a heterodimer of the products of *xseA* and *xseB* genes) that has both 3′→5′ and 5′→3′ exonuclease activities on single-stranded DNA (*48*, *49*). As can be seen from Fig. 4A (lane 11), in the absence of Cas1-Cas2, ExoVII completely digested the bottom strand of the model substrates to products that were not resolved on the gel. Most of the top strand was processed similarly, but some products located at the prespacer boundary (33 nt) as well as shorter (29 to 30 nt) digestion intermediates were observed (Fig. 4A, lane 4). In the presence of Cas1-Cas2, the DNA substrate was stabilized against ExoVII digestion, as expected; however, the digestion products were 1 to 2 nt longer than those generated by RecJ (Fig. 4A, lanes 6 and 13). To check whether the observed effects depend on the substrate sequence, a substrate of identical structure but different sequence was tested. As can be seen from fig. S6, in this case, too, RecJ trimmed 5′ ends up to correct protospacer boundaries (lane 5), while ExoVII produced slightly longer products (lane 6).

The in vitro generation of longer prespacer 5′ ends by ExoVII than by RecJ (Fig. 4A and fig. S6) and detection of prespacers with extended 5′ ends in vivo in the Δ*recJ* mutant (fig. S3B) suggest that ExoVII may be responsible for prespacer generation in the Δ*recJ* strain. To test this hypothesis, we inactivated the large ExoVII subunit encoded by the *xseA* gene (*50*) in *wt* and Δ*recJ* backgrounds. Deletion of *xseA* alone did not influence the adaptation efficiency. In contrast, primed adaptation was markedly (~700-fold) decreased in the Δ*recJ* Δ*xseA* mutant (fig. S1, A and B, and tables S2 and S3). The result is thus consistent with the idea that ExoVII is responsible for nearly all of the residual adaptation in the *recJ* mutant. We also tested the efficiency of acquisition of oligos of different lengths into the CRISPR array of the BL21-AI Δ*recJ* Δ*xseA* strain. No significant changes were observed (fig. S4, G and H, and table S11), suggesting that both RecJ and ExoVII are important for prespacer generation but not for their incorporation into CRISPR arrays.

### RecBCD-assisted processing of double-stranded prespacer ends by RecJ in vitro

Our in vivo data indicate that the RecBCD helicase activity promotes adaptation, but its nuclease activity may be dispensable since nuclease-deficient RecBC promotes prespacer formation (fig. S3). One can envision that when Cas1-Cas2 binds to a prespacer precursor that has terminal double-stranded regions, RecBCD unwinds these regions, enabling access of RecJ to 5′ ends. To test this idea in vitro, we used a double-forked DNA substrate with an extended PAM-proximal 5′ end annealed to a complementary oligonucleotide such that a 15-bp double-stranded segment with a blunt end was formed (Fig. 4B). In the absence of Cas1-Cas2, RecBCD fully degraded this substrate, while RecJ demonstrated very little activity (lanes 3 and 5), suggesting that a blunt end is a suitable substrate for the entry of RecBCD but not RecJ, as expected (*46*, *51*, *52*). RecBC had little effect on the substrate in the absence of Cas1-Cas2 (lane 4), also as expected, as it is a helicase devoid of nuclease activity (*43*–*45*). We attribute the partial degradation of the substrate to low–molecular weight products (Fig. 4B, lane 4) to the small amount of RecBCD contaminating our RecBC preparation, which was obtained during RecBCD purification as a side fraction that lost the weakly held RecD subunit.

When the substrate was bound by Cas1-Cas2, the addition of RecBC had no considerable effect, while RecBCD removed 3 to 4 nt from the double-stranded end, generating a PAM-proximal 5′ end located 15 to 16 nt upstream of the protospacer boundary (lane 7), which is about the distance from the forward end of RecBCD to the nuclease active site (*53*). Thus, RecBCD nuclease is unable to generate 5′ ends of mature prespacers. The addition of RecJ alone to the Cas1-Cas2–bound DNA led to the trimming of the 5′ end up to the prespacer boundary but only in a small fraction of the substrate (fig. S7, lane 4). In contrast, when either RecBCD or RecBC was added to Cas1-Cas2–bound DNA substrate together with RecJ, the substrate was fully converted to a 33-nt fragment whose 5′ end was located precisely at the prespacer boundary (Fig. 4B, lanes 8 and 9, and fig. S7, lane 8). We conclude that the RecBCD helicase unwinds the dsDNA flanking the protospacer bound to Cas1-Cas2, cuts it 15 to 16 nt from the PAM, but allows further digestion by RecJ to produce a correct prespacer.

Since the RecBCD helicase activity is required for prespacer generation, in the absence of RecBCD, other helicase(s) could be involved. One such helicase is RecQ of the RecFOR pathway of homologous recombination, which also includes RecJ (*54*). We generated self-targeting ∆*recQ* and ∆*recB* ∆*recQ* mutants and assessed their primed adaptation efficiency. While the *recQ* mutant was indistinguishable from *wt*, the double mutant was strongly affected (a sevenfold defect compared to the *recB* single mutant; fig. S1, C and D, and tables S4 and S5), indicating that RecQ can contribute to primed adaptation.

### DnaQ and ExoT exonucleases are not required for prespacer generation during primed adaptation

Earlier in vitro experiments showed that *E. coli* exonucleases ExoT (encoded by the *rnt* gene) and DnaQ (encoded by *dnaQ*) can trim 3′ ends of prespacer precursors bound by Cas1-Cas2, producing 3′ ends matching prespacer ends detected in vivo (*26*, *27*). Since no in vivo experiments to our knowledge have been performed with these enzymes, we tested whether prespacer generation efficiency or the structure of prespacer ends is affected by inactivation of ExoT and/or DnaQ. We constructed Δ*rnt*, Δ*dnaQ*, and Δ*rnt* Δ*dnaQ* self-targeting strains and analyzed spacer acquisition efficiency and prespacers generated during primed adaptation (fig. S8 and tables S13 to S17). Overall, prespacer lengths, prespacer ends, and the distributions of distances from prespacer ends to the boundaries of corresponding protospacers in all mutants looked similar to that in *wt* (fig. S8, A to C). In all cases where significant differences were detected by the Mann-Whitney *U* test, the effect size was very small (*r* < 0.1), indicating a negligible impact of DnaQ and ExoT on prespacer ends (table S17). Consistently, when the efficiency of prespacer generation was assessed between *wt* and mutant strains, no differences were found for Δ*dnaQ* and Δ*rnt* Δ*dnaQ* mutants (*P* > 0.05), although prespacer generation was increased ~2-fold in the Δ*rnt* mutant (*P* = 4.3 × 10^−5^) (fig. S8, D to F, and tables S15 and S16), suggesting that its product, ExoT, may destroy some prespacers before their integration at the CRISPR locus. When the adaptation efficiency was assessed, a ~2-fold decrease in spacer acquisition between the Δ*rnt* Δ*dnaQ* mutant and the *wt* was detected (*P* = 0.015) (fig. S8G and tables S13 and S14). Therefore, our results demonstrate that although DnaQ and ExoT jointly contribute to the optimal efficiency of primed adaptation, they are not essential since only a twofold decrease in primed adaptation is observed and no changes in prespacer structures are detected.

### The Cas1-Cas2 complex preferentially binds to complementary pairs of prespacer single strands

Little is known about the early steps of prespacer generation. In vitro, optimal *E. coli* Cas1-Cas2 substrates have symmetrical structures with a central 23-bp duplex flanked by noncomplementary 5-nt single-stranded DNA on each end or by 5-nt 3′ overhangs (*22*). Our data above indicate that prespacers in vivo are asymmetrical 33/37-nt DNA fragments with a blunt PAM-distal side and a 3′ overhang on the PAM-derived side. Unexpectedly, an asymmetric prespacer-like substrate bound Cas1-Cas2 ~40-fold less efficiently than a symmetrical one (fig. S9, B to D, compare substrates 2 and 10; table S18). A 33-bp duplex as well as a 33/35-nt substrate containing the 3′-TTC-5′ PAM also bound poorly (fig. S9, B to D, substrates 8 and 9). In contrast, substrates containing a 28-bp duplex and a single 5-nt 3′ overhang bound the adaptation complex well and increasing the length of the overhang further stimulated the binding (fig. S9, B to D, substrates 3 to 5). These results were obtained with preannealed (i.e., double-stranded) substrates (fig. S9A). Curiously, when the two complementary DNA single strands were added separately to Cas1-Cas2, 5- and 18-fold increases in the binding efficiency of 33/35-nt and 33/37-nt substrates were observed compared to preannealed substrates (fig. S9, A and E). A plausible explanation for this effect could be the formation of a previously characterized dual-forked substrate (*22*, *37*) from completely complementary single strands within the Cas1-Cas2 complex. Permanganate probing showed that the 33/37-nt substrate had permanganate-sensitive (i.e., single-stranded) 3′ ends, when bound to Cas1-Cas2, consistent with the formation of a dual-forked substrate upon binding (fig. S9F). These data support the previous report that Cas1-Cas2 facilitates pairing of complementary DNA strands (*26*) and suggest that Cas1-Cas2 preferentially binds single-stranded prespacer precursors and then quickly promotes the annealing of the missing (complementary) strand.

### Secondary priming detected in CRISPR arrays with a single newly acquired spacer

When we analyzed the distribution of prespacer-like fragments throughout the genomes of various strains, most fragments mapped to the region spanning the *yihN* PPS. However, at least three smaller peaks of fragments were observed in PPS-distant regions (Fig. 3A and fig. S10A). These regions correspond to ribosomal RNA (rRNA) operons *rrnD*, *rrnG*, and *rrnH* (fig. S10A). Since the four remaining rRNA operons *rrnE*, *rrnB*, *rrnA*, and *rrnC* are located less than 150 kbp away from the PPS, new spacers acquired from these four operons during adaptation primed at *yihN* could have led to secondary priming initiated at matching protospacers in the more distant *rrnD*, *rrnG*, and *rrnH* and accumulation of prespacers in regions adjacent to them (fig. S10, A to C). We found new spacers simultaneously targeting several genomic regions in our previously published data (*34*). The largest peaks of these new spacers with multiple alignments (shown in navy blue and maroon in fig. S10C) correspond to rRNA operons. Each of the three PPS-distant rRNA operons to which nonunique new spacers map is surrounded by clusters of uniquely mapped new spacers (shown in light blue and pink in fig. S10C). These unique new spacers map in an orientation expected for primed adaptation, thus implying secondary priming. A similar gradient of uniquely mapped new spacers was also observed for the *fdnGHI* operon located in the chromosomal terminus region and homologous to the *fdoGHI* operon located 19 kbp upstream of the PPS.

All analyzed new spacers, including those that were likely acquired because of secondary priming, were extracted from CRISPR arrays that acquired only a single new spacer (CRISPR arrays with two additional spacers were not analyzed in this experiment because of their low frequency). This seemingly contradicts the requirement of a newly acquired spacer targeting a PPS-distant protospacer to initiate secondary priming. In fig. S10D, we suggest a model to explain this apparent paradox. Because of genome replication, the *ori*/*ter* ratio in a growing bacterial culture is usually about 2, although it can reach higher values under conditions when the rate of cell mass increase surpasses the DNA replication rate (*55*). The *ori*/*ter* ratio in our self-targeting cells is 2.41 ± 0.07 (*24*). The CRISPR array is located ~1 Mbp away from the *oriC*. The CRISPR/*ter* ratio in our self-targeting cells is 1.64 ± 0.08 (*24*). This means that ~60% of cells should have two CRISPR copies simultaneously. If a new spacer targeting several genomic protospacers is acquired during the first round of primed adaptation in one of two CRISPR array copies, it can initiate primed adaptation at a distant genomic location. New spacers selected during secondary priming can be integrated into the second, as yet unextended CRISPR array (fig. S10D).

Analysis of spacers acquired in *rec* mutants revealed a decrease in secondary priming frequency in the ∆*recJ* mutant (fig. S10E and table S19). This observation is consistent with the overall low level of primed adaptation in this strain (fig. S1, A and B). On the contrary, secondary priming was increased in ∆*recD* and, especially, in ∆*recB* and ∆*recC* cells (fig. S10E and table S19). There are two possible explanations for the secondary priming increase. First, all three mutants have a wider distribution of spacers around the PPS (*34*). This results in a higher percentage of spacers acquired from *rrnE*, *rrnB*, *rrnA*, and *rrnC* located near the PPS, which are capable of promoting secondary priming. Second, ∆*recB* and ∆*recC* cultures are slow growing because of a defect in maintaining the *ter* region (*56*). As a consequence, diploid cells that are unable to undergo cell division are present in the mutant cultures for longer periods of time. The presence of the second chromosome increases secondary priming into yet-unexpanded CRISPR arrays. Inhibition of DNA replication should, by contrast, decrease the frequency of secondary priming into yet-unexpanded CRISPR arrays. This was observed in stationary cultures (fig. S10F and table S20).

### FragSeq reveals products of DNA degradation in the terminus region

Mapping of 31- to 40-nt fragments to the chromosome revealed not only peaks of prespacer-like fragments (Fig. 3, A and B, in purple) but also peaks of unrelated fragments (Fig. 3, A and B, in black). Such fragments were detected at the edges of regions surrounding the PPS and in the terminus region. We will discuss fragments generated in the vicinity of the PPS elsewhere. Fragments generated in the terminus regions are discussed below (figs. S11 to S14).

As can be seen from figs. S11 and S12, there are at least three types of fragments generated in the terminus region. The first type of fragments (marked by red arrows) is detected in *wt* cells in which CRISPR interference was not induced. The peak of these fragments is centered at the *dif* site, spans about 60 kbp, and is composed of fragments ~21 to 220 nt long. The fragments are mapped to both DNA strands in equal quantities. The second type of fragments is detected in ∆*recB*, ∆*recC*, and ∆*recB* ∆*recJ* mutants. Two peaks of such fragments (marked by black brackets) start at both sides of *dif*, span ~200 kbp each, and are composed of fragments ~21 to 120 nt long in ∆*recB* and ∆*recC* strains, although longer fragments are also detected in ∆*recB* ∆*recJ*. Fragments are mapped to both DNA strands in equal quantities. The third type of fragments (marked by blue arrows) is represented by sharp narrow peaks adjacent to the *ter* sequences. Such peaks are composed of fragments of all examined lengths (21 to 520 nt) and are detected in all samples studied.

In all strains, except for the ∆*recB* ∆*recJ* mutant, the highest peak is associated with the *terC* sequence. In ∆*recB* ∆*recJ*, the major peak is at *terA*. Close-up views of the coverage in the vicinity of *ter* sequences (figs. S13 and S14) reveal that (i) the widths of the peaks are ~500 bp, which corresponds to the maximal fragment length (520 nt) examined in this study; (ii) the steeper side of each peak faces the nonpermissive side of the nearest *ter* sequence (the side from which replication stops at *ter*); (iii) fragments from the leading strands approach the nonpermissive *ter* side and either overlap by 1 to 3 nt with the respective 23-nt *ter* sequence or end at ≤7-nt distance from it; (iv) fragments from the lagging strands start ~60-nt from the nonpermissive side of respective *ter* sites, although the *terC*-adjacent peak has only a small ledge at 67-nt distance, while most 5′ ends start 89 nt from *terC*; and (v) for each *ter*, the peak from the leading strand is higher than that from the lagging strand. The potential significance of these results is further addressed in Discussion.

## DISCUSSION

### Prespacer 5′ ends are generated by RecJ or ExoVII exonucleases during primed adaptation

The mechanism(s) generating prespacer 5′ ends in *E. coli* was not known. Our results demonstrate that RecJ is a major contributor to 5′-end trimming. Deletion of *recJ* decreases spacer acquisition and prespacer generation by ~90 and 70%, respectively. Prespacers formed in the absence of RecJ have one additional nucleotide on their 5′ ends, suggesting direct involvement of RecJ in 5′-end processing. This conclusion is supported by in vitro results demonstrating that RecJ trims long single-stranded 5′ ends of dual-forked DNA substrates up to the protospacer sequence protected by Cas1-Cas2.

Our results suggest that ExoVII (the product of *xseAB*) partially substitutes for RecJ activity during prespacer 5′ end trimming. First, ExoVII produces slightly longer ends than RecJ in biochemical experiments, similar to those observed in vivo in ∆*recJ* cells. Second, while primed adaptation efficiency is not affected by the *xseA* deletion, a ~700-fold decrease in adaptation efficiency is detected in ∆*recJ* ∆*xseA* cells, suggesting that ExoVII is responsible for the residual level of adaptation in ∆*recJ* cells. These results are consistent with functional redundancy between ExoVII and RecJ reported in earlier studies of methyl-directed mismatch repair (*57*, *58*) and repair of ultraviolet-induced damages (*59*). Furthermore, ExoVII partially compensates for RecJ activity during recombination and repair of gamma radiation–induced DNA damage in ∆*recD* cells (*60*).

### A dual role of RecBCD in primed adaptation

Our results suggest that although RecBCD participates in generation of prespacer 5′ ends, it cannot produce prespacers alone. In vitro, the RecBCD nuclease was unable to process the ends of the tested prespacer precursors to the length of mature prespacers. Consistently, no changes in the structure of prespacer ends were detected in the nuclease-deficient Δ*recD* mutant. However, when *recB* or *recC* was deleted, prespacers with 1-nt shorter 5′ ends were formed. This suggests direct involvement of the RecBCD helicase activity in prespacer generation. Spacer acquisition was decreased by ~36% in ∆*recD* cells and by 70% in ∆*recB* or ∆*recC* mutants. Prespacer generation in the 1-Mbp PPS region was decreased by ~35% in ∆*recB* or ∆*recC* but was not significantly affected in ∆*recD* cells. When a narrower 0.1-Mbp region was assessed, prespacer generation was decreased by 83% in ∆*recB*, 68% in ∆*recC*, and 49% in ∆*recD* cells. We suggest that RecBCD unwinds double-stranded regions in prespacer precursors and thus provides access to 5′ ends for single strand–specific RecJ. When a Cas1-Cas2–bound prespacer precursor with a terminal double-stranded extension was treated with a combination of RecBCD and RecJ, or RecBC and RecJ, proper 5′ ends were generated. A slight decrease in prespacer generation and spacer acquisition efficiency in the ∆*recD* mutant is likely caused by the fact that the RecBC helicase is slower than RecBCD (*61*, *62*). Since *recBC* mutants are not fully compromised in spacer acquisition, we suggest that the RecBCD helicase activity can be partially substituted by helicases that remain to be identified. One such helicase may be RecQ, since a double *recB recQ* mutant is more strongly affected in spacer acquisition than the corresponding single mutants (fig. S1, C and D). In *wt*, however, RecQ appears to play little, if any, role because the *recQ* single mutant did not significantly differ from *wt*. The reason(s) for extra shortening of prespacers in the absence of RecB or RecC remains unknown. One possible explanation is that the helicase that unwinds prespacer ends in the absence of RecBCD partially displaces the Cas1-Cas2 complex, allowing the removal of additional nucleotides by RecJ/ExoVII.

The PPS region from which protospacers are selected during primed adaptation is wider in ∆*recB*, ∆*recC*, and ∆*recD* cells compared to *wt* (*34*). Here, we show that prespacers are also generated at greater distances from the PPS in ∆*recB*, ∆*recC*, and ∆*recD* cells. We suggest that there are two levels at which RecBCD influences primed adaptation (fig. S15): first, generation of ends of Cas1-Cas2–bound prespacer precursors discussed above (fig. S15A) and, second, interfering with PAC (primed acquisition complex) movement (fig. S15B). RecBCD displaces roadblock proteins immediately after encountering them or after pushing them for up to ~20 kbp (*63*, *64*). The interaction of RecBCD with moving Cas3 or PAC has not been studied to date. The reported velocity of Cas3 and PAC is ~80 to 90 bp/s (*65*), while the reported RecBCD velocity ranges from 300 to 1400 bp/s (*41*, *62*, *64*, *66*). We hypothesize that RecBCD is able to catch up with PAC moving away from the PPS and displace it. Such a mechanism would explain the observed wider area from which prespacers and spacers are selected in the absence of RecBCD. Our data suggest that RecBC is unable to displace PAC, although this requires further investigation.

### DnaQ and ExoT are not essential for generation of prespacer 3′ ends during primed adaptation in vivo

In vitro, DnaQ and ExoT 3′→5′ exonucleases trim 3′ ends of prespacer precursors bound by Cas1-Cas2 to lengths expected for mature prespacers (*26*, *27*). In the present work, we analyzed whether DnaQ and/or ExoT deficiencies affect prespacer generation in vivo and found that these enzymes are not required for proper processing of prespacer 3′ ends; however, when both enzymes are absent, a small but statistically significant decrease in primed adaptation efficiency is observed. Thus, DnaQ and ExoT may contribute to primed adaptation in *wt*; however, their role is clearly nonessential. Since there are at least 13 3′→5′ nucleases in *E. coli* (*67*), it is possible that some of them substitute for DnaQ and ExoT activities. Recently, a similar situation was described for the type II-A CRISPR-Cas system where the Cas9 HNH domain was found to trim long prespacers to the correct size in vitro but was dispensable for adaptation in vivo (*68*).

### A model of primed adaptation in the type I-E CRISPR-Cas system

**Fig. 5.**
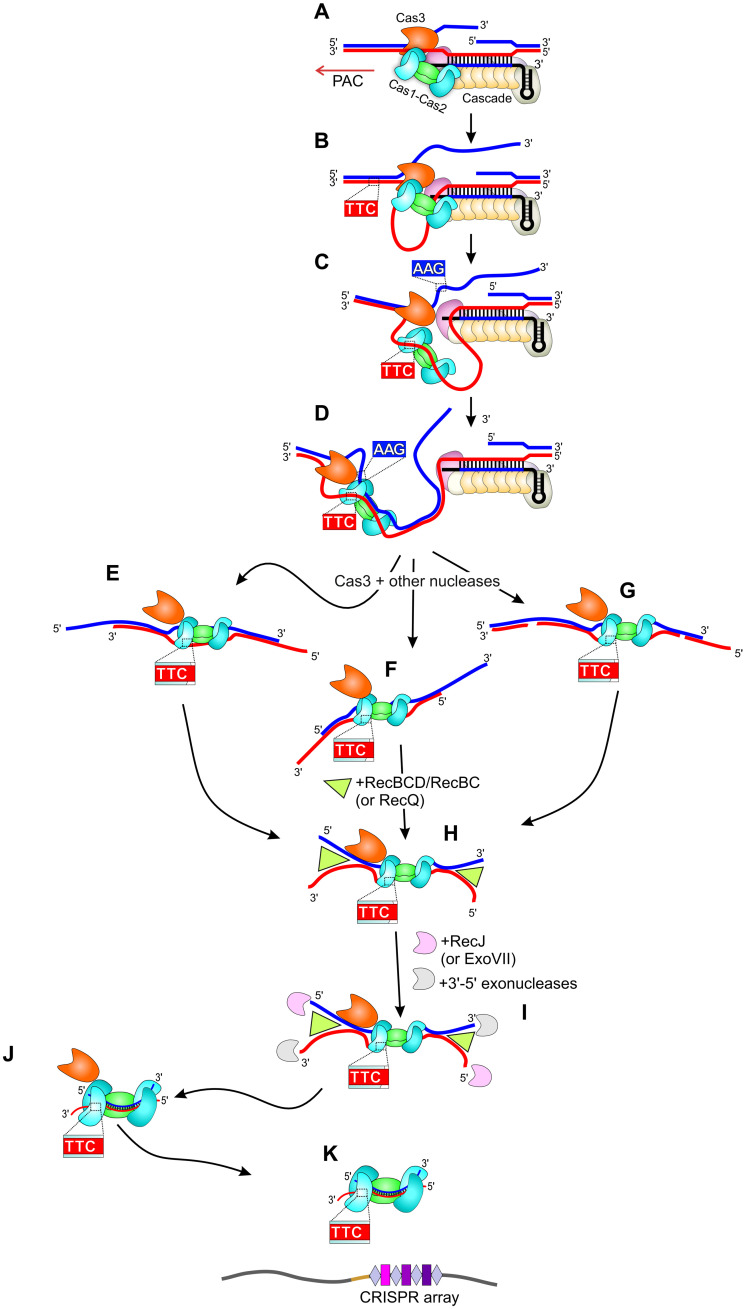
A model of primed adaptation in the type I-E CRISPR-Cas system. (**A**) The PAC composed of Cascade, Cas3, and Cas1-Cas2 is assembled on the PPS (*65*). Cas3 introduces a nick into the nontarget PPS strand (blue). (**B**) The PAC translocates in the PAM-proximal direction (upstream of the PPS) due to the Cas3 3′→5′ helicase activity, separating DNA strands (*65*). (**C**) One of the Cas1 subunits recognizes the 3′-TTC-5′ sequence in the target strand. The recognition leads to the binding of Cas1-Cas2 to the adjacent protospacer (present in a single-stranded form). (**D**) Cas1-Cas2 bound to the single-stranded TTC-associated protospacer anneals it to the complementary AAG-associated strand (*26*). (**E** to **G**) Because of random cleavages by Cas3 or other nucleases, various Cas1-Cas2–bound prespacer precursors are produced. (**H**) The RecBCD or RecBC helicases unwind double-stranded regions flanking the protospacer. In the absence of RecBCD and RecBC, the double-stranded regions are unwound by another helicase (likely RecQ). (**I**) Single-stranded 5′ ends are degraded by the RecJ 5′-3′ exonuclease. ExoVII partially substitutes for RecJ activity. The 3′ ends are trimmed asymmetrically by unidentified nucleases such that a short 3′-NNTT-5′ overhang is left on the PAM-derived end. (**J**) Cas3 is transiently bound to Cas1-Cas2 and the 33/37-nt prespacer (*23*). (**K**) The Cas1-Cas2 complex integrates the prespacer into the CRISPR array.

In Fig. 5, we present a model of prespacer generation during primed adaptation that is consistent with available published data and results obtained in our work. A PAC composed of Cas1-Cas2, Cas3, and Cascade is assembled on the PPS (*65*). The complex moves along DNA because of the Cas3 helicase activity that provides Cas1-Cas2 with single-stranded substrates for binding (*26*). When Cas1-Cas2 recognizes a 3′-TTC-5′ sequence, it binds to the adjacent protospacer sequence, captures its complementary strand generated by the Cas3 helicase-nuclease, and forms duplex DNA. Since during primed adaptation prespacers are selected in a specific orientation relative to the PPS (so that the 3′-TTC-5′ motif is in the T-strand upstream of the PPS and in the NT-strand downstream of the PPS), we speculate that the Cas1-Cas2 positioning within PAC allows sampling of 3′-TTC-5′ in one strand only. Primed adaptation is accompanied by DNA cleavage by Cas3 and, possibly, other cellular nucleases, generating prespacer precursors of various lengths and structures. Cas1-Cas2 bound to the selected protospacer sets the boundaries of a future prespacer by protecting it from degradation. The 5′ ends of double-stranded sequences flanking the protospacer are processed by RecJ in association with RecBCD (or other helicases, e.g., RecQ). ExoVII can perform the 5′-end trimming and partially compensate when RecJ is absent. A set of enzymes generating prespacer 3′ ends in vivo is yet to be determined. We cannot exclude that 3′-5′ exonucleases studied here (DnaQ, ExoT, RecBCD, and ExoVII) participate in this process, but their potential contribution may be masked by functional redundancy. According to our recent data (*23*), Cas3 remains associated with Cas1-Cas2 at least until the mature 33/37-nt prespacer is formed.

### Chromosome replication complexity and CRISPR adaptation

We demonstrate that new spacers selected because of secondary priming can be observed in CRISPR arrays with a single acquired spacer. This observation seemingly contradicts the requirement for an additional spacer that must initiate secondary priming. We suggest that a new spacer acquired into a CRISPR array on one sister chromatid can lead to secondary priming and acquisition of a second new spacer via primed adaptation into the CRISPR array located on the second sister chromatid. In this case, both new spacers are integrated into initially unexpanded CRISPR arrays.

The increase in naïve adaptation efficiency (as measured by the intensity of the agarose gel bands corresponding to CRISPR array amplicons extended by one repeat-spacer unit) in the presence of Cas3 and effector complexes was reported in type I-B (*69*) and I-F (*70*, *71*) CRISPR-Cas systems. Our results suggest that some such new spacers could be acquired because of primed adaptation initiated by naïve spacers acquired from various positions in the genome. Subsequent primed acquisition into the yet unexpanded CRISPR array on a sister chromatid will also occur from any genomic location and therefore may be mistaken for naïve adaption, while in reality it is caused by priming. Depending on chromosome replication complexity (*55*), such priming disguised as naïve adaptation can lead to a substantial increase in adaptation efficiency and affect spacer choices.

### FragSeq confirms two mechanisms of chromosome fragility in the terminus region

A recent study by Mei *et al.* (*72*) revealed two sources of double-strand breaks (DSBs) in the terminus region of *E. coli*. The first source is the binding of the Tus protein to a *ter* sequence that creates a barrier for a replication fork approaching the fork-blocking (nonpermissive) *ter* side (*73*, *74*). If a second fork arrives from the same direction while the first one remains stalled and not resolved, a one-sided DSB forms (*72*, *75*). Such double-stranded termini were observed in *wt* and ∆*recB* cells (*72*). We hypothesize that the narrow peaks of fragments adjacent to nonpermissive *ter* sides revealed in the present study (figs. S11 to S14) originate from replication forks stalled by *ter*-Tus barriers. The fragments we observe could be true fragments generated because of chromosome terminus resection by some cellular nucleases or could be formed by mechanical shearing of stalled replication forks during DNA purification. In either case, the *ter*-proximal side of such fragments should correspond to the ends of stalled replication forks. Our data suggest that the synthesis of the leading strand stops 1 to 7 nt before or up to 3 nt inside the 23-bp *ter* sequence (fig. S14). This result is in good agreement with published in vitro data showing that the primary arrest sites of the leading strand approaching *terB* are at the first two adenines of *terB* or the sixth nucleotide upstream of it (*73*). Previously, the 5′ end of the lagging strand was mapped ~63 to 65 nt upstream of *terB* in vivo (*76*) and 50, 66, or 82 nt upstream of *terB* in vitro (*73*). Our results are consistent with these data, since we detect 5′ ends of lagging strand fragments mainly 59 nt from *terB* (fig. S13). Our results extend the earlier data to other *ter* sites showing that 5′ ends of lagging strand fragments are mainly mapped 61 nt from *terA*, 62 nt from *terD*, and 67 or 89 nt from *terC* (fig. S13).

Overall, the distribution of fragments between four *ter* sites is similar in *wt*, ∆*recB*, ∆*recC*, ∆*recD*, and ∆*recJ* strains with major peaks at *terB*, *terC*, and *terA* (figs. S11 and S12). A notable difference is observed in the ∆*recB* ∆*recJ* mutant, where the *terA*-adjacent peak was predominant. We speculate that, since in most of our samples (except for *wt* uninduced) the genome was cut by Cas3 at the PPS, homologous recombination is required to allow for progression of the replication fork through the PPS toward the nonpermissive side of *terB* and *terC*. Since ∆*recB* and ∆*recJ* mutations inactivate both homologous recombination pathways functional in *E. coli*, RecBCD, and RecFOR (*77*), we detect only fragments derived from the replication fork moving in the opposite direction toward *terA*. Experiments with uninduced ∆*recB* ∆*recJ* cells will be required to test this assumption.

The second source of chromosome breakage in the terminus region characterized by Mei *et al.* (*72*) is failed resolution of catenanes or covalent dimers of sister chromosomes. Both structures are typically resolved at *dif* (*78*–*82*). Two sites enriched with Holliday junctions were found flanking *dif*, suggesting initiation of homologous recombination due to a DSB at *dif* (*72*). The formation of these Holliday junctions was dependent on cell division (*72*). In line with these observations, we detect a peak of fragments spanning *dif* in uninduced *wt* cells (fig. S11). The peak disappears in induced *wt* cells probably because of inhibition of cell division in cells with active CRISPR self-targeting (*24*). We suggest that the fragments detected at *dif* arise because of resection of DNA termini required to form a single-stranded 3′-tail for recombination. These fragments are likely generated by RecBCD.

Extensive degradation around *dif* was observed in the absence of RecBCD, leaving DNA ends located up to 300 kbp away from *dif* (*72*). We detect two symmetrical peaks of fragments located up to ~250 kbp to both sides from *dif* in ∆*recB*, ∆*recC*, and ∆*recB* ∆*recJ* mutants (fig. S11). This implies that some nuclease degrades DNA around *dif* in the absence of RecBCD, leaving DNA termini detected by Mei *et al.* (*72*) and producing fragments detected in our study. Our results indicate that the length of these fragments is ~20 to 300 nt, and they are further shortened by RecJ to ~20 to 120 nt (fig. S11).

Overall, our data confirm the existence of two mechanisms of chromosome breakage in the terminus region (*72*) and demonstrate that FragSeq is a powerful tool that can be used to complement other techniques such as END-seq (*83*) and X-seq (*84*) in comprehensive studies of chromosome fragility.

## MATERIALS AND METHODS

### Strains and plasmids

Bacterial strains used in this study are listed in table S1. Self-targeting strains with deletions Δ*recB*, Δ*recC*, Δ*recD*, Δ*recJ*, and Δ*recB* Δ*recJ* were constructed in previous work (*34*). Self-targeting strains with deletions Δ*xseA*, Δ*recJ* Δ*xseA*, Δ*rnt*, Δ*dnaQ*, Δ*rnt* Δ*dnaQ*, Δ*recQ*, and Δ*recB* Δ*recQ* and BL21-AI strains with deletions Δ*recB* Δ*recJ* and Δ*recJ* Δ*xseA* were constructed in this work using P1 phage transduction by transferring kanamycin resistance cassettes from respective Keio collection strains (*85*). To obtain double deletion mutants, the *kan* resistance gene was removed after the transfer of the first deletion using the pCP20 plasmid (*86*). The pCas1-2 plasmid for the expression of *cas1* and *cas2* genes in prespacer efficiency assay was described earlier (*17*).

### Sample preparation for high-throughput sequencing

Samples for FragSeq and spacer acquisition analysis in self-targeting cells were obtained as described previously (*24*). Cultures were grown in LB at 30°C until the OD_600_ (optical density at 600 nm) reached 0.3 to 0.4. Expression of *cas* genes was induced by 1 mM isopropyl-β-d-thiogalactopyranoside (IPTG) and 1 mM l(+)-arabinose. Cultures were incubated for an additional 5 hours at 30°C, washed in phosphate-buffered saline (PBS), and stored at −70°C. The only exception to this protocol was in the experiment to detect secondary priming (fig. S10F), where self-targeting was induced either in growing cultures (OD_600_ of 0.3 of 0.4) or in stationary-phase cultures (overnight cultures).

For FragSeq, total genomic DNA was isolated by phenol-chloroform extraction, and fragments smaller than ~700 nt were purified using the Select-a-Size DNA Clean & Concentrator kit (Zymo Research) as described previously (*24*). FragSeq libraries were prepared using the Accel-NGS 1S Plus DNA Library Kit (Swift Biosciences) with modifications to the standard protocol recommended by the manufacturer to retain small fragments. The libraries were sequenced on NextSeq 500, HiSeq4000, or MiniSeq Illumina platforms in 2 × 75 or 2 × 150 bp paired-end modes. The sequencing coverage needed was determined on the basis of the results of library size evaluation such that at least a few thousand 31- to 40-nt reads were expected. Library size evaluation was performed using the Bioanalyzer 2100 system.

For analysis of spacer acquisition efficiency during self-targeting, cell lysates were prepared by concentrating cells 20× in Milli-Q water and heating at 95°C for 5 min. Cell debris was removed from lysates by centrifugation at 16*g* for 1 min. Amplified mixtures of extended and nonextended CRISPR arrays were purified using the GeneJET PCR Purification Kit. Primers used for amplification of CRISPR arrays are listed in table S21. Sequencing libraries were prepared using NEBNext Ultra II Library Prep Kit (New England Biolabs) and sequenced on the MiniSeq, HiSeq4000, or NovaSeq 6000 Illumina platforms in 2 × 150 bp paired-end read mode. The sequencing coverage was planned based on visual assessment of spacer adaptation efficiency using agarose gel electrophoresis so that at least a thousand reads corresponding to expanded CRISPR arrays were expected in the sequencing data.

Prespacer efficiency assay was performed as described earlier (*24*, *35*) except for the polymerase chain reaction (PCR) stage that was performed with primers containing 5′-terminal extensions corresponding to NEBNext Illumina adapter sequences (table S21). The libraries were purified, indexed, and sequenced on the MiniSeq or HiSeq4000 Illumina platforms in 2 × 150 bp paired-end read mode as described above. Oligonucleotides used for electroporation are listed in table S8. The sequencing coverage was planned based on visual assessment of spacer adaptation efficiency using agarose gel electrophoresis so that at least a thousand reads corresponding to expanded CRISPR arrays were expected in the sequencing data.

### High-throughput sequencing data analysis

Bioinformatic analysis was performed in RStudio using ShortRead and Biostrings packages (*87*). Reads containing more than 10% of positions with Phred quality score <20 were discarded, and adapter sequences were trimmed from read 3′ ends. Plots were generated using ggplot2 (*88*). Sequence logos were generated using the ggseqlogo package (*89*).

For FragSeq analysis, reads were mapped to the KD403 reference genome using a custom-written R script. During the first stage of library preparation following the protocol of the Accel-NGS 1S Plus DNA Library Kit (Swift Biosciences), a low complexity tail with an average length of 8 bases (and up to ~12 bases) mostly composed of C and T nucleotides is ligated to 3′ ends of fragments. To account for these tails, the last 15 nt were removed from 3′ ends of forward reads followed by forward read mapping with several mismatches allowed (up to 5% of read length). The 5′ ends of mapped forward reads correspond to the fragments’ 5 ends. The first 20 nt of reverse reads, which include the tails and the 3′-end nucleotides of fragments, were mapped without mismatches allowed. If a read was not aligned or was not properly oriented relatively to the corresponding forward read, 1 nt of a presumptive tail was removed from the 5′ end of the reverse read and the first 20 nt of the trimmed reverse read were mapped again. The cycles of reverse read alignment and trimming by 1 nt were repeated until the appropriate hit was found but not more than 15 times. The positions of 5′ ends of mapped reverse reads were regarded as positions of the fragments’ 3 ends.

During analysis of CRISPR amplicons derived from self-targeting cells, forward and reverse reads overlapping by at least 20 nt were merged. The CRISPR repeat sequence was searched with two mismatches allowed. For estimation of primed adaptation efficiency, the number of merged reads with 1, 2, 3, or 4 CRISPR repeats corresponding to arrays with 0, 1, 2, or 3 newly acquired spacers was calculated. Primed adaptation efficiency was calculated as the total number of newly acquired spacers divided by the total number of CRISPR arrays.

During analysis of CRISPR amplicons derived from BL21-AI, BL21-AI ∆*recB* ∆*recJ*, and BL21-AI ∆*recJ* ∆*xseA* cells in prespacer efficiency assays, forward and reverse reads overlapping by at least 20 nt were merged, and CRISPR repeat sequences were searched with two mismatches allowed. Newly acquired spacer sequences corresponding to the top spacer strand were extracted from CRISPR arrays containing at least two repeats. Newly acquired spacers were mapped with three mismatches allowed to the Bl21-AI genome, the pCas1-2 plasmid, and the sequences of oligonucleotides used for electroporation. The total number of CRISPR arrays, the total number of oligo-derived spacers, and the number of properly processed oligo-derived spacers were calculated. We define properly processed oligo-derived spacers as 33-bp oligo-derived spacers that start with the PAM-derived G. Oligo acquisition efficiency was calculated as the number of properly processed oligo-derived spacers per CRISPR array (%) further adjusted to remove the batch effect caused by replicating experiments on different days. In all cases, the batch-group design was balanced, i.e., the study groups were equally represented in all batches (tables S9 to S11). Therefore, we removed the batch effect by subtracting the mean in each batch from all measurements in that batch resulting in zero-centered values (*90*).

Statistical analysis was performed in RStudio. The methods used are specified in the figure legends. The Benjamini-Hochberg adjustment was applied to *P* values when hypotheses with multiple comparisons were tested.

### Protein purification

Cas1 and Cas2 were purified as described previously (*27*). RecBCD and RecBC were purified as described previously (*62*).

### Exonuclease footprinting

For exonuclease footprinting, unlabeled and 3′-fluorescein–labeled DNA oligonucleotides purchased from IDT (table S12) were used to assemble prespacer precursor substrates. Exonuclease RecJ and ExoVII reactions were carried out on double-forked DNA substrates containing a central 23-bp duplex flanked with 5′ and 3′ overhangs (Fig. 4A). For ExoV (RecBCD) nuclease footprinting, a double-forked DNA substrate was supplemented with a complementary (to 5′ overhang) strand to provide a blunt end configuration of the substrate (Fig. 4B and fig. S7). Exonuclease reactions were performed with 0.2 μM DNA substrate bound by the 1.6 μM Cas1-Cas2 complex, in 10 μl of binding buffer [40 mM tris-HCl (pH 7.5), 50 mM NaCl, 10 mM MgCl_2_, 0.5 mM tris(2-Carboxyethyl)phosphine (TCEP), and bovine serum albumin (BSA; 0.1 mg/ml)] supplemented with 1 mM adenosine triphosphate (ATP) when RecBCD was used. Before these reactions, the Cas1-Cas2 complex was reconstituted in the binding buffer (without magnesium) by incubating Cas1 and Cas2 subunits at the molar ratio of 2:1 on ice for at least 20 min. This mixture was diluted 10-fold into the binding reaction with DNA substrates and incubated at room temperature for 20 min. Exonuclease reactions were initiated by addition of 30 U of RecJ or 10 U of ExoVII (both from NEB), or RecBC or RecBCD (final concentration of 50 nM) to 10 μl of binding reaction and incubated at 37°C for 30 min. The reactions were terminated by addition of 15 μl of formamide loading buffer containing heparin (0.4 mg/ml) and heated in a boiling water bath for 1 min. The digested DNA fragments were resolved by urea-denaturing 8% polyacrylamide gel electrophoresis (PAGE). The gel was screened and imaged with an Amersham Typhoon scanner.

### Electrophoretic mobility shift assay

DNA duplexes were formed by mixing 5′-[^32^P]–labeled strands and unlabeled strands (table S18) at a molar ratio of 1:2 in Nuclease-Free Duplex Buffer (IDT), heating for 2 min at 95°C, and slowly cooling to 20°C. The Cas1-Cas2 complex was reconstituted as described in the previous section. The binding reaction was carried out in 10 μl of binding buffer [40 mM tris-HCl (pH 7.5), 50 mM NaCl, 10 mM MgCl_2_, 0.5 mM TCEP, and BSA (0.1 mg/ml)] containing 25 nM DNA substrate and 2 μM Cas1-Cas2 complex. Binding reactions were incubated for 30 min at 37°C, loaded onto a native precast 4 to 20% gradient polyacrylamide gel (Novex, Invitrogen), and run in tris-glycine buffer at 20 mA for 2 hours. The bound complexes were visualized with Typhoon phosphorImager and calculated by ImageQuant software (GE Healthcare).

### Permanganate probing

KMnO_4_ probing was performed with 10 nM 5′-[^32^P]–labeled DNA substrate (table S18) and 800 nM Cas1-Cas2 complex in 10 μl of binding buffer [40 mM tris-HCl (pH 7.5), 50 NaCl, 10 mM MgCl_2_, 0.5 mM TCEP, and BSA (0.1 mg/ml)]. The binding reaction was incubated at 37°C for 30 min, and the probing reaction was initiated by adding KMnO_4_ to a final concentration of 2.5 mM. Reactions were incubated for 10 s and quenched by the addition of 10 μl of 1% 2-mercaptoethanol. The reactions were extracted with phenol-chloroform mixture, followed by ethanol precipitation. DNA pellets were dissolved in 50 μl of freshly prepared 1 M piperidine and heated at 95°C for 10 min. Piperidine was removed by chloroform extraction, and DNA was ethanol-precipitated. Pellets were dissolved in 10 to 15 μl of formamide loading buffer, and products were separated by denaturing 11% PAGE and visualized with Typhoon phosphorImager (GE Healthcare).

## Notes

View/request a protocol for this paper from *Bio-protocol*.
