# Supplementary material for: Host nucleases generate prespacers for primed adaptation in the E. coli type I-E CRISPR-Cas system
Source: Sci Adv. 2022 Nov 25;8(47):eabn8650. doi: 10.1126/sciadv.abn8650 (PMC9699676; doi:10.1126/sciadv.abn8650)
Supplement: Supplementary file 1 — Supplementary Text Figs. S1 to S15 Tables S1 to S22 [file sciadv.abn8650_sm.pdf]

Science Advances
AAAS

Supplementary Materials for

Host nucleases generate prespacers for primed adaptation in the *E. coli* type I-E CRISPR-Cas system

Anna A. Shiriaeva *et al.*

Corresponding author: Konstantin Severinov, severik@waksman.rutgers.edu;
Anna A. Shiriaeva, annabiologic@gmail.com

Sci. Adv. **8**, eabn8650 (2022)
DOI: 10.1126/sciadv.abn8650

This PDF file includes:

Supplementary Text
Figs. S1 to S15
Tables S1 to S22

Supplementary Text

The previous protocol for FragSeq library preparation consisted of sequential ligation of single-stranded adapters to fragments' ends and indexing PCR; each round was followed by DNA purification from PAGE and precipitation with sodium acetate, glycogen, and ethanol *(24)*. An ~1.4-fold prevalence of 31-40-nt fragments mapped to the NT-strand upstream and to the T-strand downstream of the PPS was observed when the original protocol for library preparation was used *(24)*.

In the present paper, we switched to a faster protocol for library preparation using the Accel-NGS 1S Plus DNA Library Kit (Swift Biosciences). In this protocol, at each step, DNA is purified on magnetic beads. As a result, shorter fragments may be recovered to a lesser extent than longer ones, which may create a bias in the number of reads of different sizes. To test this conjecture, we added 10 double-stranded oligonucleotides mixed in equimolar ratio to 12 samples of genomic DNA used in the experiment presented in Fig. S8. DNA fragments (including the added oligos) were purified and processed via our standard pipeline (Select-a-Size Kit by Zymo Research, libraries preparation using the Accel-NGS 1S Plus DNA library kit by Swift Biosciences, and Illumina sequencing). The sequences of added oligonucleotides were designed such that they were easily distinguished from each other and from the *E. coli* genomic DNA after sequencing (Table S22). The lengths of the added oligonucleotides ranged from 31 to 40 bp with a 1-bp increment. The percentage of reads mapped to each of the ten oligonucleotides is shown in Fig. S2A. As can be seen, the number of recovered oligos does, indeed, decrease with shortening of the oligo length. We fitted a regression line to these data and, based on the regression line, estimated the loss coefficients for each oligo length. When the number of reads mapped to each oligonucleotide is multiplied by the corresponding loss coefficient, the equimolar ratio gets nearly restored.

When the amounts of 31-40-nt fragments was analyzed in a sample prepared using the Accel-NGS 1S Plus DNA Library Kit, a 2.13-fold prevalence of fragments mapped to the T-strand upstream of the PPS and a 2.43-fold prevalence of fragments mapped to the NT-strand downstream of the PPS was observed (Fig. S2B, top; Fig. S2D, left). Using the loss coefficients calculated for the oligos (Fig. S2A), we adjusted the amounts of genomic fragments. As a result, the ratio of fragments decreased from 2.13 to 0.99 upstream of the PPS (T/NT ratio) and from 2.43 to 1.18 (NT/T ratio) downstream of it (Fig. S2B, bottom; Fig. S2D, right). Therefore, the initial imbalance in the amounts of complementary fragments was caused, at least in part, by the loss of shorter fragments during library preparation.

To minimize the effect of short fragment loss, we adjusted the results of all experiments shown in the manuscript using the loss coefficients (Fig. 2A, C; Fig. S8A). Slight imbalance was still observed in some samples (1.66 at maximum) even after the adjustment (Fig. 2A). It is yet to be determined if this is the artifact of a particular library preparation or a specific characteristic of the strain.

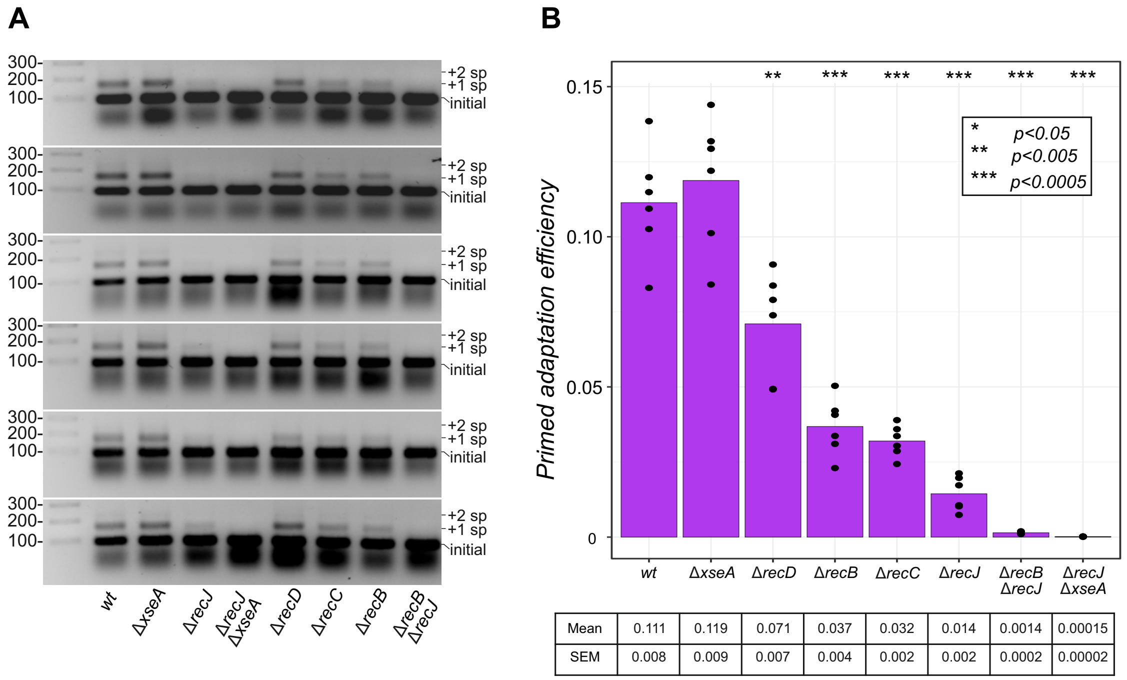

Mean	0.111	0.119	0.071	0.037	0.032	0.014	0.0014	0.00015
SEM	0.008	0.009	0.007	0.004	0.002	0.002	0.0002	0.00002

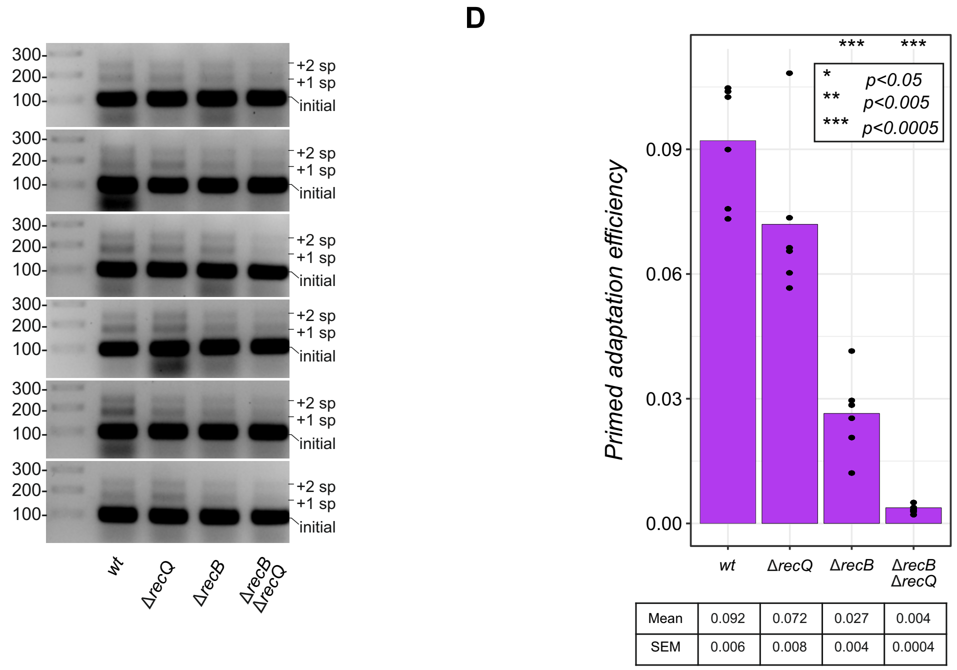

Mean	0.092	0.072	0.027	0.004
SEM	0.006	0.008	0.004	0.0004

Fig. S1. Primed adaptation efficiency is decreased in Δ*recD*, Δ*recB*, Δ*recC*, Δ*recJ*, Δ*recB* Δ*recJ*, Δ*recJ* Δ*xseA*, and Δ*recB* Δ*recQ* mutants. (A, B) Efficiency of primed adaptation in the *wt* and Δ*recD*, Δ*recB*, Δ*recC*, Δ*recJ*, Δ*xseA*, Δ*recB* Δ*recJ*, Δ*recJ* Δ*xseA* strains. (C, D) Efficiency of primed adaptation in the *wt*, Δ*recB*, Δ*recQ*, and Δ*recB* Δ*recQ* strains. In panels A and C, amplification products of unexpanded CRISPR arrays ("initial") and CRISPR arrays with one ("+1 sp") or two ("+2 sp") additional spacer-repeat units resolved by electrophoresis in agarose gel are shown. A molecular-weight size marker is shown on the left. Results of 6 independent biological replicates are presented. In panels B and D, primed adaptation efficiency is calculated as the ratio of newly acquired spacers to the total number of CRISPR arrays determined by high-throughput sequencing of CRISPR array amplicons. Asterisks indicate statistically significant differences between a mutant and the *wt* ($p<0.05$) in pairwise Welch's t-test. Points represent individual values obtained in 6 biological replicates. For each strain, the mean and standard error of the mean are shown in the table under the bar graph.

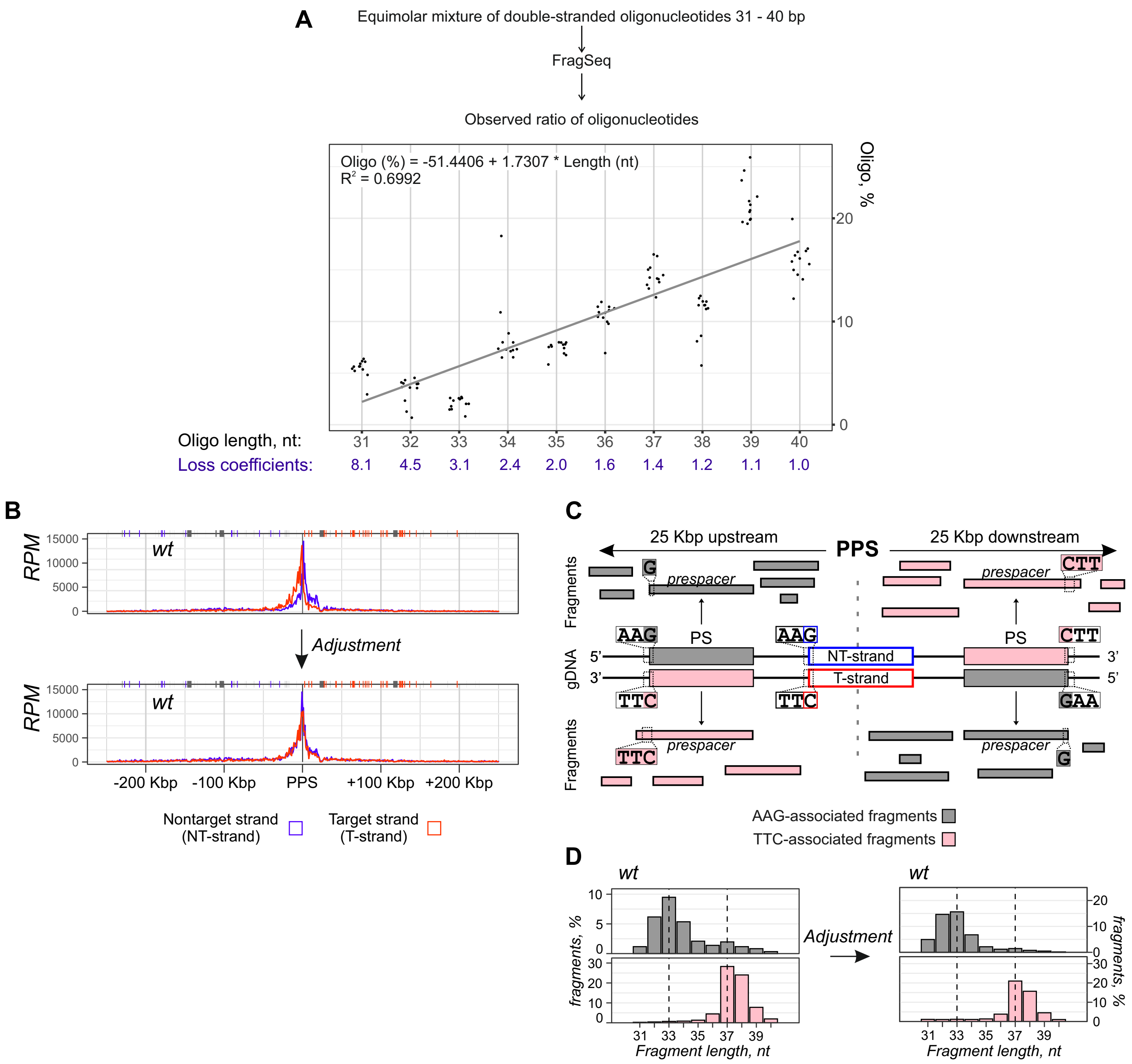

Fig. S2. The imbalance between the number of fragments originating from NT- and T-strands up- and downstream of the PPS is caused by a library preparation bias. (A) Recovery of double-stranded oligos of different lengths by the FragSeq procedure used in this work. For each length, one model double-stranded oligonucleotide was used. The oligonucleotides of different lengths had different sequences and thus could be easily distinguished from one another after sequencing. Twelve biological replicates (shown by dots) were performed. The regression line fitting the data is presented. Below, calculated loss coefficients for each tested oligo lengths are shown. (B) Normalized sequence coverage (reads per million, RPM, in 1-Kbp bins) by 31-40-nt ssDNA fragments of a region around the PPS (± 250 Kbp). At the top, the plot obtained for the *wt* strain without the adjustment for recovery of fragments of different lengths is shown. The plot below is drawn using adjusted fragment abundancies obtained by multiplying the initial values by fragment-length specific loss coefficients from panel A. As can be seen, the bias observed in the top plot disappears as a result of the adjustment. For each plot, the locations of the Chi sites are indicated by red and blue vertical lines for Chi located in T- and NT-strands, respectively. Gray boxes indicate regions with repetitive sequences where unambiguous fragment mapping is impossible.

Reads matching such repetitive sequences were excluded from analysis, which leads to an apparent decrease in fragment coverage. (C) A 50-Kbp region around the PPS is schematically presented as in Fig. 2B. Oppositely oriented protospacers (PS) that are predominantly selected as spacers during primed adaptation are depicted to the left (upstream) and to the right (downstream) of the PPS. Fragments mapping to the NT-strand upstream of the PPS and to the T-strand downstream of the PPS are shown in gray. In the wild-type, AAG-associated fragments are found in this group. Fragments mapping to the T-strand upstream of the PPS and to the NT-strand downstream of it are shown in pink. In the wild-type, TTC-associated fragments are found in this group. (D) Length distributions of 31-40-nt fragments originating from the 50-Kbp region around the PPS. 100% corresponds to all 31-40-nt fragments from both strands in this region. The plot at the left shows the number of obtained fragments without any adjustment. The plot at the right has been adjusted using fragment-length specific loss coefficients from panel A.

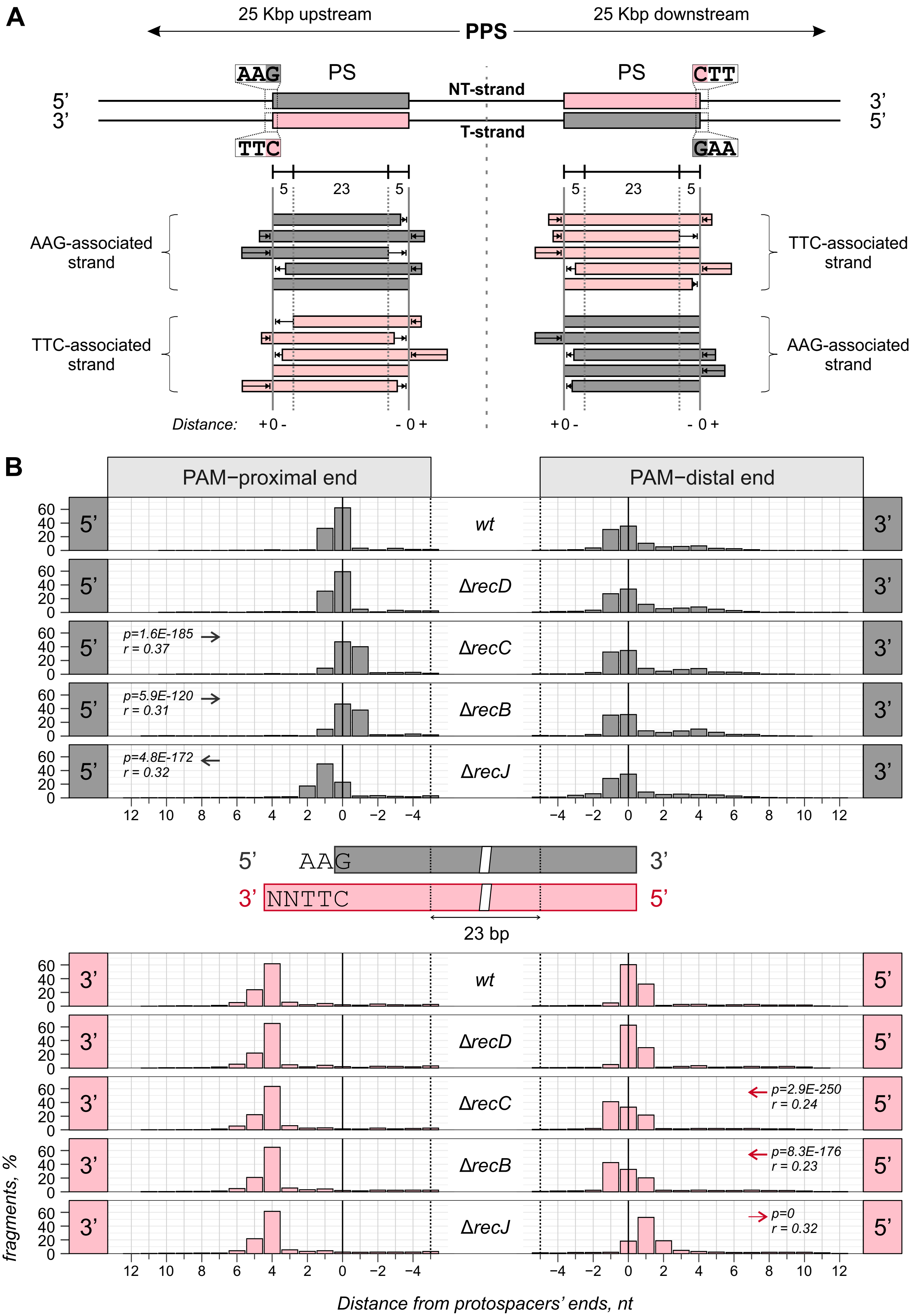

A
25 Kbp upstream
PPS
25 Kbp downstream
AAG
PS
NT-strand
T-strand
TTC
CTT
GAA
5’
3’
5
23
5
AAG-associated strand
TTC-associated strand
Distance:
+0 -
- 0 +
B
PAM−proximal end
PAM−distal end
wt
ΔrecD
ΔrecC
ΔrecB
ΔrecJ
p=1.6E-185
r = 0.37
p=5.9E-120
r = 0.31
p=4.8E-172
r = 0.32
AAG
NNTTC
23 bp
p=2.9E-250
r = 0.24
p=8.3E-176
r = 0.23
p=0
r = 0.32
fragments, %
Distance from protospacers’ ends, nt

Fig. S3. Processing of prespacer ends varies in different *E. coli* strains. (A) A strategy for selection of potential prespacer fragments. Coordinates of all 865 possible 33-bp protospacers with an adjacent 5'-AAG-3'/3'-TTC-5' PAM were determined in a 50-Kbp region centered at the PPS. Upstream of the PPS, only protospacers with the 5'-AAG-3' motif in the top (nontarget) strand were selected. Downstream of the PPS, only the protospacers with the 5'-AAG-3' motif in the bottom (target) strand were selected. For each protospacer, coordinates of its central 23-bp region were determined. 31-40-nt fragments that contained the central 23-bp protospacer parts were selected for further analysis. All fragments that mapped to the protospacer strand associated with the 5'-AAG-3' motif were pooled (shown in gray). Fragments mapped to the protospacer strand associated with the 3'-TTC-5' motif were also pooled (shown in pink). For each fragment, distances from its ends to corresponding protospacer ends were calculated. Zero values were assigned to the distances if fragments' ends coincided with the protospacers' ends. Positive values were assigned to the distances if fragments' ends lay outside protospacers. Negative values were assigned to the distances if fragments' ends lay within protospacers. (B) Distribution of the distances from fragments' ends to the ends of protospacers in indicated strains. Values within individual plots are p-values of one-sided Mann Whitney U tests computed in comparisons with the *wt* and effect size values r. Only p-values less than 0.05 and r-values greater than 0.1 are shown. The arrows indicate the direction of the shift along the X-axis in samples compared with the *wt*.

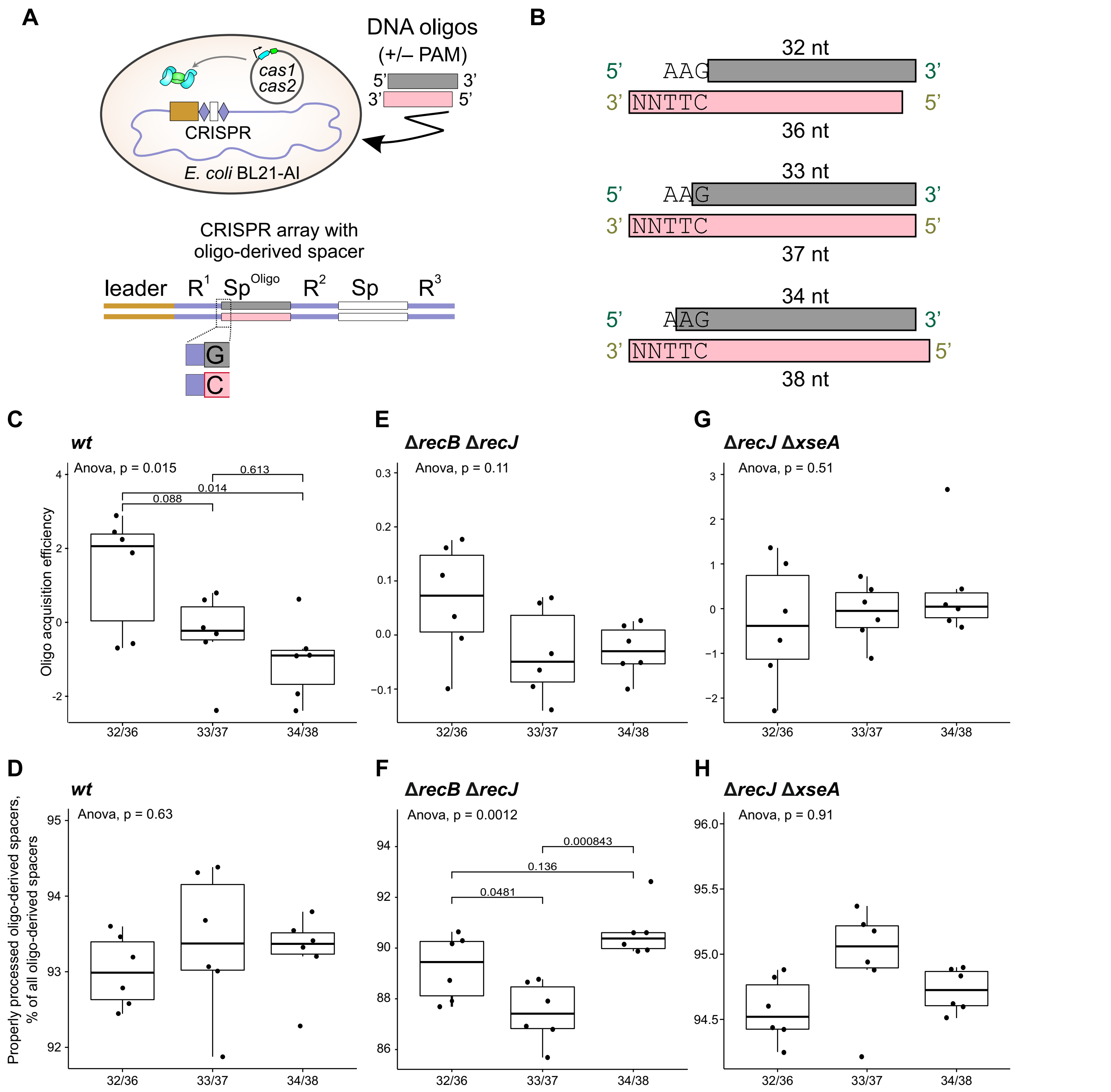

Fig. S4. Shortening or extending prespacer 5' ends by 1 nt does not affect prespacer acquisition efficiency. (A) Prespacer acquisition efficiency assay. Top, electroporation of a synthetic DNA oligo into cells containing a CRISPR array and a plasmid that directs expression of *cas1* and *cas2*. Bottom, oligo-derived spacers integrated into the CRISPR array. (B) Oligonucleotides used in prespacer acquisition efficiency assay. The results of oligo electroporation into the *wt* (C, D), Δ*recB* Δ*recJ* (E, F), and Δ*recJ* Δ*xseA* (G, H) strains are shown. (C, E, G) Zero-centered oligo acquisition efficiencies after removal of batch effects by subtracting the mean of the measurements for each batch. The measurements were taken 2 hours after electroporation. (D, F, H) Percent of properly processed oligo-derived spacers among all oligo-derived spacers. Properly processed oligo-derived spacers are defined as 33-bp spacers starting with the PAM-derived G/C pair. p-values of one-way ANOVA and Tukey HSD tests are reported.

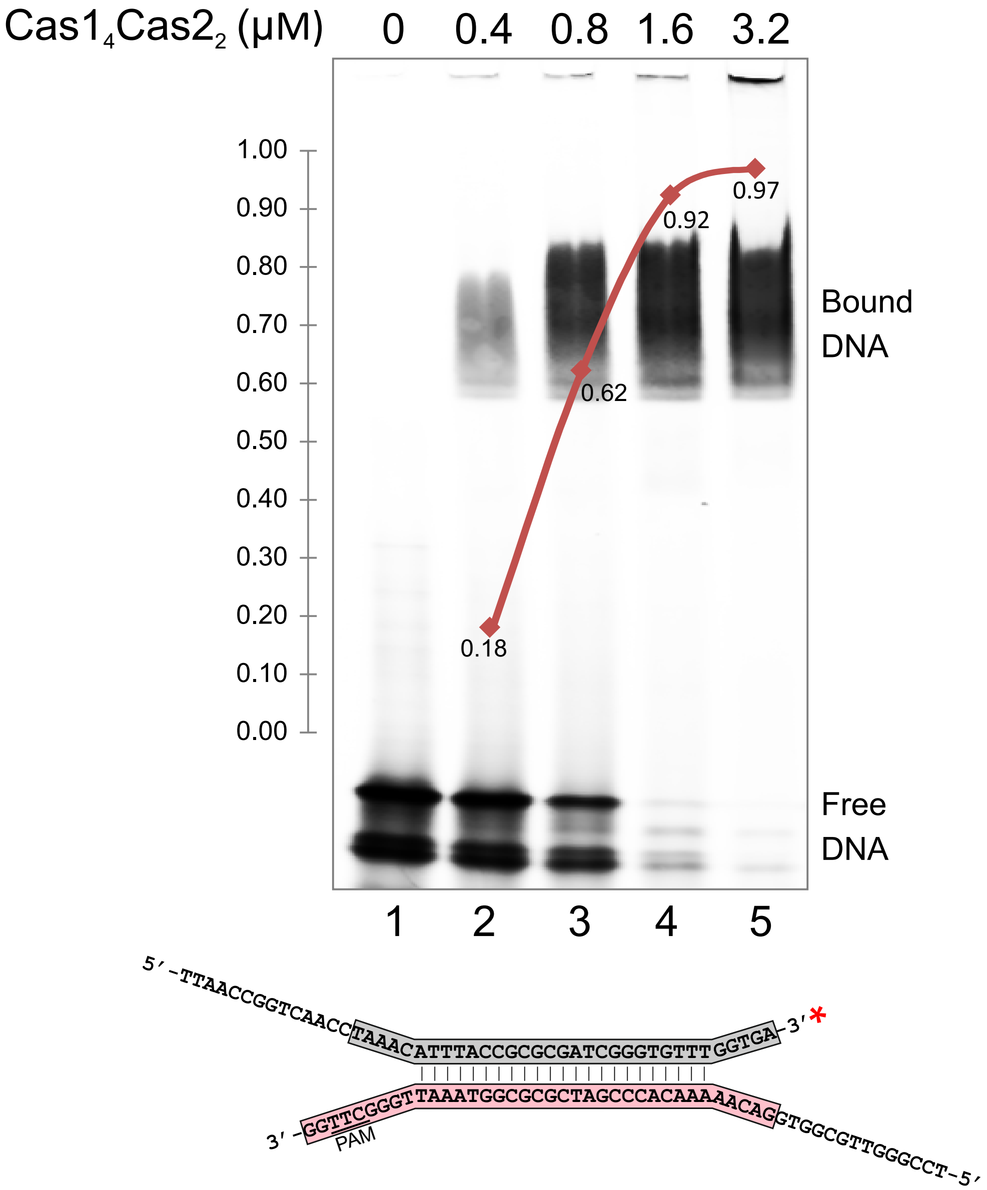

Fig. S5. Cas1-Cas2 binding to a prespacer precursor substrate. (A) Serial dilutions of Cas1-Cas2 were incubated with 0.2 µM DNA substrate (shown at the bottom) in the binding buffer. Formation of the Cas1-Cas2-DNA complex was evaluated by EMSA using native 4-20% PAGE in Tris-Glycine buffer. Fractions of the Cas1-Cas2-DNA complex were plotted against Cas1-Cas2 concentrations (red line). The Cas1-Cas2 concentration, at which ~92% of DNA substrate was shifted (lane 4), has been chosen as working concentration in exonuclease footprinting experiments. The apparent Kd is ~0.5 µM. * is 3'-end fluorescein.

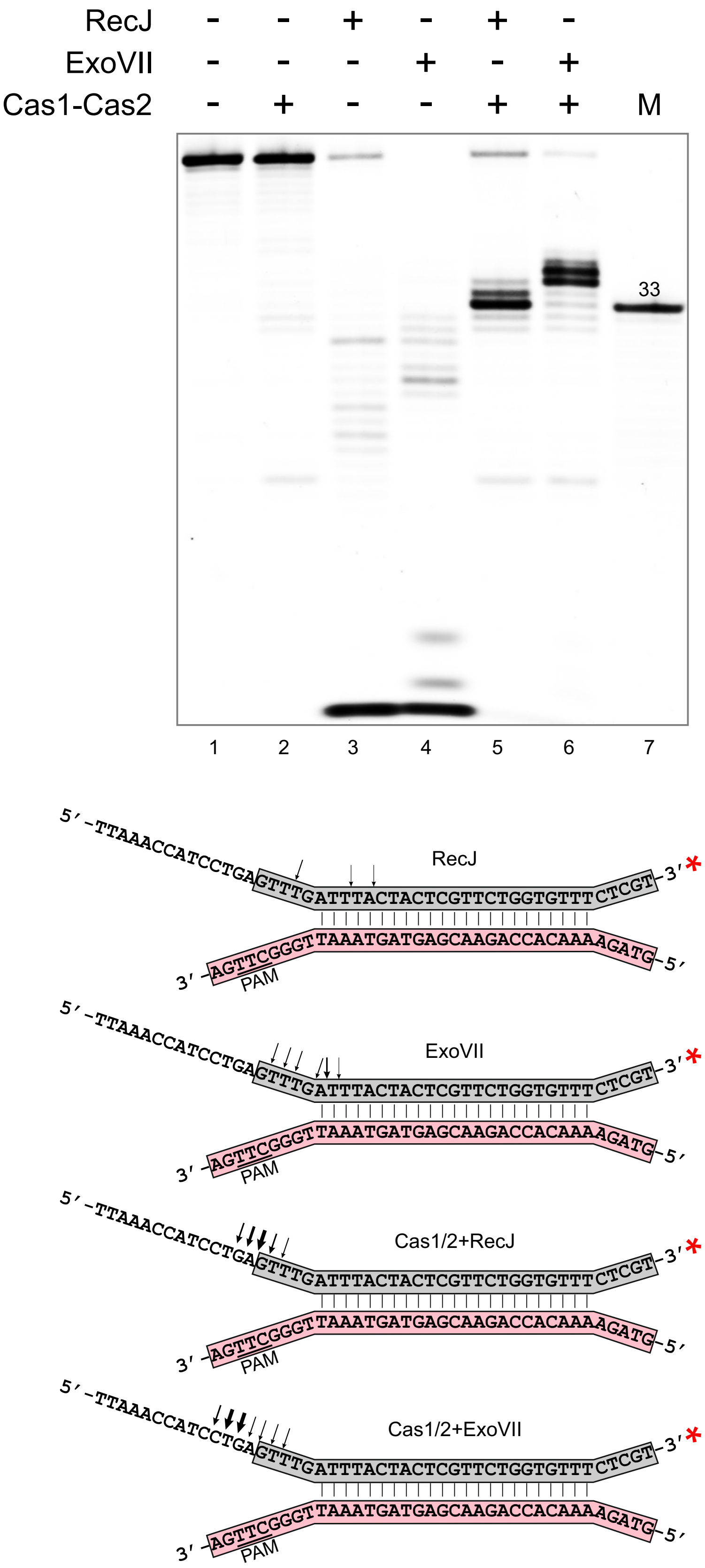

Fig. S6. Digestion of prespacer 5' ends by RecJ and ExoVII. A double-forked DNA substrate composed of a 23-bp central duplex with single-stranded extensions (shown at the bottom) was labeled at one of the 3' ends with fluorescein (shown by asterisks) and treated with RecJ or ExoVII exonucleases in the presence/absence of Cas1-Cas2. A fluorescein-labeled 33-nt oligonucleotide (highlighted in gray on schemes at the bottom) was used as a marker. The positions of cleavage sites observed in the presence of different proteins are shown by arrows. Note that rare cleavages in the absence of Cas1-Cas2 are within the duplex part of the substrate.

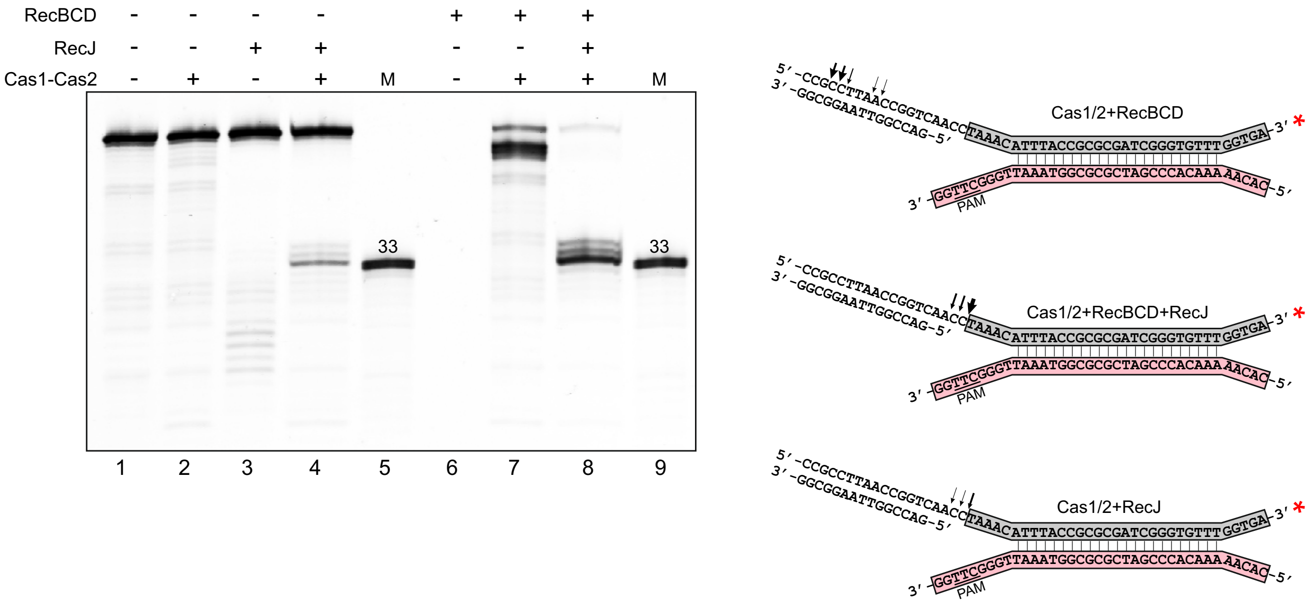

Fig. S7. Processing of prespacer 5' ends by RecJ and RecBCD nucleases. (A) A double-forked DNA substrate (shown on the right) was labeled with fluorescein and used to study RecBCD-assisted processing of double-stranded terminal regions by RecJ. Reaction products were resolved by denaturing polyacrylamide gel electrophoresis. A fluorescein-labeled 33-nt oligonucleotide (highlighted in gray in schemes on the right) was used to map the PAM-proximal boundary of the DNA substrate protection by Cas1-Cas2. Arrows show the positions of cleavage sites observed in the presence of different proteins.

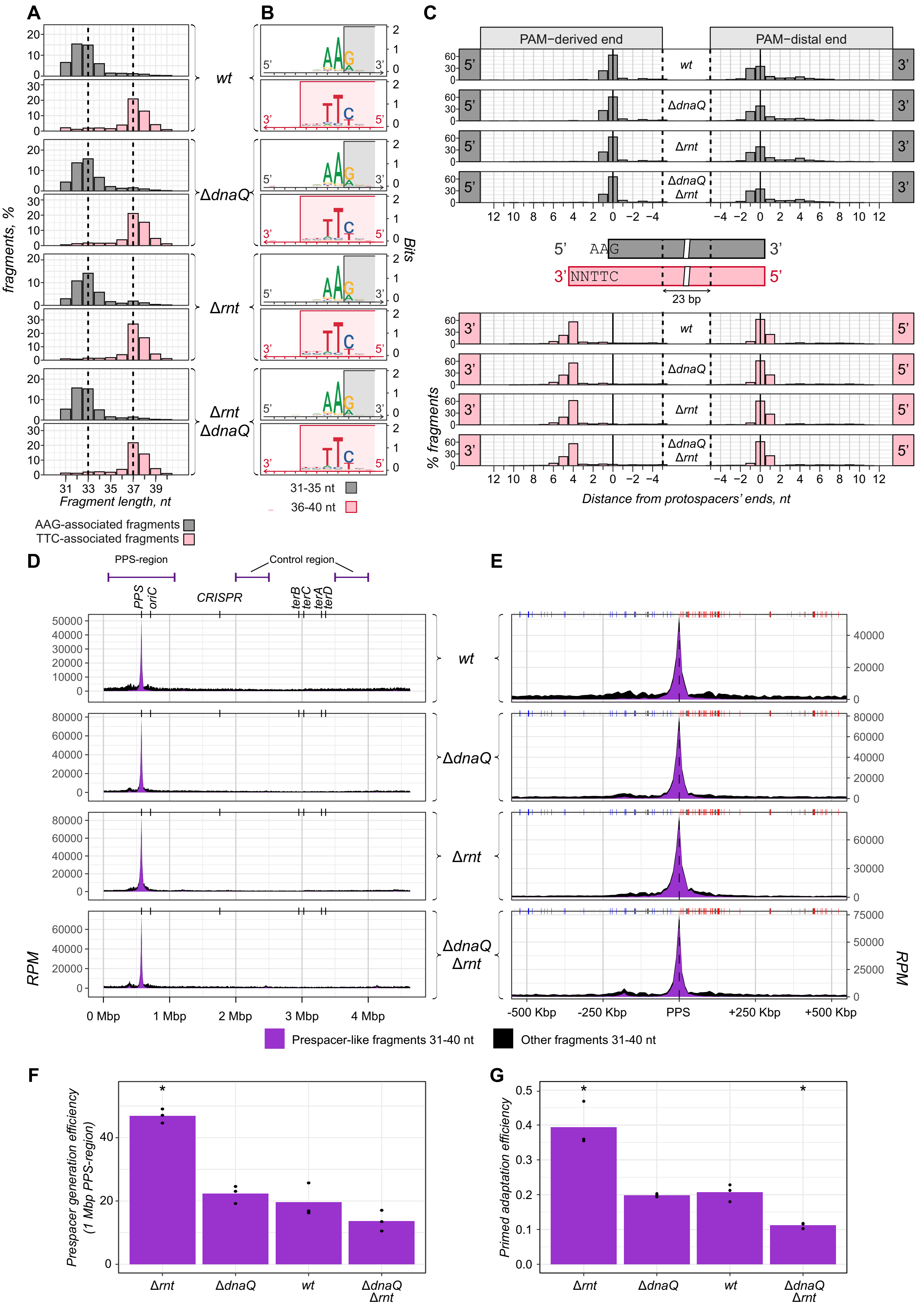
A
B
C
fragments, %
wt
ΔdnaQ
Δrnt
Δrnt ΔdnaQ
Fragment length, nt
AAG-associated fragments
TTC-associated fragments
Bits
31-35 nt
36-40 nt
PAM−derived end
PAM−distal end
23 bp
5' AAG 3'
3' NNTTC 5'
% fragments
Distance from protospacers' ends, nt
D
E
PPS-region
Control region
PPS
oriC
CRISPR
terB
terC
terA
terD
RPM
0 Mbp
1 Mbp
2 Mbp
3 Mbp
4 Mbp
-500 Kbp
-250 Kbp
PPS
+250 Kbp
+500 Kbp
Prespacer-like fragments 31-40 nt
Other fragments 31-40 nt
F
G
Prespacer generation efficiency (1 Mbp PPS-region)
Primed adaptation efficiency
Δrnt
ΔdnaQ
wt
ΔdnaQ Δrnt

Fig. S8. DnaQ and ExoT are not essential for prespacer generation during primed adaptation. (A) Length distributions of 31-40-nt fragments originating from the 50-Kbp PPS-region after adjustment using fragment-length specific loss coefficients from Fig. S2A. A 100% value corresponds to all 31-40-nt fragments from both strands in this region. (B) Sequence alignments of fragments' ends and adjacent genomic regions. Gray rectangles correspond to 5'-terminal sequences of 31-35-nt AAG-associated fragments. Pink rectangles correspond to 3'-terminal sequences of 36-40-nt TTC-associated fragments. (C) Processing of prespacer ends in Δ*dnaQ*, Δ*rnt*, and Δ*dnaQ* Δ*rnt* strains (*rnt* encodes ExoT). Distribution of the distances from fragments' ends to the ends of corresponding protospacers (see the strategy for selecting fragments for analysis in Fig. S3A legend). (D) Distribution of 31-40-nt fragments along the *E. coli* chromosome. Prespacer-like fragments (purple) are defined in Fig. 3. All other 31-40-nt fragments are shown in black above prespacer-like fragments. Normalized sequence coverage (RPM, in 10-Kbp bins) is shown. Coordinates on the X-axes represent positions on the chromosome. *oriC* - replication origin; *terA*, *terB*, *terC*, *terD* - sites of replication termination; "CRISPR" denotes the position of the CRISPR array. "PPS-region" and "control region" indicate regions used to calculate prespacer generation efficiency near the PPS shown in F. (E) A close-up of the sequence coverage from D for a 1-Mbp region centered at the PPS (coordinates on the X-axes represent a distance from the PPS). Chi sites properly oriented relative to the PPS are shown above each coverage plot as blue or red vertical lines. Gray boxes indicate regions of repetitive sequences. Reads matching such repetitive sequences were excluded from analysis, which leads to an apparent decrease in fragment coverage. (F) Prespacer generation efficiency calculated as the ratio of prespacer-like fragments from the PPS-region to prespacer-like fragments from the control region. The coordinates of PPS- and control regions are shown in panel D. (G) Primed adaptation efficiency calculated as the ratio of newly acquired spacers to the total number of CRISPR arrays determined by high-throughput sequencing of CRISPR array amplicons. In F and G, * indicates statistically significant differences between a mutant and the *wt* ($p<0.05$) in pairwise T-tests. Individual points show values obtained in 3 biological replicates.

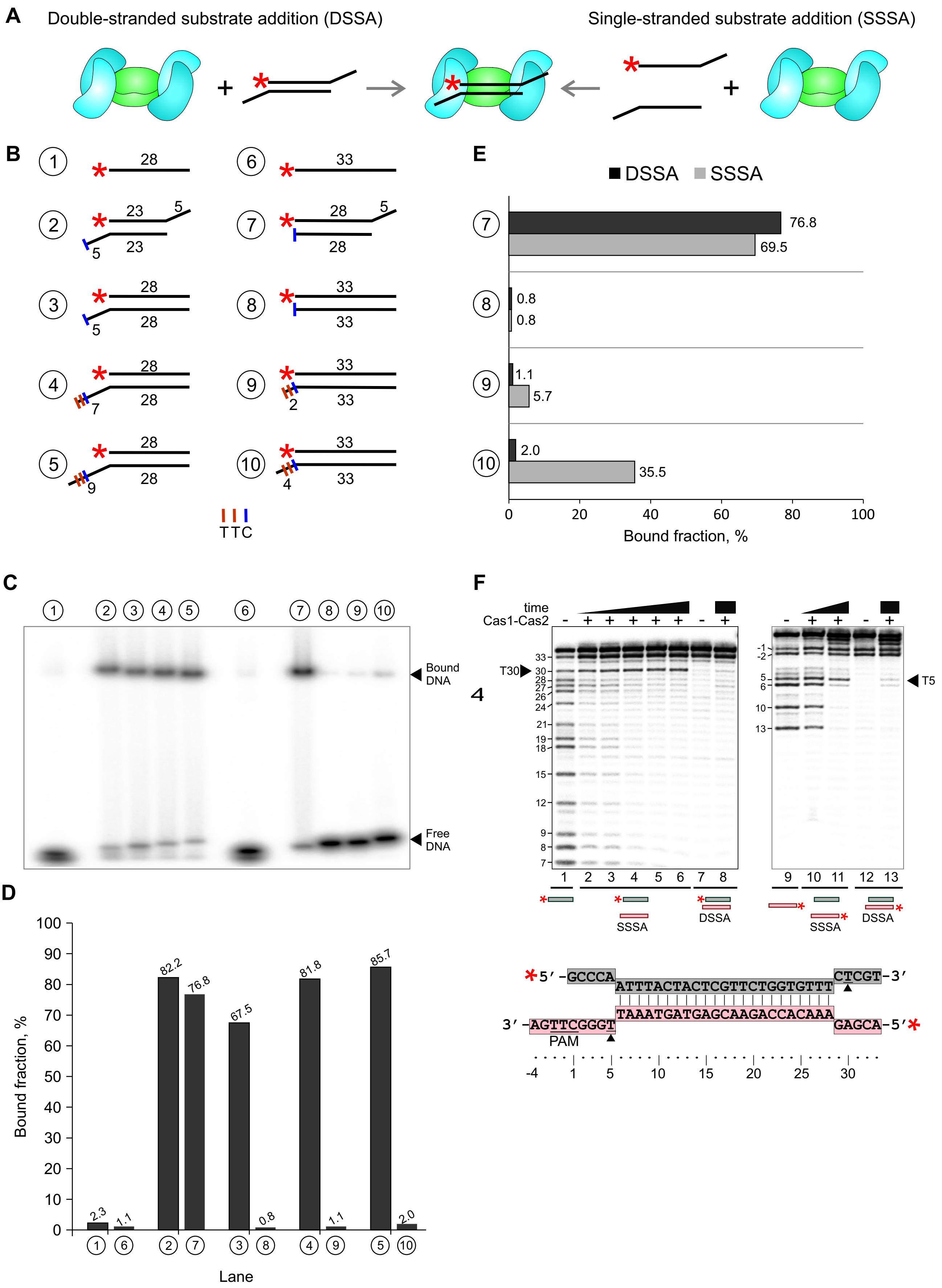
A
Double-stranded substrate addition (DSSA)
Single-stranded substrate addition (SSSA)
B
1 28
2 23 5 5 23
3 28 5 28
4 28 7 28
5 28 9 28
6 33
7 28 5 28
8 33 33
9 33 2 33
10 33 4 33
TTC
C
1 2 3 4 5 6 7 8 9 10
Bound DNA
Free DNA
D
Bound fraction, %
100 90 80 70 60 50 40 30 20 10 0
2.3 1.1 82.2 76.8 67.5 0.8 81.8 1.1 85.7 2.0
1 6 2 7 3 8 4 9 5 10
Lane
E
DSSA SSSA
7 76.8 69.5
8 0.8 0.8
9 1.1 5.7
10 2.0 35.5
0 20 40 60 80 100
Bound fraction, %
F
time
Cas1-Cas2
- + + + + + - +
33 30 28 27 26 24 21 19 18 15 12 9 8 7
T30
1 2 3 4 5 6 7 8
SSSA
DSSA
- + + - +
-1 -2 5 6 10 13
T5
9 10 11 12 13
SSSA
DSSA
5′-GCCCA ATTTACTACTCGTTCTGGTGTTT CTCGT-3′
3′-AGTTCGGGT TAAATGATGAGCAAGACCACAAA GAGCA-5′
PAM
-4 1 5 10 15 20 25 30

Fig. S9. ***In vitro*** **Cas1-Cas2 complex formation with various binding substrates.** (A) A schematic of the experimental setup. Cas1-Cas2 is mixed with preannealed double-stranded substrates (DSSA) or complementary single-stranded substrates (SSSA). In both cases only one strand of the substrate is 5'-terminally labeled with [P^{32}] (red asterisk). (B) DNA substrates used in EMSA experiments. The length of the labeled strand was constant (28 nt for substrates 1-5 or 33 nt for substrates 6-10) while the length of the complementary strand was varied. The position of the 3'-TTC-5' sequence is marked by blue and brown lines. (C) A representative autoradiograph of native acrylamide gel showing the binding of Cas1-Cas2 to indicated DSSA substrates. (D) Quantification of Cas1-Cas2-DNA complex formation. Substrates with identical unlabeled bottom strands but different labeled top strands are grouped in pairs. (E) Quantification of complex formation between Cas1-Cas2 and substrates 7-10 in DSSA and SSSA reactions (30 min). (F) KMnO4 probing was used to monitor conformational changes in the 33/37-nt DNA substrate number 10 in Cas1-Cas2 binding reactions using SSSA (lanes 2-6, 10, 11) or DSSA (lanes 8, 13) protocols. After the indicated times of Cas1-Cas2 binding to DNA (0.25, 0.5, 0.75, 1, 2 min – lanes 2-6, respectively, 0.5 min – lane 10, 10 min – lanes 8, 11, 13), $KMnO_4$ was added for 10 sec to oxidize unpaired thymines, which were revealed, after piperidine treatment, by denaturing 12%-PAGE and phosphorimaging. Based on the gel data, a schematic diagram (bottom panel) summarizing secondary structure of the DNA substrate bound by Cas1-Cas2 has been proposed. The base pairs on the diagram are numbered from the first spacer position: G in the top strand (gray colored), or C in the bottom strand (pink colored). Accordingly, thymines numbered 7-9,12,15,18,19,21,26-28 in the bottom strand (see left gel, lane 1 for reference) and 6,10,13 in the top strand (see right gel, lane 9 for reference) get gradually annealed during Cas1-Cas2-DNA complex formation (see transitions between lanes 2 and 6, and lanes 10 and 11) and become resistant to the oxidation between 6 and 28 positions (23-bp duplex formation); and sensitivity of the 5th and 30th thymines (pointed by triangles on the gels and diagram) indicates single-strand regions of DNA substrate remaining unpaired.

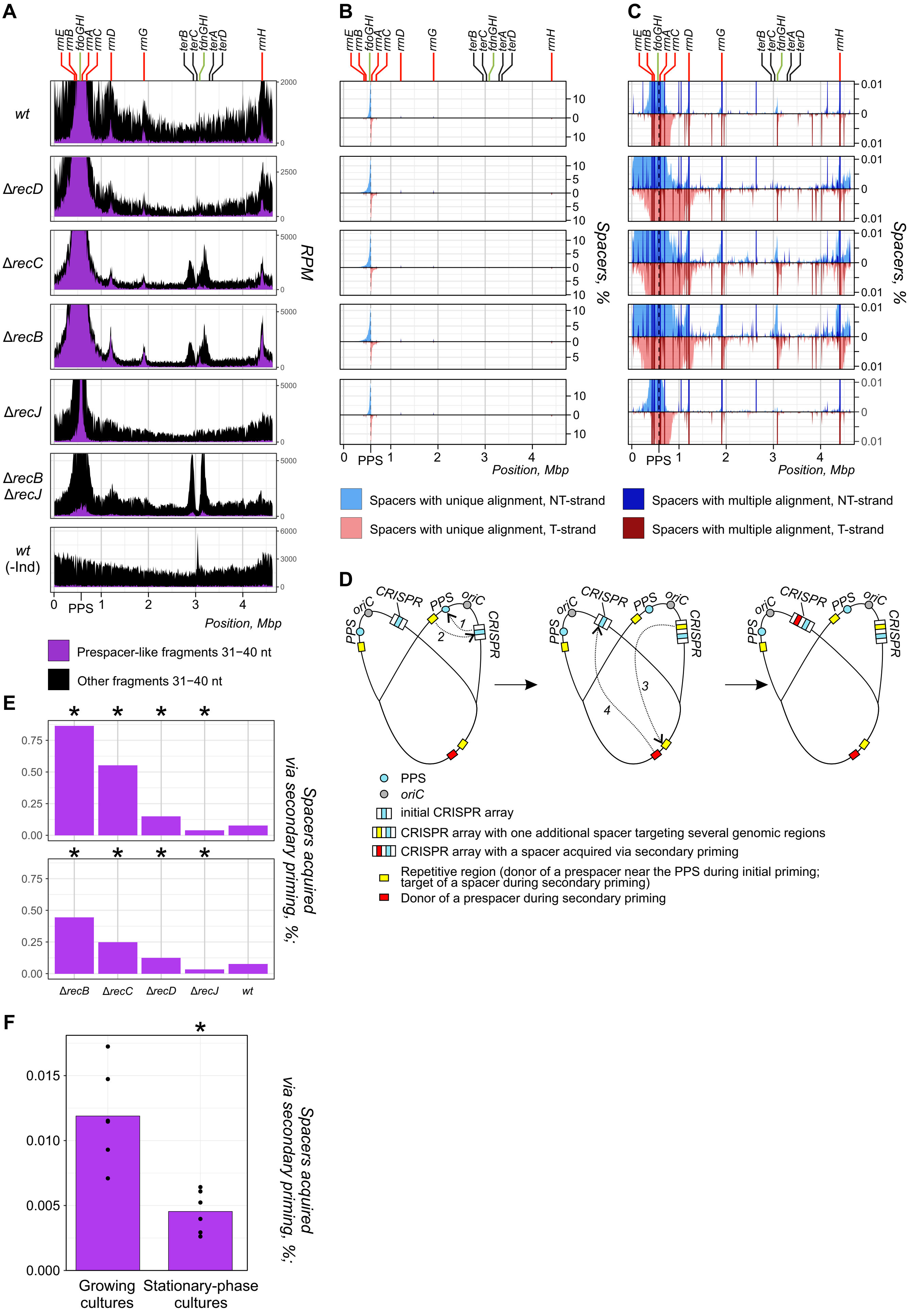
A
B
C
rrnE
rrnB
fdoGHI
rrnA
rrnC
rrnD
rrnG
terB
terC
fdnGHI
terA
terD
rrnH
wt
ΔrecD
ΔrecC
ΔrecB
ΔrecJ
ΔrecB ΔrecJ
wt (-Ind)
RPM
Spacers, %
PPS
Position, Mbp
Prespacer-like fragments 31−40 nt
Other fragments 31−40 nt
Spacers with unique alignment, NT-strand
Spacers with unique alignment, T-strand
Spacers with multiple alignment, NT-strand
Spacers with multiple alignment, T-strand
D
CRISPR
oriC
PPS
PPS
oriC
initial CRISPR array
CRISPR array with one additional spacer targeting several genomic regions
CRISPR array with a spacer acquired via secondary priming
Repetitive region (donor of a prespacer near the PPS during initial priming; target of a spacer during secondary priming)
Donor of a prespacer during secondary priming
E
Spacers acquired via secondary priming, %;
ΔrecB
ΔrecC
ΔrecD
ΔrecJ
wt
F
Growing cultures
Stationary-phase cultures

Fig. S10. Secondary priming in repetitive genomic regions. (A) Normalized sequence coverage by 31-40-nt fragments (RPM, in 10-Kbp bins). The figure is the same as Fig. 3A but zoomed in on the Y-axes. (B) Alignment of new spacers extracted from CRISPR arrays that acquired only one spacer during self-targeting of the *E. coli* genome. The number of new spacers mapped to 10-Kbp bins of the genome is shown. New spacers mapped to single locations on the chromosome are shown in light blue for the NT-strand and in pink for the T-strand. New spacers that map to several positions on the chromosome are shown in navy blue (when mapped to the NT-strand) and in maroon (when mapped to the T-strand). (C) As in B but zoomed in on the Y-axes. In A, B, and C, coordinates on the X-axes represent positions on the chromosome. *rrnA-E*, *G*, and *H* - ribosomal RNA operons; *terA*, *terB*, *terC*, *terD* - sites of replication termination; *fdoGHI* and *fdnGHI* - duplicated operons encoding formate dehydrogenase enzymes. (D) Schematic representation of a possible mechanism for the secondary priming detected in CRISPR arrays with a single newly acquired spacer. 1) The self-targeting spacer initiates primed adaptation from the PPS-region that contains a repetitive genomic region shown in yellow. 2) A "yellow" prespacer originating from this non-unique PPS-adjacent region gets incorporated into the CRISPR array. 3) The newly acquired spacer initiates primed adaptation from a matching genomic region whose location is different from the one from which the new spacer originated from. 4) A prespacer from a unique genomic sequence shown in red gets acquired into the CRISPR array. Since dividing cells have the *ori*/*ter* ratio of ~2 or higher several CRISPR arrays exist in some cells, and the "red" prespacer selected during secondary priming might get incorporated into the initial CRISPR array without the new "yellow" spacer. (E) Percent of spacers uniquely mapped to the regions 25 Kbp to the left and 25 Kbp to the right of *rrnD*, *rrnG*, *rrnH*, and *fdnGHI* in the *wt*, Δ*recB*, Δ*recC*, Δ*recD*, and Δ*recJ* strains. A 100% value corresponds to the total number of spacers uniquely mapped to the genome. The results of two biological replicates of the experiment performed in (*34*) are shown. * indicates $p<0.05$ for a Chi-square test comparing the amounts of spacers in secondary priming (25 Kbp to the left and 25 Kbp to the right of *rrnD*, *rrnG*, *rrnH*, and *fdnGHI*) and all other regions between the *wt* and other samples. (F) Percent of spacers uniquely mapped to the regions 25 Kbp to the left and 25 Kbp to the right of *rrnD*, *rrnG*, *rrnH*, and *fdnGHI* in cells with primed adaptation induced either in growing or stationary-phase cultures of *wt* self-targeting cells. A 100% value corresponds to the total number of spacers uniquely mapped to the genome. An asterisk indicates statistically significant difference between exponential and stationary-phase cells ($p<0.05$, T-test). Results of 6 independent biological replicates are presented.

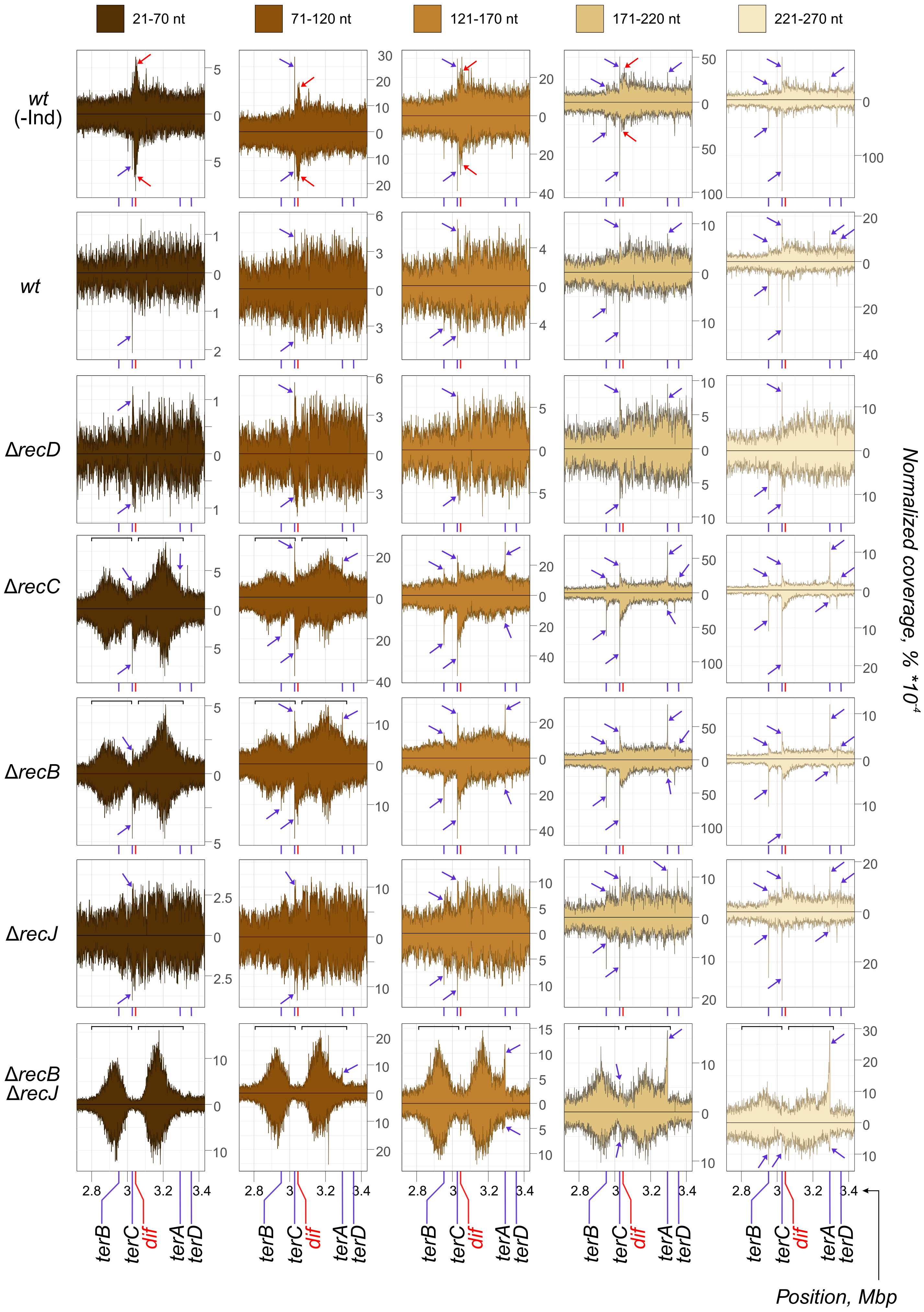

21-70 nt
71-120 nt
121-170 nt
171-220 nt
221-270 nt
wt (-Ind)
wt
ΔrecD
ΔrecC
ΔrecB
ΔrecJ
ΔrecB ΔrecJ
Normalized coverage, % *10-4
2.8
3
3.2
3.4
terB
terC
dif
terA
terD
Position, Mbp

Fig. S11. Distribution of 21-270-nt fragments mapping to the terminus region of the chromosome. Normalized sequence coverage of 21-270-nt FragSeq fragments is shown in 1-Kbp bins (100% corresponds to the total coverage with all 21-520-nt fragments mapped to the entire genome). Fragments were divided by size into groups shown in different colors. Coverage shown above and below the 0 value on the Y axis corresponds to the coverage on the top and bottom DNA strands. Red arrows mark peaks of fragments centered at *dif*. Black brackets mark two peaks of fragments located ~150 Kbp from *dif* in Δ*recB*, Δ*recC*, and Δ*recB* Δ*recJ* strains. Blue arrows mark peaks of fragments adjacent to *ter* sequences.

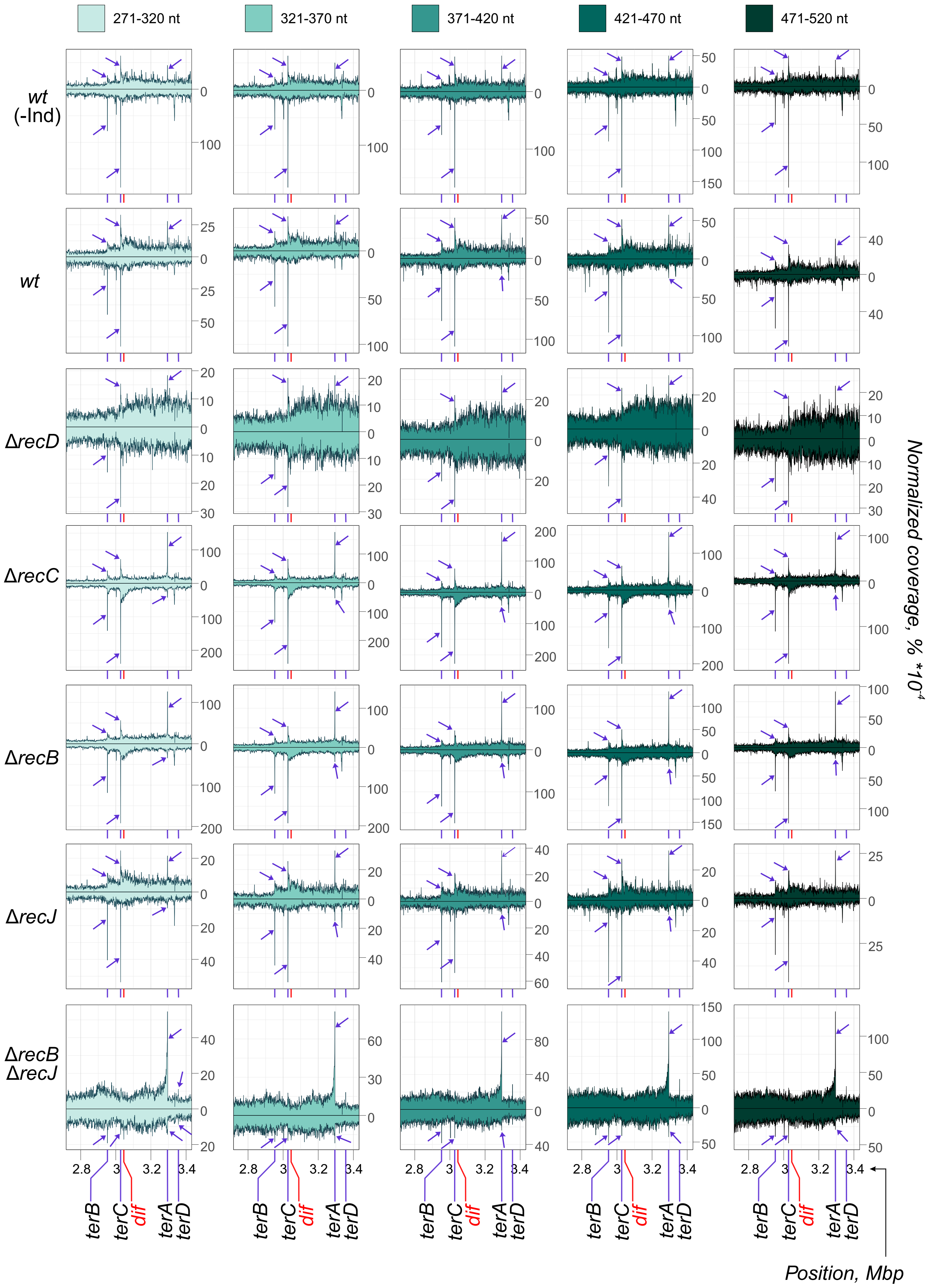

271-320 nt
321-370 nt
371-420 nt
421-470 nt
471-520 nt
wt (-Ind)
wt
ΔrecD
ΔrecC
ΔrecB
ΔrecJ
ΔrecB ΔrecJ
terB
terC
dif
terA
terD
Normalized coverage, % *10^-4
Position, Mbp

Fig. S12. Distribution of 271-520-nt fragments mapping to the terminus region of the chromosome. Normalized sequence coverage of 271-520-nt FragSeq fragments is shown in 1-Kbp bins (100% corresponds to the total coverage with all 21-520-nt fragments mapped to the entire genome). Fragments were divided by size into groups shown in different colors and analyzed separately. Coverage shown above and below the 0 value on the Y axis corresponds to the coverage on the top and bottom DNA strands. Red arrows mark peaks of fragments centered at *dif*. Blue arrows mark peaks of fragments adjacent to *ter* sequences.

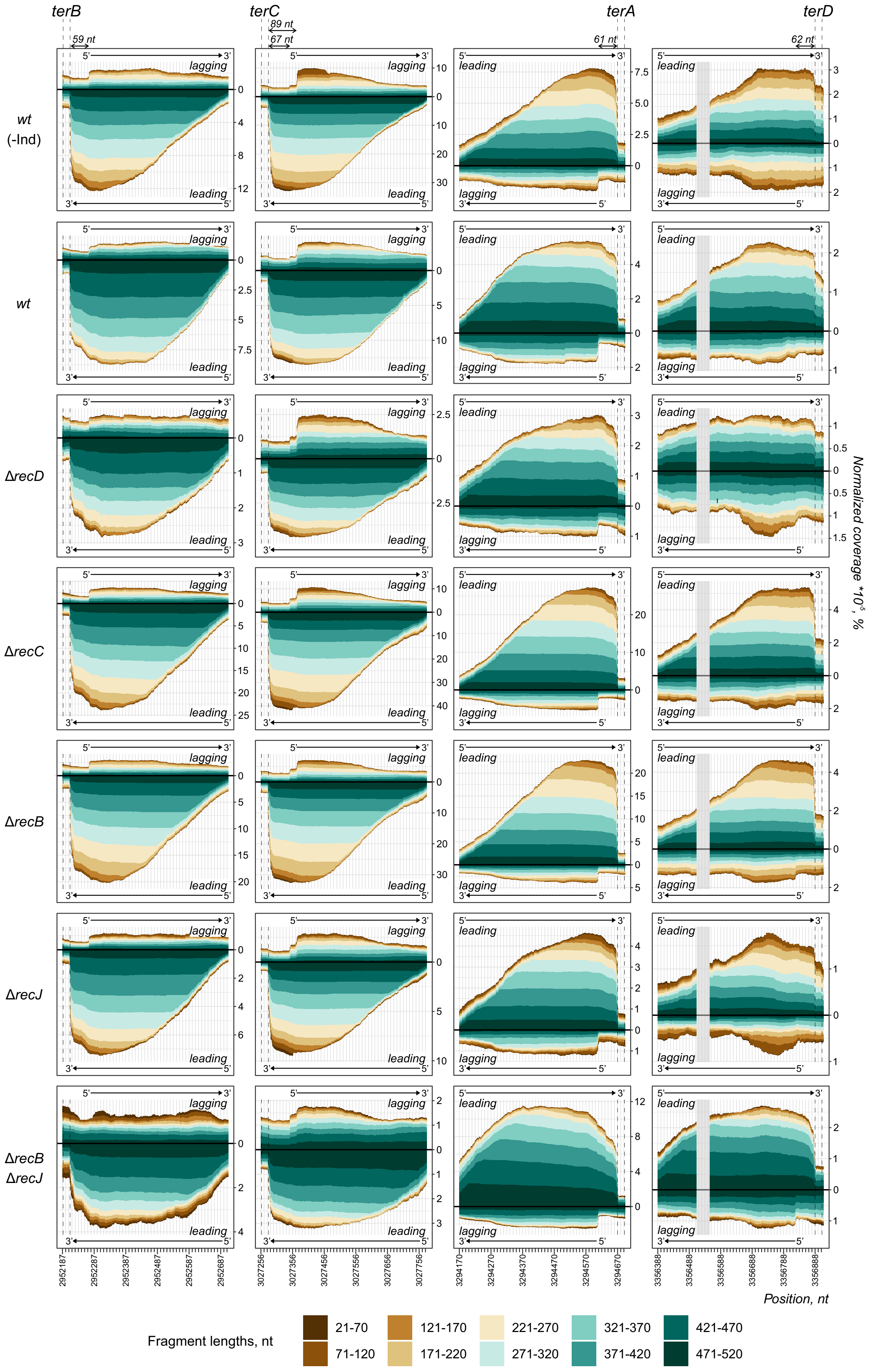

terB
terC
terA
terD
59 nt
89 nt
67 nt
61 nt
62 nt
wt (-Ind)
wt
ΔrecD
ΔrecC
ΔrecB
ΔrecJ
ΔrecB ΔrecJ
leading
lagging
5'
3'
Normalized coverage *10^5, %
2952187
2952287
2952387
2952487
2952587
2952687
3027256
3027356
3027456
3027556
3027656
3027756
3294170
3294270
3294370
3294470
3294570
3294670
3356388
3356488
3356588
3356688
3356788
3356888
Position, nt
Fragment lengths, nt
21-70
71-120
121-170
171-220
221-270
271-320
321-370
371-420
421-470
471-520

Fig. S13. Distribution of fragments mapping to *ter* sites and their nonpermissive sides. Normalized sequence coverage with FragSeq fragments is shown with 1-nt resolution (100% corresponds to the total coverage with all 21-520-nt fragments mapped to the entire genome). Depending on the *ter* site, coverage on either the leading or the lagging strand is shown above or below the 0 value on the Y axes. The direction of the leading and lagging strands for each *ter* site is shown by horizontal arrows. Colors of different shade indicate fragments of different lengths (shown below and matching colors in Supplementary Figures 11 and 12). Gray shaded areas in *terD* panels correspond to a non-unique genomic region that was removed from analysis. Dashed vertical lines indicate the boundaries of each *ter* site. The X axes show genome coordinates. Thin vertical lines shown on each panel reflect 10-nt increments of genomic coordinates.

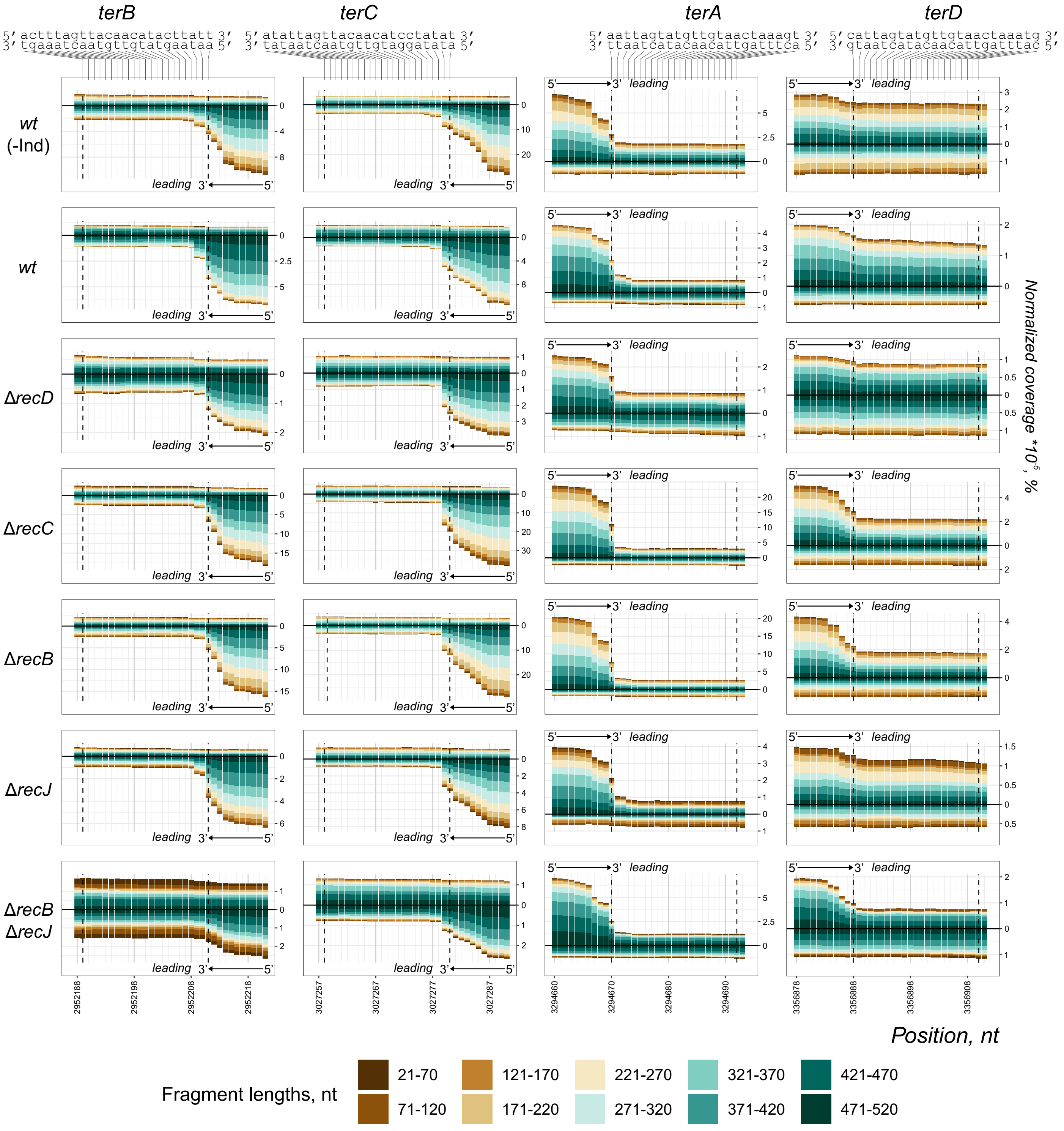

Fig. S14. A close-up view of fragments mapping to *ter* sequences. Representations are as in Supplementary Figure 13. Thin vertical lines shown on each panel reflect 1-nt increments of genomic coordinates. The ends of lagging strands are not seen at this magnification.

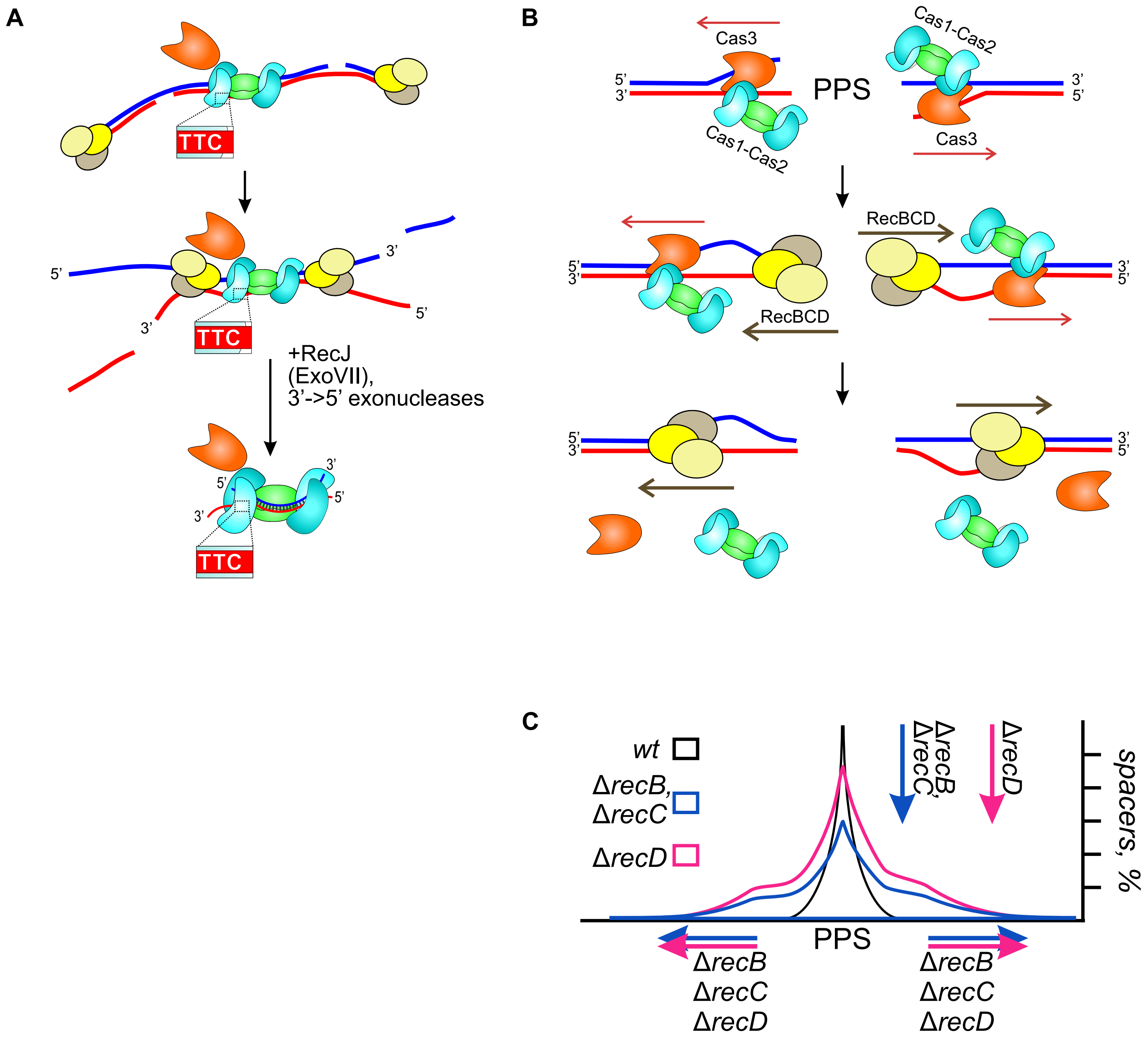

Fig. S15. A model for RecBCD involvement in two stages of primed adaptation. (A) RecBCD or RecBC unwinds double-stranded regions of prespacer precursors bound by Cas1-Cas2 ensuring processing of ends by single-strand specific exonucleases. More details are in Figure 5. (B) RecBCD dislodges a PAC which is moving away from the PPS while searching for protospacers. Thus, the presence of RecBCD limits the area from which protospacers can be selected. (C) Two outcomes of RecBCD inactivation in self-targeting cells. The peak of acquired spacers mapped to the PPS-regions becomes lower due to decreased prespacer generation efficiency and wider due to PAC movement to greater distances from PPS. The deletions of *recB* or *recC* lead to a greater decrease in prespacer generation efficiency than the *recD* deletion.

Name	Description	Source
KD403	K-12 F^+, *lacUV5-cas3 araBp8-cse1*, CRISPRI:: repeat-Sp^{yihN}-repeat, ΔCRISPRII. Sp^{yihN} (TCAAACAACCGACCTTGTTGTTCGCTATTGCC) targets chromosomal protospacer PPS (CCAAACAACCGACCTTGTTGTTCGCTATTGCC) within *yihN* gene forming a mismatch between crRNA and PPS at position +1.	Shiriaeva et al., 2019 *(24)*
ES9	KD403 Δ*recB::FRT*	Kurilovich et al., 2019 *(34)*
ES13	KD403 Δ*recC::FRT*	
KD705	KD403 Δ*recD::FRT*	
KD403 *recJ*	KD403 Δ*recJ::FRT*	
ES9 *recJ*	KD403 Δ*recB::FRT* Δ*recJ::FRT-kanR-FRT*	
KD403 *xseA*	KD403 Δ*xseA::FRT*	This study
KD403 *recJ xseA*	KD403 Δ*recJ::FRT* Δ*xseA::FRT-kanR-FRT*	
KD403 *rnt*	KD403 Δ*rnt::FRT-kanR-FRT*	
KD403 *dnaQ*	KD403 Δ*dnaQ::FRT-kanR-FRT*	
KD403 *dnaQ rnt*	KD403 Δ*rnt::FRT* Δ*dnaQ::FRT-kanR-FRT*	
KD403 *recQ*	KD403 Δ*recQ::FRT*	
KD403 *recB recQ*	KD403 Δ*recB::FRT* Δ*recQ::FRT-kanR-FRT*	
BL21-AI *recB recJ*	BL21-AI Δ*recB::FRT* Δ*recJ::FRT-kanR-FRT*	
BL21-AI *recJ xseA*	BL21-AI Δ*recJ::FRT* Δ*xseA::FRT-kanR-FRT*	
BL21-AI	F^- *ompT hsdSB (rB- mB-) gal dcm araB::T7RNAP-tetA*	Invitrogen

Table S1.

Strains used in this study

Strain	Biological replicate	Total number of sequenced CRISPR arrays with:				CRISPR arrays, total	New spacers, total	Adaptation efficiency (Number of newly acquired spacers / Number of CRISPR arrays)	Mean ± SEM
		0 newly acquired spacers	1 newly acquired spacer	2 newly acquired spacers	3 newly acquired spacers				
wt	1	151934	12900	405	0	165239	13710	0.082971	0.111±0.008
	2	161586	20384	755	1	182726	21897	0.119835	
	3	161616	23697	1055	1	186369	25810	0.138489	
	4	149947	18307	542	0	168796	19391	0.114878	
	5	153331	17574	585	3	171493	18753	0.109351	
	6	150297	16106	507	0	166910	17120	0.102570	
Δ*xseA*	1	170564	14619	500	0	185683	15619	0.084116	0.119±0.009
	2	164096	22818	973	0	187887	24764	0.131803	
	3	158946	22019	727	8	181700	23497	0.129318	
	4	143944	22475	795	1	167215	24068	0.143934	
	5	150080	19434	660	1	170175	20757	0.121974	
	6	43582	4587	152	0	48321	4891	0.101219	
Δ*recD*	1	150610	7574	118	0	158302	7810	0.049336	0.071±0.007
	2	156528	11844	307	0	168679	12458	0.073856	
	3	154732	13485	315	0	168532	14115	0.083753	
	4	134549	12842	279	1	147671	13403	0.090763	
	5	150656	7539	124	0	158319	7787	0.049186	
	6	121163	9871	248	1	131283	10370	0.078990	
Δ*recB*	1	152197	3498	41	0	155736	3580	0.022988	0.037±0.004
	2	187031	7915	146	0	195092	8207	0.042067	
	3	178232	9131	156	1	187520	9446	0.050373	
	4	172423	7089	112	0	179624	7313	0.040713	
	5	127268	4314	60	0	131642	4434	0.033682	
	6	133625	4110	83	0	137818	4276	0.031026	
Δ*recC*	1	178578	4293	80	0	182951	4453	0.024340	0.032±0.002
	2	159743	4821	92	0	164656	5005	0.030397	
	3	200672	6745	118	0	207535	6981	0.033638	

	4	176094	6350	104	0	182548	6558	0.035925	
	5	138003	3973	48	0	142024	4069	0.028650	
	6	133629	5218	96	0	138943	5410	0.038937	
ΔrecJ	1	117949	1206	6	0	119161	1218	0.010221	0.014±0.002
	2	173748	1286	3	0	175037	1292	0.007381	
	3	168638	1800	3	0	170441	1806	0.010596	
	4	164148	3475	41	0	167664	3557	0.021215	
	5	132786	2312	9	0	135107	2330	0.017246	
	6	100220	1998	8	0	102226	2014	0.019701	
ΔrecB ΔrecJ	1	3619397	6220	10	0	3625627	6240	0.001721	0.0014±0.0002
	2	3973264	7637	8	0	3980909	7653	0.001922	
	3	4093398	5616	4	0	4099018	5624	0.001372	
	4	3698279	3913	5	0	3702197	3923	0.001060	
	5	3463492	4184	1	0	3467677	4186	0.001207	
	6	2904317	3084	2	0	2907403	3088	0.001062	
ΔrecJ ΔxseA	1	15558720	1904	1	0	15560625	1906	0.000122	0.00015±0.00002
	2	19585843	1388	1	0	19587232	1390	0.000071	
	3	15266465	2875	1	0	18985489	2877	0.000152	
	4	17035251	2987	1	0	17038239	2989	0.000175	
	5	17083641	3054	0	0	17086695	3054	0.000179	
	6	18348887	3562	0	0	18352449	3562	0.000194	

Table S2.

Primed adaptation efficiency in *wt*, Δ*xseA*, Δ*recD,* Δ*recB,* Δ*recC,* Δ*recJ,* Δ*recB* Δ*recJ,* and Δ*recJ* Δ*xseA* strains

Strain 1	Strain 2	p-value	adjusted p-value (BH)
wt	Δ*recD*	3.16E-03	**3.54E-03**
wt	Δ*recB*	3.30E-05	**1.47E-04**
wt	Δ*recC*	6.64E-05	**1.86E-04**
wt	Δ*recJ*	1.89E-05	**1.47E-04**
wt	Δ*recB* Δ*recJ*	2.73E-05	**1.47E-04**
wt	Δ*xseA*	5.45E-01	5.45E-01
wt	Δ*recJ* Δ*xseA*	2.60E-05	**1.47E-04**
Δ*recD*	Δ*recB*	3.47E-03	**3.74E-03**
Δ*recD*	Δ*recC*	2.21E-03	**2.73E-03**
Δ*recD*	Δ*recJ*	2.96E-04	**4.60E-04**
Δ*recD*	Δ*recB* Δ*recJ*	2.05E-04	**4.10E-04**
Δ*recD*	Δ*xseA*	2.24E-03	**2.73E-03**
Δ*recD*	Δ*recJ* Δ*xseA*	1.89E-04	**4.08E-04**
Δ*recB*	Δ*recC*	3.13E-01	3.24E-01
Δ*recB*	Δ*recJ*	1.11E-03	**1.56E-03**
Δ*recB*	Δ*recB* Δ*recJ*	2.75E-04	**4.52E-04**
Δ*recB*	Δ*xseA*	8.02E-05	**2.04E-04**
Δ*recB*	Δ*recJ* Δ*xseA*	2.36E-04	**4.40E-04**
Δ*recC*	Δ*recJ*	2.54E-04	**4.45E-04**
Δ*recC*	Δ*recB* Δ*recJ*	2.92E-05	**1.47E-04**
Δ*recC*	Δ*xseA*	1.29E-04	**3.01E-04**
Δ*recC*	Δ*recJ* Δ*xseA*	2.54E-05	**1.47E-04**
Δ*recJ*	Δ*recB* Δ*recJ*	2.54E-03	**2.96E-03**
Δ*recJ*	Δ*xseA*	4.40E-05	**1.47E-04**
Δ*recJ*	Δ*recJ* Δ*xseA*	1.72E-03	**2.29E-03**
Δ*recB* Δ*recJ*	Δ*xseA*	4.74E-05	**1.47E-04**
Δ*recB* Δ*recJ*	Δ*recJ* Δ*xseA*	3.32E-04	**4.89E-04**
Δ*xseA*	Δ*recJ* Δ*xseA*	4.51E-05	**1.47E-04**

Table S3.

Comparison of primed adaptation efficiency between *wt*, Δ*xseA*, Δ*recD*, Δ*recB*, Δ*recC*, Δ*recJ*, Δ*recB* Δ*recJ*, and Δ*recJ* Δ*xseA* strains using the pairwise Welch's t-test (adjusted p-values < 0.05 are shown in bold)

Strain	Biological replicate	Total number of sequenced CRISPR arrays with:				CRISPR arrays, total	New spacers, total	Adaptation efficiency (Number of newly acquired spacers / Number of CRISPR arrays)	Mean ± SEM
		**0 newly acquired spacers**	**1 newly acquired spacer**	**2 newly acquired spacers**	**3 newly acquired spacers**				
wt	1	101442	7717	168	0	109327	8053	0.0737	0.092±0.006
	2	64676	5086	115	0	69877	5316	0.0761	
	3	205821	22445	718	1	228985	23884	0.1043	
	4	121514	13072	414	0	135000	13900	0.1030	
	5	90668	10083	269	0	101020	10621	0.1051	
	6	136839	12838	358	0	150035	13554	0.0903	
Δ*recB*	1	330198	4125	32	0	334355	4189	0.0125	0.027±0.004
	2	235895	6851	80	0	242826	7011	0.0289	
	3	373690	11281	127	4	385102	11547	0.0300	
	4	362634	15217	228	0	378079	15673	0.0415	
	5	159604	3307	28	0	162939	3363	0.0206	
	6	96369	2438	31	0	98838	2500	0.0253	
Δ*recQ*	1	79587	5375	116	0	85078	5607	0.0659	0.072±0.008
	2	108101	12470	337	0	120908	13144	0.1087	
	3	141549	9723	191	0	151463	10105	0.0667	
	4	46235	2863	49	0	49147	2961	0.0602	
	5	60324	3488	64	0	63876	3616	0.0566	
	6	39039	2956	68	0	42063	3092	0.0735	
Δ*recB* Δ*recQ*	1	825720	2883	9	0	828612	2901	0.0035	0.004±0.0004
	2	1393711	4532	4	0	1398247	4540	0.0032	
	3	340305	851	1	0	341157	853	0.0025	
	4	847326	3573	10	0	850909	3593	0.0042	
	5	939641	3522	10	0	943173	3542	0.0038	
	6	772504	4205	10	0	776719	4225	0.0054	

Table S4.

Primed adaptation efficiency in *wt*, Δ*recQ*, Δ*recB*, and Δ*recB* Δ*recQ* strains

Strain 1	Strain 2	p-value	adjusted p-value (BH)
wt	Δ*recQ*	6.68E-02	6.68E-02
wt	Δ*recB*	8.13E-06	**4.88E-05**
wt	Δ*recB* Δ*recQ*	2.23E-05	**6.69E-05**
Δ*recB*	Δ*recQ*	9.79E-04	**1.47E-03**
Δ*recB*	Δ*recB* Δ*recQ*	2.19E-03	**2.63E-03**
Δ*recQ*	Δ*recB* Δ*recQ*	3.04E-04	**6.07E-04**

Table S5.

Comparison of primed adaptation efficiency between *wt*, Δ*recQ*, Δ*recB*, and Δ*recB* Δ*recQ* strains using the pairwise Welch's t-test (adjusted p-values < 0.05 are shown in bold)

Strain	Biological replicate	Total number of fragments	Fragments 31-40 nt (total number per genome)	Prespacer-like 31-40-nt fragments (total number per genome)	Prespacer-like 31-40-nt fragments from the 1-Mbp PPS-region	Prespacer-like 31-40-nt fragments from the 0.1-Mbp PPS-region	Prespacer-like 31-40-nt fragments from the control 1-Mbp region	Results of the Chi-square test (BH adjusted p-values). Comparison of proportions of prespacer-like fragments in the 1-Mbp PPS/1-Mbp control region to the proportion observed in *wt*	Results of the Chi-square test (BH adjusted p-values). Comparison of proportions of prespacer-like fragments in the 0.1-Mbp PPS/1-Mbp control region to the proportion observed in *wt*
wt	1	5425090	41057	17636	16365	14035	185	NA	NA
Δ*recD*	1	5373705	41260	18139	16303	7974	206	0.29	**1.8e-10**
Δ*recB*	1	10317142	82412	47007	37835	8337	651	**6.6e-07**	**1.2e-122**
Δ*recC*	1	6184099	64772	34090	29225	12512	512	**5.9e-07**	**6.4e-42**
Δ*recJ*	1	7165761	104221	17261	14919	11830	511	**1.3e-40**	**2.6e-46**
Δ*recB* Δ*recJ*	1	5337195	151211	10252	4994	1223	1042	**0**	**0**
wt (without *cas* gene expression)	1	4933773	42707	1897	510	52	316	**0**	**0**

Table S6.

Statistics of sequencing short DNA fragments generated during primed adaptation in *wt*, Δ*recD*, Δ*recB*, Δ*recC*, Δ*recJ*, and Δ*recB* Δ*recJ* strains (adjusted p-values < 0.05 are shown in bold)

Strand	Strain1	Strain2	Sample size1	Sample size2	PAM-derived end				PAM-distal end			
					Effect size	BH-adjusted p-value, two-sided pairwise Wilcoxon rank sum test	BH-adjusted p-value, one-sided pairwise Wilcoxon rank sum test (Alternative: sample 1 > sample 2)	BH-adjusted p-value, one-sided pairwise Wilcoxon rank sum test (Alternative: sample 1 < sample 2)	Effect size	BH-adjusted p-value, two-sided pairwise Wilcoxon rank sum test	BH-adjusted p-value, one-sided pairwise Wilcoxon rank sum test (Alternative: sample 1 > sample 2)	BH-adjusted p-value, one-sided pairwise Wilcoxon rank sum test (Alternative: sample 1 < sample 2)
AAG-associated strand	Δ*recC*	Δ*recB*	2146	1476	0.0272	0.13	0.17	1	0.0384	**0.03**	1	**0.02**
	Δ*recD*	Δ*recB*	1583	1476	**0.3336**	**1.4E-75**	1	**1.4E-75**	0.0213	0.29	1	0.24
	Δ*recD*	Δ*recC*	1583	2146	**0.3629**	**2.3E-108**	1	**2.7E-108**	0.0190	0.29	0.35	1
	Δ*recJ*	Δ*recB*	3718	1476	**0.4388**	**1.1E-218**	1	**1.2E-218**	0.0855	**1.7E-09**	1	**1.6E-09**
	Δ*recJ*	Δ*recC*	3718	2146	**0.4947**	**0**	1	**0**	0.0573	**2.2E-05**	1	**1.9E-05**
	Δ*recJ*	Δ*recD*	3718	1583	**0.2734**	**9.4E-88**	1	**1.0E-87**	0.0696	**9.1E-07**	1	**8.2E-07**
	wt	Δ*recB*	4058	1476	**0.3138**	**4.6E-120**	1	**5.9E-120**	0.0558	**6.0E-05**	1	**5.0E-05**
	wt	Δ*recC*	4058	2146	**0.3695**	**1.6E-185**	1	**1.6E-185**	0.0221	0.11	1	0.09
	wt	Δ*recD*	4058	1583	0.0009	0.97	1	0.95	0.0384	**5.6E-03**	1	**4.6E-03**
	wt	Δ*recJ*	4058	3718	**0.3177**	**3.9E-172**	**3.2E-172**	1	0.0378	**1.3E-03**	**1.6E-03**	1
TTC-associated strand	Δ*recC*	Δ*recB*	8794	4570	0.0300	**8.7E-04**	1	**7.5E-04**	0.0052	0.62	0.73	1
	Δ*recD*	Δ*recB*	5275	4570	0.0301	**4.2E-03**	1	**3.5E-03**	**0.2403**	**4.0E-125**	**3.0E-125**	1
	Δ*recD*	Δ*recC*	5275	8794	0.0012	0.94	0.99	0.95	**0.2270**	**3.7E-159**	**2.9E-159**	1
	Δ*recJ*	Δ*recB*	9764	4570	0.0041	0.69	0.78	1	**0.4005**	**0**	**0**	1
	Δ*recJ*	Δ*recC*	9764	8794	0.0353	**3.0E-06**	**3.3E-06**	1	**0.4311**	**0**	**0**	1
	Δ*recJ*	Δ*recD*	9764	5275	0.0322	**1.4E-04**	**1.6E-04**	1	**0.3149**	**0**	**0**	1
	wt	Δ*recB*	10817	4570	0.0273	**1.2E-03**	1	**9.6E-04**	**0.2283**	**9.2E-176**	**8.3E-176**	1
	wt	Δ*recC*	10817	8794	0.0013	0.92	0.99	0.95	**0.2416**	**3.9E-250**	**2.9E-250**	1
	wt	Δ*recD*	10817	5275	0.0001	0.99	1	0.95	0.0118	0.17	0.21	1
	wt	Δ*recJ*	10817	9764	0.0335	**3.0E-06**	1	**2.8E-06**	**0.3234**	**0**	1	**0**

Table S7.

Pairwise comparisons of distributions of distances from prespacer ends to protospacer end coordinates between *wt*, Δ*recD*, Δ*recB*, Δ*recC*, and Δ*recJ* strains (adjusted p-values < 0.05 and effect size values > 0.1 are shown in bold)

#	Transforming oligo names	Transforming oligo sequences
1.	33/37F	5’ P-**G**CCCAATTTACCGCGCGATCGGGTGTTTGGTGA-3’
	33/37R	3’-GG**TTC**GGGTTAAATGGCGCGCTAGCCCACAAACCACT-P5’
2.	32/36F	5’ P-CCCAATTTACCGCGCGATCGGGTGTTTGGTGA-3’
	32/36R	3’-GG**TTC**GGGTTAAATGGCGCGCTAGCCCACAAACCAC-P5’
3.	34/38F	5’ P-**AG**CCCAATTTACCGCGCGATCGGGTGTTTGGTGA-3’
	34/38R	3’-GG**TTC**GGGTTAAATGGCGCGCTAGCCCACAAACCACTG-P5’

Table S8.

Oligonucleotides used for prespacer efficiency assay

*****Nucleotides corresponding to the PAM are written in bold

Transforming oligonucleotide	Biological replicate	Batch	Total number of sequenced CRISPR arrays	Adaptation efficiency (number of newly acquired spacers from any DNA per CRISPR array, %)		Oligo acquisition efficiency (number of oligo-derived spacers per CRISPR array, %)		Acquisition efficiency of a properly processed* oligonucleotide (number of properly processed oligo-derived spacers per CRISPR array, %)			% of properly processed* oligo-derived spacers among all oligo-derived spacers	
				Per replicate	Mean ± SEM	Per replicate	Mean ± SEM	Per replicate	Mean ± SEM	Adjusted values after subtracting the mean of the measurements in each batch	Per replicate	Mean ± SEM
33/37F + 33/37R	1	1	784802	5.60	5.58±0.66	4.28	3.92±0.63	3.99	3.68±0.60	-0.5300000	93.07	93.39±0.39
	2	1	762931	6.93		4.53		4.21		-0.3100000	93.01	
	3	2	659426	6.33		4.38		4.14		-0.1450000	94.38	
	4	2	960646	6.85		5.39		5.08		0.7950000	94.31	
	5	3	764598	2.59		0.90		0.82		-2.3833333	91.88	
	6	3	722981	5.18		4.07		3.81		0.6066667	93.68	
32/36F + 32/36R	1	1	442044	8.14	7.60±0.86	6.85	5.76±0.81	6.40	5.36±0.75	1.8800000	93.46	93.01±0.20
	2	1	704759	10.23		7.31		6.76		2.2400000	92.44	
	3	2	720848	5.27		3.85		3.59		-0.6950000	93.19	
	4	2	807672	9.29		7.66		7.17		2.8850000	93.60	
	5	3	638573	5.06		2.84		2.63		-0.5733333	92.58	
	6	3	723838	7.58		6.07		5.64		2.4366667	92.78	
34/38F + 34/38R	1	1	592398	4.81	4.63±0.24	3.87	3.18±0.32	3.63	2.97±0.30	-0.8900000	93.79	93.26±0.21
	2	1	374286	4.09		2.31		2.13		-2.3900000	92.28	
	3	2	631397	3.87		2.53		2.35		-1.9350000	93.20	
	4	2	450826	4.81		3.62		3.38		-0.9050000	93.55	
	5	3	441205	4.63		2.67		2.49		-0.7133333	93.41	
	6	3	401047	5.55		4.11		3.83		0.6266667	93.32	

Table S9.

Statistics of sequencing CRISPR arrays in prespacer efficiency assay upon electroporation of oligonucleotides in BL21-AI pCas1/2 cells

***** We define properly processed oligo-derived spacers as those that were processed between an A and a G in the PAM sequence (T and C in PAM-complementary sequence) and integrated as a 33 bp spacer with the PAM-derived G being in the top CRISPR strand

Transforming oligonucleotide	Biological replicate	Batch	Total number of sequenced CRISPR arrays	Adaptation efficiency (number of newly acquired spacers from any DNA per CRISPR array, %)		Oligo acquisition efficiency (number of oligo-derived spacers per CRISPR array, %)		Acquisition efficiency of a properly processed* oligonucleotide (number of properly processed oligo-derived spacers per CRISPR array, %)		% of properly processed* oligo-derived spacers among all oligo-derived spacers		
				**Per replicate**	**Mean ± SEM**	**Per replicate**	**Mean ± SEM**	**Per replicate**	**Mean ± SEM**	**Adjusted values after subtracting the mean of the measurements in each batch**	**Per replicate**	**Mean ± SEM**
33/37F + 33/37R	1	1	303775	0.82	0.76±0.05	0.37	0.31±0.03	0.33	0.27±0.03	0.060000000	88.77	87.46± 0.49
	2	1	319710	0.55		0.16		0.13		-0.140000000	85.69	
	3	1	301449	0.88		0.38		0.34		0.070000000	87.91	
	4	2	337242	0.79		0.34		0.30		-0.034444444	88.66	
	5	2	314293	0.81		0.31		0.27		-0.064444444	86.80	
	6	2	283067	0.72		0.28		0.24		-0.094444444	86.92	
32/36F + 32/36R	1	1	319970	0.86	0.79±0.07	0.43	0.41±0.05	0.38	0.36±0.05	0.110000000	88.73	89.24± 0.53
	2	1	271738	0.49		0.19		0.17		-0.100000000	87.91	
	3	1	304344	0.82		0.47		0.43		0.160000000	90.29	
	4	2	321016	0.74		0.37		0.33		-0.004444444	87.69	
	5	2	289069	1.05		0.56		0.51		0.175555556	90.64	
	6	2	314212	0.78		0.41		0.37		0.035555556	90.17	
34/38F + 34/38R	1	1	295967	0.64	0.69±0.05	0.24	0.30±0.03	0.22	0.27±0.03	-0.050000000	90.61	90.63± 0.42
	2	1	293047	0.58		0.19		0.17		-0.100000000	89.88	
	3	1	327774	0.59		0.28		0.26		-0.010000000	92.62	
	4	2	240476	0.66		0.31		0.28		-0.054444444	90.15	
	5	2	332502	0.84		0.40		0.36		0.025555556	89.92	
	6	2	290952	0.86		0.39		0.35		0.015555556	90.61	

Table S10.

Statistics of sequencing CRISPR arrays in prespacer efficiency assay upon electroporation of oligonucleotides in BL21-AI Δ*recB* Δ*recJ* pCas1/2 cells

***** We define properly processed oligo-derived spacers as those that were processed between an A and a G in the PAM sequence (T and C in PAM-complementary sequence) and integrated as a 33 bp spacer with the PAM-derived G being in the top CRISPR strand

**Transforming oligonucleotide**	**Biological replicate**	**Batch**	**Total number of sequenced CRISPR arrays**	**Adaptation efficiency (number of newly acquired spacers from any DNA per CRISPR array, %)**		**Oligo acquisition efficiency (number of oligo-derived spacers per CRISPR array, %)**		**Acquisition efficiency of a properly processed* oligonucleotide (number of properly processed oligo-derived spacers per CRISPR array, %)**			**% of properly processed* oligo-derived spacers among all oligo-derived spacers**	
				**Per replicate**	**Mean ± SEM**	**Per replicate**	**Mean ± SEM**	**Per replicate**	**Mean ± SEM**	**Adjusted values after subtracting the mean of the measurements in each batch**	**Per replicate**	**Mean ± SEM**
33/37F + 33/37R	1	1	288708	2.28	5.10±1.34	2.15	4.84±1.28	2.05	4.60±1.22	0.148888889	95.37	94.97± 0.17
	2	1	287337	1.63		1.51		1.42		-0.481111111	94.21	
	3	1	223324	2.66		2.45		2.33		0.428888889	95.23	
	4	2	269037	7.96		7.61		7.22		-0.250000000	94.94	
	5	2	258028	9.08		8.63		8.19		0.720000000	94.88	
	6	2	297585	6.99		6.68		6.36		-1.110000000	95.18	
32/36F + 32/36R	1	1	260749	0.71	4.85±1.46	0.67	4.61±1.39	0.63	4.36±1.32	-1.271111111	94.88	94.57± 0.10
	2	1	259897	3.63		3.45		3.26		1.358888889	94.60	
	3	1	272556	1.39		1.26		1.19		-0.711111111	94.42	
	4	2	257272	9.44		8.99		8.48		1.010000000	94.25	
	5	2	278452	8.19		7.81		7.41		-0.060000000	94.82	
	6	2	274823	5.76		5.49		5.19		-2.280000000	94.44	
34/38F + 34/38R	1	1	272898	2.17	5.60±1.56	2.10	5.38±1.50	1.99	5.10±1.43	0.088888889	94.90	94.72± 0.07
	2	1	246880	2.57		2.47		2.34		0.438888889	94.62	
	3	1	275154	2.14		2.01		1.90		-0.001111111	94.60	
	4	2	301663	7.70		7.43		7.05		-0.420000000	94.88	
	5	2	319279	7.96		7.61		7.20		-0.270000000	94.51	
	6	2	196481	11.06		10.68		10.13		2.660000000	94.83	

Table S11.

Statistics of sequencing CRISPR arrays in prespacer efficiency assay upon electroporation of oligonucleotides in BL21-AI Δ*recJ* Δ*xseA* pCas1/2 cells

***** We define properly processed oligo-derived spacers as those that were processed between an A and a G in the PAM sequence (T and C in PAM-complementary sequence) and integrated as a 33 bp spacer with the PAM-derived G being in the top CRISPR strand

#	Description	Oligo sequences
1.	PAM-distal side labeled DNA substrate<br>(Fig. 4A; Fig. S5)	5'-TTAACCGGTCAACCTAAACATTTACCGCGCGATCGGGTGTTTGGTGA/36-FAM/-3′<br>5'-TCCGGGTTGCGGTGGACAAAAACACCCGATCGCGCGGTAAATTGGGCTTGG-3'
2.	PAM-proximal side labeled DNA substrate<br>(Fig. 4A)	5'-TTAACCGGTCAACCTAAACATTTACCGCGCGATCGGGTGTTTGGTGA-3′<br>5'-TCCGGGTTGCGGTGGACAAAAACACCCGATCGCGCGGTAAATTGGGCTTGG/36-FAM/-3'
3.	PAM-distal side labeled DNA substrate<br>(Fig. S6)	5′-TTAAACCATCCTGAGTTTGATTTACTACTCGTTCTGGTGTTTCTCGT/36-FAM/-3′<br>5′-GTAGAAAACACCAGAACGAGTAGTAAATTGGGCTTGA-3′
4.	PAM-distal side labeled substrate with a double-stranded extension<br>(Fig. 4B, Fig. S7)	5′-CCGCCTTAACCGGTCAACCTAAACATTTACCGCGCGATCGGGTGTTTGGTGA/36-FAM/-3′<br>5′-CACAAAAACACCCGATCGCGCGGTAAATTGGGCTTGG-3′<br>5′-GACCGGTTAAGGCGG-3′

Table S12.
Oligonucleotides used in exonuclease footprinting experiments

Strain	Biological replicate	Total number of sequenced CRISPR arrays with:				CRISPR arrays, total	New spacers, total	Adaptation efficiency (Number of newly acquired spacers / Number of CRISPR arrays)	Mean ± SEM
		0 newly acquired spacers	1 newly acquired spacer	2 newly acquired spacers	3 newly acquired spacers				
wt	1	53122	10456	529	2	64109	11520	0,18	0.21±0.014
	2	81604	19482	1098	1	102185	21681	0,21	
	3	64582	16884	948	8	82422	18804	0,23	
Δ*rnt*	1	57744	26774	1930	6	86454	30652	0,35	0.39±0.037
	2	63732	29227	2510	23	95492	34316	0,36	
	3	53430	36461	3632	27	93550	43806	0,47	
Δ*dnaQ*	1	49783	11007	587	3	61380	12190	0,20	0.20±0.002
	2	58587	13079	810	2	72478	14705	0,20	
	3	65287	13814	832	4	79937	15490	0,19	
Δ*rnt* Δ*dnaQ*	1	91854	11557	304	2	103717	12171	0,12	0.11±0.005
	2	30002	3268	72	0	33342	3412	0,10	
	3	58321	7211	194	0	65726	7599	0,12	

Table S13.

Primed adaptation efficiency in *wt*, Δ*rnt*, Δ*dnaQ*, and Δ*rnt* Δ*dnaQ* strains

Strain 1	Strain 2	p-value	adjusted p-value (BH)
Δ*dnaQ*	Δ*rnt* Δ*dnaQ*	1.58E-02	**1.89E-02**
Δ*dnaQ*	Δ*rnt*	1.30E-04	**3.40E-04**
Δ*dnaQ*	*wt*	7.79E-01	7.79E-01
Δ*rnt* Δ*dnaQ*	Δ*rnt*	8.80E-06	**5.30E-05**
Δ*rnt* Δ*dnaQ*	*wt*	1.02E-02	**1.53E-02**
Δ*rnt*	*wt*	1.70E-04	**3.40E-04**

Table S14.
Comparison of primed adaptation efficiency between *wt*, Δ*rnt*, Δ*dnaQ*, and Δ*rnt* Δ*dnaQ* strains using the pairwise T-test (adjusted p-values < 0.05 are shown in bold)

Strain	Biological replicate	Total number of fragments	Fragments 31-40 nt (total number per genome)	Prespacer-like 31-40-nt fragments (total number per genome)	Prespacer-like 31-40-nt fragments from the 1-Mbp PPS-region	Prespacer-like 31-40-nt fragments from the control 1-Mbp region	Ratio prespacer-like fragments in the PPS/control region	Mean ratio ± SEM
wt	1	436145	9413	1582	1266	75	16.88	19.62±5.29
wt	2	509175	9455	1837	1491	58	25.71	
wt	3	368867	8459	1510	1203	74	16.26	
Δ*rnt*	1	689507	41538	15329	13557	288	47.07	46.92±2.23
Δ*rnt*	2	591561	15190	4739	4194	94	44.62	
Δ*rnt*	3	782028	19949	5853	5152	105	49.07	
Δ*dnaQ*	1	549357	15419	4234	3689	150	24.59	22.28±2.78
Δ*dnaQ*	2	590292	13898	3434	2927	127	23.05	
Δ*dnaQ*	3	608118	10840	2636	2227	116	19.20	
Δ*dnaQ* Δ*rnt*	1	477421	10366	2125	1723	101	17.06	13.69±3.27
Δ*dnaQ* Δ*rnt*	2	391716	5347	914	694	66	10.52	
Δ*dnaQ* Δ*rnt*	3	784474	19428	6150	4946	367	13.48	

Table S15.

Statistics of sequencing short DNA fragments generated during primed adaptation in *wt*, Δ*rnt*, Δ*dnaQ*, and Δ*rnt* Δ*dnaQ* strains

Strain 1	Strain 2	p-value	adjusted p-value (BH)
Δ*dnaQ*	Δ*rnt* Δ*dnaQ*	1.90E-02	**2.80E-02**
Δ*dnaQ*	Δ*rnt*	3.00E-05	**6.00E-05**
Δ*dnaQ*	*wt*	3.89E-01	3.89E-01
Δ*rnt* Δ*dnaQ*	Δ*rnt*	3.20E-06	**1.90E-05**
Δ*rnt* Δ*dnaQ*	*wt*	7.70E-02	9.30E-02
Δ*rnt*	*wt*	1.40E-05	**4.30E-05**

Table S16.
Comparison of prespacer generation efficiency (ratio of prespacer-like fragments in the PPS/control region) between *wt*, Δ*rnt*, Δ*dnaQ*, and Δ*rnt* Δ*dnaQ* strains using the pairwise T-test (adjusted p-values < 0.05 are shown in bold)

Strand	Strain1	Strain2	Sample size1	Sample size2	PAM-derived end				PAM-distal end			
					**Effect size**	**BH-adjusted p-value, two-sided pairwise Wilcoxon rank sum test**	**BH-adjusted p-value, one-sided pairwise Wilcoxon rank sum test (Alternative: sample 1 > sample 2)**	**BH-adjusted p-value, one-sided pairwise Wilcoxon rank sum test (Alternative: sample 1 < sample 2)**	**Effect size**	**BH-adjusted p-value, two-sided pairwise Wilcoxon rank sum test**	**BH-adjusted p-value, one-sided pairwise Wilcoxon rank sum test (Alternative: sample 1 > sample 2)**	**BH-adjusted p-value, one-sided pairwise Wilcoxon rank sum test (Alternative: sample 1 < sample 2)**
AAG-associated strand	Δ*dnaQ* Δ*rnt*	Δ*dnaQ*	1814	2188	0.0419	**0.02**	**0.01**	1	0.0310	0.10	1	0.12
	Δ*rnt*	Δ*dnaQ*	4050	2188	0.0069	0.72	1	0.72	0.0133	0.44	0.39	1
	Δ*rnt*	Δ*dnaQ* Δ*rnt*	4050	1814	0.0466	**2.9E-03**	1	**2.1E-03**	0.0418	**5.2E-03**	**4.2E-03**	1
	wt	Δ*dnaQ*	968	2188	0.0078	0.72	0.66	1	0.0560	**5.2E-03**	1	**6.6E-03**
	wt	Δ*dnaQ* Δ*rnt*	968	1814	0.0323	0.16	1	0.18	0.0284	0.22	1	0.23
	wt	Δ*rnt*	968	4050	0.0126	0.52	0.45	1	0.0590	**3.5E-04**	1	**3.5E-04**
TTC-associated strand	Δ*dnaQ* Δ*rnt*	Δ*dnaQ*	5052	5869	0.0311	**5.2E-03**	**4.2E-03**	1	0.0056	0.72	1	0.72
	Δ*rnt*	Δ*dnaQ*	14415	5869	0.0410	**1.3E-07**	**6.5E-08**	1	0.0199	**0.01**	**9.1E-03**	1
	Δ*rnt*	Δ*dnaQ* Δ*rnt*	14415	5052	0.0106	0.22	0.21	1	0.0245	**3.8E-03**	**3.8E-03**	1
	wt	Δ*dnaQ*	2523	5869	0.0342	**5.2E-03**	**4.2E-03**	1	0.0039	0.75	1	0.78
	wt	Δ*dnaQ* Δ*rnt*	2523	5052	0.0054	0.72	0.66	1	0.0012	0.92	0.85	1
	wt	Δ*rnt*	2523	14415	0.0040	0.72	1	0.72	0.0189	**0.03**	1	**0.04**

Table S17.

Pairwise comparisons of distributions of distances from prespacer ends to protospacer end coordinates between *wt*, Δ*rnt*, Δ*dnaQ*, and Δ*rnt* Δ*dnaQ* strains (adjusted p-values < 0.05 are shown in bold)

#	Description	Oligo sequences
1	5'[P32] labeled 28 nt oligonucleotide used as top strand in double stranded prespacer substrates #2-5	5'P32-ATTTACTACTCGTTCTGGTGTTTCTCGT-3’
2	Symmetrical prespacer substrate with a central 23-bp duplex flanked by 5-nt single-stranded DNA on each 3' end	5'P32-ATTTACTACTCGTTCTGGTGTTTCTCGT-3<br>3'-CGGGTTAAATGATGAGCAAGACCACAAA-5’
3	Prespacer substrate containing a 28-bp duplex and a single 5-nt 3’-overhang	5'P32-ATTTACTACTCGTTCTGGTGTTTCTCGT-3’<br>3'-CGGGTTAAATGATGAGCAAGACCACAAAGAGCA-5’
4	Prespacer substrate with a 28-bp duplex and a single 7-nt 3’-overhang containing PAM	5'P32-ATTTACTACTCGTTCTGGTGTTTCTCGT-3’<br>3'-**TTC**GGGTTAAATGATGAGCAAGACCACAAAGAGCA-5’
5.	Prespacer substrate with a 28-bp duplex and a single 9-nt 3’-overhang containing PAM	5'P32-ATTTACTACTCGTTCTGGTGTTTCTCGT-3’<br>3'-AG**TTC**GGGTTAAATGATGAGCAAGACCACAAAGAGCA-5’
6	5'[P32] labeled 33 nt oligonucleotide used as top strand in double stranded prespacer substrates #8-10	5'P32-GCCCAATTTACTACTCGTTCTGGTGTTTCTCGT-3’
7	Prespacer substrate containing a 28-bp duplex and a single 5-nt 3’-overhang	5'P32-GCCCAATTTACTACTCGTTCTGGTGTTTCTCGT-3’<br>3'-CGGGTTAAATGATGAGCAAGACCACAAA-5’
8	Blunt-ended prespacer substrate 33 bp long	5'P32-GCCCAATTTACTACTCGTTCTGGTGTTTCTCGT-3’<br>3'-CGGGTTAAATGATGAGCAAGACCACAAAGAGCA-5’
9	Prespacer substrate with a 33-bp duplex and a single 2-nt 3’-overhang containing PAM	5'P32-**G**CCCAATTTACTACTCGTTCTGGTGTTTCTCGT-3’<br>3'-**TTC**GGGTTAAATGATGAGCAAGACCACAAAGAGCA-5’
10.	Prespacer substrate with a 33-bp duplex and a single 4-nt 3’-overhang containing PAM	5'P32-**G**CCCAATTTACTACTCGTTCTGGTGTTTCTCGT-3’<br>3'-AG**TTC**GGGTTAAATGATGAGCAAGACCACAAAGAGCA-5’

Table S18.
Oligonucleotides used in binding and KMnO4 footprinting experiments (Fig. S9)

***Nucleotides corresponding to the PAM are shown in bold

**Biological replicate**	**Strain**	**Uniquely mapped spacers from CRISPR arrays with a single new spacer**	**Spacers acquired via secondary priming (spacers uniquely mapped to the regions not farther than 25 kbp from *rrnD, rrnG, rrnH,* and *fdnGHI*)**	**Percent of spacers acquired via secondary priming**	**Results of the chi-square test (BH adjusted p-values). Comparison of the proportion of spacers acquired via secondary priming and other spacers in a mutant to the proportion observed in *wt***
1	*wt*	137046	105	0.077	NA
	Δ*recB*	100884	871	0.863	**3.68E-192**
	Δ*recC*	66223	365	0.551	**9.64E-96**
	Δ*recD*	113087	170	0.150	**6.96E-08**
	Δ*recJ*	99966	38	0.038	**0.00025**
2	*wt*	96297	73	0.076	NA
	Δ*recB*	137601	613	0.446	**7.92E-59**
	Δ*recC*	88479	219	0.248	**5.72E-20**
	Δ*recD*	159770	200	0.125	**0.00027**
	Δ*recJ*	80902	26	0.032	**0.00021**

Table S19.
Secondary priming in *wt*, Δ*recB*, Δ*recC*, Δ*recD*, and Δ*recJ strains*. The data for analysis were taken from *(34)*. Adjusted p-values < 0.05 are shown in bold

**Sample type**	**Biological replicate**	**Uniquely mapped spacers from CRISPR arrays with a single new spacer**	**Spacers acquired via secondary priming (spacers uniquely mapped to the regions not farther than 25 Kbp from *rrnD*, *rrnG*, *rrnH*, and *fdnGHI*)**	**Percent of spacers acquired via secondary priming**	**Mean ± SEM**	**T-test, p-value**
Growing cells	1	1086432	101	0.0093	0.0119±0.0015	**0.0011**
	2	563783	65	0.0115		
	3	634417	45	0.0071		
	4	493028	85	0.0172		
	5	454755	67	0.0147		
	6	427669	49	0.0115		
Stationary-phase cells	1	936639	57	0.0061	0.0045±0.0007	
	2	932303	37	0.0040		
	3	668903	35	0.0052		
	4	701043	45	0.0064		
	5	458601	12	0.0026		
	6	477428	14	0.0029		

Table S20.
Secondary priming is decreased in stationary-phase cells

Experiment	Strain/ Conditions	Biological replicate	Primer 1 (5'- 3')	Primer 2 (5'- 3')
High-throughput sequencing of spacers acquired during primed adaptation (Fig. S1A-B)	KD403	1	Au1<br>**GACTGGAGTTCAGACGTGTGCTCTTCCGATCT**GTCAGcaacaaggtcggttgtttgacg	KL1<br>**ACACTCTTTCCCTACACGACGCTCTTCCGATCT**TCGAaatgctttaagaacaaatgtatacttttagagagttc
		2		KL2<br>**ACACTCTTTCCCTACACGACGCTCTTCCGATCT**AGCTaatgctttaagaacaaatgtatacttttagagagttc
		3		KL3<br>**ACACTCTTTCCCTACACGACGCTCTTCCGATCT**GCACaatgctttaagaacaaatgtatacttttagagagttc
		4		KL4<br>**ACACTCTTTCCCTACACGACGCTCTTCCGATCT**CATCaatgctttaagaacaaatgtatacttttagagagttc
		5		KL5<br>**ACACTCTTTCCCTACACGACGCTCTTCCGATCT**ATGAaatgctttaagaacaaatgtatacttttagagagttc
		6		KL6<br>**ACACTCTTTCCCTACACGACGCTCTTCCGATCT**TGCGaatgctttaagaacaaatgtatacttttagagagttc
	KD403 Δ*recB*	1	Au6<br>**GACTGGAGTTCAGACGTGTGCTCTTCCGATCT**GCTCTcaacaaggtcggttgtttgacg	KL1<br>**ACACTCTTTCCCTACACGACGCTCTTCCGATCT**TCGAaatgctttaagaacaaatgtatacttttagagagttc
		2		KL2<br>**ACACTCTTTCCCTACACGACGCTCTTCCGATCT**AGCTaatgctttaagaacaaatgtatacttttagagagttc
		3		KL3<br>**ACACTCTTTCCCTACACGACGCTCTTCCGATCT**GCACaatgctttaagaacaaatgtatacttttagagagttc
		4		KL4<br>**ACACTCTTTCCCTACACGACGCTCTTCCGATCT**CATCaatgctttaagaacaaatgtatacttttagagagttc
		5		KL5<br>**ACACTCTTTCCCTACACGACGCTCTTCCGATCT**ATGAaatgctttaagaacaaatgtatacttttagagagttc
		6		KL6<br>**ACACTCTTTCCCTACACGACGCTCTTCCGATCT**TGCGaatgctttaagaacaaatgtatacttttagagagttc

	KD403 Δ*recC*	1	Au9<br>**GACTGGAGTTCAGACGTGTGCTCTTCCGATCT**GAGATcaacaaggtcggttgtttgacg	KL1<br>**ACACTCTTTCCCTACACGACGCTCTTCCGATCT**TCGAaatgctttaagaacaaatgtatacttttagagagttc
		2		KL2<br>**ACACTCTTTCCCTACACGACGCTCTTCCGATCT**AGCTaatgctttaagaacaaatgtatacttttagagagttc
		3		KL3<br>**ACACTCTTTCCCTACACGACGCTCTTCCGATCT**GCACaatgctttaagaacaaatgtatacttttagagagttc
		4		KL4<br>**ACACTCTTTCCCTACACGACGCTCTTCCGATCT**CATCaatgctttaagaacaaatgtatacttttagagagttc
		5		KL5<br>**ACACTCTTTCCCTACACGACGCTCTTCCGATCT**ATGAaatgctttaagaacaaatgtatacttttagagagttc
		6		KL6<br>**ACACTCTTTCCCTACACGACGCTCTTCCGATCT**TGCGaatgctttaagaacaaatgtatacttttagagagttc
	KD403 Δ*recD*	1	Au2<br>**GACTGGAGTTCAGACGTGTGCTCTTCCGATCT**GGTTGcaacaaggtcggttgtttgacg	KL1<br>**ACACTCTTTCCCTACACGACGCTCTTCCGATCT**TCGAaatgctttaagaacaaatgtatacttttagagagttc
		2		KL2<br>**ACACTCTTTCCCTACACGACGCTCTTCCGATCT**AGCTaatgctttaagaacaaatgtatacttttagagagttc
		3		KL3<br>**ACACTCTTTCCCTACACGACGCTCTTCCGATCT**GCACaatgctttaagaacaaatgtatacttttagagagttc
		4		KL4<br>**ACACTCTTTCCCTACACGACGCTCTTCCGATCT**CATCaatgctttaagaacaaatgtatacttttagagagttc
		5		KL5<br>**ACACTCTTTCCCTACACGACGCTCTTCCGATCT**ATGAaatgctttaagaacaaatgtatacttttagagagttc
		6		KL6<br>**ACACTCTTTCCCTACACGACGCTCTTCCGATCT**TGCGaatgctttaagaacaaatgtatacttttagagagttc
	KD403 Δ*recJ*	1	Au3<br>**GACTGGAGTTCAGACGTGTGCTCTTCCGATCT**CCAGCcaacaaggtcggttgtttgacg	KL1<br>**ACACTCTTTCCCTACACGACGCTCTTCCGATCT**TCGAaatgctttaagaacaaatgtatacttttagagagttc

		2		KL2<br>**ACACTCTTTCCCTACACGACGCTCTTCCGATCT**AGCTaatgctttaagaacaaatgtatacttttagagagttc
		3		KL3<br>**ACACTCTTTCCCTACACGACGCTCTTCCGATCT**GCACaatgctttaagaacaaatgtatacttttagagagttc
		4		KL4<br>**ACACTCTTTCCCTACACGACGCTCTTCCGATCT**CATCaatgctttaagaacaaatgtatacttttagagagttc
		5		KL5<br>**ACACTCTTTCCCTACACGACGCTCTTCCGATCT**ATGAaatgctttaagaacaaatgtatacttttagagagttc
		6		KL6<br>**ACACTCTTTCCCTACACGACGCTCTTCCGATCT**TGCGaatgctttaagaacaaatgtatacttttagagagttc
	KD403<br>Δ*xseA*	1	Au4<br>**GACTGGAGTTCAGACGTGTGCTCTTCCGATCT**TACCGcaacaaggtcggttgtttgacg	KL1<br>**ACACTCTTTCCCTACACGACGCTCTTCCGATCT**TCGAaatgctttaagaacaaatgtatacttttagagagttc
		2		KL2<br>**ACACTCTTTCCCTACACGACGCTCTTCCGATCT**AGCTaatgctttaagaacaaatgtatacttttagagagttc
		3		KL3<br>**ACACTCTTTCCCTACACGACGCTCTTCCGATCT**GCACaatgctttaagaacaaatgtatacttttagagagttc
		4		KL4<br>**ACACTCTTTCCCTACACGACGCTCTTCCGATCT**CATCaatgctttaagaacaaatgtatacttttagagagttc
		5		KL5<br>**ACACTCTTTCCCTACACGACGCTCTTCCGATCT**ATGAaatgctttaagaacaaatgtatacttttagagagttc
		6		KL6<br>**ACACTCTTTCCCTACACGACGCTCTTCCGATCT**TGCGaatgctttaagaacaaatgtatacttttagagagttc
	KD403<br>Δ*recJ* Δ*xseA*	1	Au12<br>**GACTGGAGTTCAGACGTGTGCTCTTCCGATCT**ACCTAcaacaaggtcggttgtttgacg	KL1<br>**ACACTCTTTCCCTACACGACGCTCTTCCGATCT**TCGAaatgctttaagaacaaatgtatacttttagagagttc
		2		KL2<br>**ACACTCTTTCCCTACACGACGCTCTTCCGATCT**AGCTaatgctttaagaacaaatgtatacttttagagagttc

		3		KL3<br>**ACACTCTTTCCCTACACGACGCTCTTCCGATCT**GCACaatgctttaagaacaaatgtatacttttagagagttc
		4		KL4<br>**ACACTCTTTCCCTACACGACGCTCTTCCGATCT**CATCaatgctttaagaacaaatgtatacttttagagagttc
		5		KL5<br>**ACACTCTTTCCCTACACGACGCTCTTCCGATCT**ATGAaatgctttaagaacaaatgtatacttttagagagttc
		6		KL6<br>**ACACTCTTTCCCTACACGACGCTCTTCCGATCT**TGCGaatgctttaagaacaaatgtatacttttagagagttc
	KD403<br>Δ*recB* Δ*recJ*	1	Au10<br>**GACTGGAGTTCAGACGTGTGCTCTTCCGATCT**ATGCCcaacaaggtcggttgtttgacg	KL1<br>**ACACTCTTTCCCTACACGACGCTCTTCCGATCT**TCGAaatgctttaagaacaaatgtatacttttagagagttc
		2		KL2<br>**ACACTCTTTCCCTACACGACGCTCTTCCGATCT**AGCTaatgctttaagaacaaatgtatacttttagagagttc
		3		KL3<br>**ACACTCTTTCCCTACACGACGCTCTTCCGATCT**GCACaatgctttaagaacaaatgtatacttttagagagttc
		4		KL4<br>**ACACTCTTTCCCTACACGACGCTCTTCCGATCT**CATCaatgctttaagaacaaatgtatacttttagagagttc
		5		KL5<br>**ACACTCTTTCCCTACACGACGCTCTTCCGATCT**ATGAaatgctttaagaacaaatgtatacttttagagagttc
		6		KL6<br>**ACACTCTTTCCCTACACGACGCTCTTCCGATCT**TGCGaatgctttaagaacaaatgtatacttttagagagttc
High-throughput sequencing of spacers acquired during primed adaptation (Fig. S1C-D)	KD403	1	Au1<br>**GACTGGAGTTCAGACGTGTGCTCTTCCGATCT**GTCAGcaacaaggtcggttgtttgacg	KL1<br>**ACACTCTTTCCCTACACGACGCTCTTCCGATCT**TCGAaatgctttaagaacaaatgtatacttttagagagttc
		2	Au2<br>**GACTGGAGTTCAGACGTGTGCTCTTCCGATCT**GGTTGcaacaaggtcggttgtttgacg	

		3	Au3<br>**GACTGGAGTTCAGACGTGTGCTCTTCCGATCT**CCAGCcaacaaggtcggttgtttgacg	
		4	Au4<br>**GACTGGAGTTCAGACGTGTGCTCTTCCGATCT**TACCGcaacaaggtcggttgtttgacg	
		5	Au5<br>**GACTGGAGTTCAGACGTGTGCTCTTCCGATCT**TAGGAcaacaaggtcggttgtttgacg	
		6	Au6<br>**GACTGGAGTTCAGACGTGTGCTCTTCCGATCT**GCTCTcaacaaggtcggttgtttgacg	
	KD403<br>Δ*recB*	1	Au1<br>**GACTGGAGTTCAGACGTGTGCTCTTCCGATCT**GTCAGcaacaaggtcggttgtttgacg	KL2<br>**ACACTCTTTCCCTACACGACGCTCTTCCGATCT**AGCTaatgctttaagaacaaatgtatacttttagagagttc
		2	Au2<br>**GACTGGAGTTCAGACGTGTGCTCTTCCGATCT**GGTTGcaacaaggtcggttgtttgacg	
		3	Au3<br>**GACTGGAGTTCAGACGTGTGCTCTTCCGATCT**CCAGCcaacaaggtcggttgtttgacg	
		4	Au4<br>**GACTGGAGTTCAGACGTGTGCTCTTCCGATCT**TACCGcaacaaggtcggttgtttgacg	
		5	Au5<br>**GACTGGAGTTCAGACGTGTGCTCTTCCGATCT**TAGGAcaacaaggtcggttgtttgacg	
		6	Au6<br>**GACTGGAGTTCAGACGTGTGCTCTTCCGATCT**GCTCTcaacaaggtcggttgtttgacg	
	KD403<br>Δ*recQ*	1	Au1<br>**GACTGGAGTTCAGACGTGTGCTCTTCCGATCT**GTCAGcaacaaggtcggttgtttgacg	KL3<br>**ACACTCTTTCCCTACACGACGCTCTTCCGATCT**GCACaatgctttaagaacaaatgtatacttttagagagttc
		2	Au2<br>**GACTGGAGTTCAGACGTGTGCTCTTCCGATCT**GGTTGcaacaaggtcggttgtttgacg	
		3	Au3<br>**GACTGGAGTTCAGACGTGTGCTCTTCCGATCT**CCAGCcaacaaggtcggttgtttgacg	

		4	Au4 **GACTGGAGTTCAGACGTGTGCTCTTCC GATCT**TACCGcaacaaggtcggttgtttgacg	
		5	Au5 **GACTGGAGTTCAGACGTGTGCTCTTCC GATCT**TAGGAcaacaaggtcggttgtttgacg	
		6	Au6 **GACTGGAGTTCAGACGTGTGCTCTTCC GATCT**GCTCTcaacaaggtcggttgtttgacg	
	KD403 Δ*recB* Δ*recQ*	1	Au1 **GACTGGAGTTCAGACGTGTGCTCTTCC GATCT**GTCAGcaacaaggtcggttgtttgacg	KL4 **ACACTCTTTCCCTACACGACGCTCTTCCGATCT**C ATCaatgctttaagaacaaatgtatacttttagagagttc
		2	Au2 **GACTGGAGTTCAGACGTGTGCTCTTCC GATCT**GGTTGcaacaaggtcggttgtttgacg	
		3	Au3 **GACTGGAGTTCAGACGTGTGCTCTTCC GATCT**CCAGCcaacaaggtcggttgtttgacg	
		4	Au4 **GACTGGAGTTCAGACGTGTGCTCTTCC GATCT**TACCGcaacaaggtcggttgtttgacg	
		5	Au5 **GACTGGAGTTCAGACGTGTGCTCTTCC GATCT**TAGGAcaacaaggtcggttgtttgacg	
		6	Au6 **GACTGGAGTTCAGACGTGTGCTCTTCC GATCT**GCTCTcaacaaggtcggttgtttgacg	

High-throughput sequencing of spacers acquired during primed adaptation in KD403 and Δ*rnt*, Δ*dnaQ*, and Δ*rnt* Δ*dnaQ* derivatives (Fig. S8G). Illumina adapters were ligated using NEBNext Ultra II Library Prep Kit (NEB)	KD403, KD403 Δ*rnt*, KD403 Δ*dnaQ*, KD403 Δ*rnt* Δ*dnaQ*	1-3	LDR-F2 atgctttaagaacaaatgtatacttttag	autoSp2_R aatagcgaacaacaaggtcggttg
High-throughput sequencing of spacers acquired during prespacer efficiency assay in BL21-AI (Fig. S4C-D)	BL21-AI, oligo 33nt/37nt	1	BC-Ld1 **ACACTCTTTCCCTACACGACGCTCTTCCGATCT**TACTatcattaattaataataggttatgtttagagtg	BC-Sp **GACTGGAGTTCAGACGTGTGCTCTTCCGATCT**ttgagcgatgatatttgtgctcgg
		2		
		3		
		4		
		5		
		6		
	BL21-AI, oligo 32nt/36nt	1	BC-Ld2 **ACACTCTTTCCCTACACGACGCTCTTCCGATCT**AGACatcattaattaataataggttatgtttagagtg	
		2		
		3		
		4		
		5		
		6		
	BL21-AI, oligo 34nt/38nt	1	BC-Ld3 **ACACTCTTTCCCTACACGACGCTCTTCCGATCT**CTGAatcattaattaataataggttatgtttagagtg	
		2		
		3		
		4		
		5		
		6		
High-throughput sequencing of spacers acquired during prespacer efficiency assay in BL21-AI	BL21-AI Δ*recB* Δ*recJ*, oligo 33nt/37nt	1	BC-Ld1 **ACACTCTTTCCCTACACGACGCTCTTCCGATCT**TACTatcattaattaataataggttatgtttagagtg	
		2		
		3		
		4	BC-Ld2 **ACACTCTTTCCCTACACGACGCTCTTCCGATCT**AGACatcattaattaataataggttatgtttagagtg	
		5		
		6		

Δ*recB* Δ*recJ* (Fig. S4E-F)	BL21-AI Δ*recB* Δ*recJ*, oligo 32nt/36nt	1<br>2<br>3	BC-Ld1<br>**ACACTCTTTCCCTACACGACGCTCTTCCGATCT**TACTatcattaattaataataggttatgtttagagtg	
		4<br>5<br>6	BC-Ld2<br>**ACACTCTTTCCCTACACGACGCTCTTCCGATCT**AGACatcattaattaataataggttatgtttagagtg	
	BL21-AI Δ*recB* Δ*recJ*, oligo 34nt/38nt	1<br>2<br>3	BC-Ld1<br>**ACACTCTTTCCCTACACGACGCTCTTCCGATCT**TACTatcattaattaataataggttatgtttagagtg	
		4<br>5<br>6	BC-Ld2<br>**ACACTCTTTCCCTACACGACGCTCTTCCGATCT**AGACatcattaattaataataggttatgtttagagtg	
High-throughput sequencing of spacers acquired during prespacer efficiency assay in BL21-AI Δ*recJ* Δ*xseA* (Fig. S4G-H)	BL21-AI Δ*recJ* Δ*xseA*, oligo 33nt/37nt	1<br>2<br>3	BC-Ld3<br>**ACACTCTTTCCCTACACGACGCTCTTCCGATCT**CTGAatcattaattaataataggttatgtttagagtg	
		4<br>5<br>6	BC-Ld4<br>**ACACTCTTTCCCTACACGACGCTCTTCCGATCT**CATGatcattaattaataataggttatgtttagagtg	
	BL21-AI Δ*recJ* Δ*xseA*, oligo 32nt/36nt	1<br>2<br>3	BC-Ld3<br>**ACACTCTTTCCCTACACGACGCTCTTCCGATCT**CTGAatcattaattaataataggttatgtttagagtg	
		4<br>5<br>6	BC-Ld4<br>**ACACTCTTTCCCTACACGACGCTCTTCCGATCT**CATGatcattaattaataataggttatgtttagagtg	
	BL21-AI Δ*recJ* Δ*xseA*, oligo 34nt/38nt	1<br>2<br>3	BC-Ld3<br>**ACACTCTTTCCCTACACGACGCTCTTCCGATCT**CTGAatcattaattaataataggttatgtttagagtg	
		4<br>5<br>6	BC-Ld4<br>**ACACTCTTTCCCTACACGACGCTCTTCCGATCT**CATGatcattaattaataataggttatgtttagagtg	
High-throughput sequencing of spacers acquired during primed adaptation (Fig. S10F)	KD403, growing	1	Au1<br>**GACTGGAGTTCAGACGTGTGCTCTTCCGATCT**GTCAGcaacaaggtcggttgtttgacg	KL1<br>**ACACTCTTTCCCTACACGACGCTCTTCCGATCT**TCGAaatgctttaagaacaaatgtatacttttagagagttc
		2		KL2<br>**ACACTCTTTCCCTACACGACGCTCTTCCGATCT**AGCTaatgctttaagaacaaatgtatacttttagagagttc
		3		KL3<br>**ACACTCTTTCCCTACACGACGCTCTTCCGATCT**GCACaatgctttaagaacaaatgtatacttttagagagttc

		4		KL4<br>**ACACTCTTTCCCTACACGACGCTCTTCCGATCT**CATCaatgctttaagaacaaatgtatacttttagagagttc
		5		KL5<br>**ACACTCTTTCCCTACACGACGCTCTTCCGATCT**ATGAaatgctttaagaacaaatgtatacttttagagagttc
		6		KL6<br>**ACACTCTTTCCCTACACGACGCTCTTCCGATCT**TGCGaatgctttaagaacaaatgtatacttttagagagttc
	KD403, stationary	1	Au2<br>**GACTGGAGTTCAGACGTGTGCTCTTCCGATCT**GGTTGcaacaaggtcggttgtttgacg	KL1<br>**ACACTCTTTCCCTACACGACGCTCTTCCGATCT**TCGAaatgctttaagaacaaatgtatacttttagagagttc
		2		KL2<br>**ACACTCTTTCCCTACACGACGCTCTTCCGATCT**AGCTaatgctttaagaacaaatgtatacttttagagagttc
		3		KL3<br>**ACACTCTTTCCCTACACGACGCTCTTCCGATCT**GCACaatgctttaagaacaaatgtatacttttagagagttc
		4		KL4<br>**ACACTCTTTCCCTACACGACGCTCTTCCGATCT**CATCaatgctttaagaacaaatgtatacttttagagagttc
		5		KL5<br>**ACACTCTTTCCCTACACGACGCTCTTCCGATCT**ATGAaatgctttaagaacaaatgtatacttttagagagttc
		6		KL6<br>**ACACTCTTTCCCTACACGACGCTCTTCCGATCT**TGCGaatgctttaagaacaaatgtatacttttagagagttc

Table S21.
List of primers used for amplification of CRISPR arrays for high-throughput sequencing

Lowercase letters indicate nucleotides annealing to the chromosome.
NebNext Illumina adapter sequences are shown in bold. Additional barcodes introduced after adapter sequences are underlined.

Oligo name	Sequence (5' to 3')	Length, nt
IFASds31F	TCCCCTTGTCCAATCAAACTCATCGAATTCA	31
IFASds31R	TGAATTCGATGAGTTTGATTGGACAAGGGGA	
IFASds32F	TACATTTAAGACCCTAATATCACATCATTAGA	32
IFASds32R	TCTAATGATGTGATATTAGGGTCTTAAATGTA	
IFASds33F	CATTAATTGCCACTGCCAAAATTCTGTCCAGAA	33
IFASds33R	TTCTGGACAGAATTTTGGCAGTGGCAATTAATG	
IFASds34F	CGACTACCAAATCCGCATGTTAGGGGACTTCTTA	34
IFASds34R	TAAGAAGTCCCCTAACATGCGGATTTGGTAGTCG	
IFASds35F	TTAATTCTTTTATCGTGAGGAGCAGCGGATCTTAA	35
IFASds35R	TTAAGATCCGCTGCTCCTCACGATAAAAGAATTAA	
IFASds36F	TACTTATAAGATGTCTCAACGGTATCCGCAACTTGG	36
IFASds36R	CCAAGTTGCGGATACCGTTGAGACATCTTATAAGTA	
IFASds37F	CAAGTGCCTACTATCCTTAAACGCATATCTCGCCCAG	37
IFASds37R	CTGGGCGAGATATGCGTTTAAGGATAGTAGGCACTTG	
IFASds38F	TAGCTTCCCAATATGTGAGCATCAATTGTTGTCCGGCG	38
IFASds38R	CGCCGGACAACAATTGATGCTCACATATTGGGAAGCTA	
IFASds39F	CGAGATAGTCATGTGCTCACGGAACTTACTGTATGAGTA	39
IFASds39R	TACTCATACAGTAAGTTCCGTGAGCACATGACTATCTCG	
IFASds40F	CTGATTTGAAAGAGTTGTCAGTTTGCTGGTTCAGGTAAAG	40
IFASds40R	CTTTACCTGAACCAGCAAACTGACAACTCTTTCAAATCAG	

Table S22.

List of oligonucleotides used to determine the loss coefficients for fragments of various lengths
